# Comparative S-acyl-proteomics reveals post-translational modulation of fungal purine metabolism for Verticillium wilt

**DOI:** 10.1371/journal.ppat.1014730

**Published:** 2026-10-26

**Authors:** Chi Li, Wenyang Zhang, Shuaifei Cui, Jian Zhang, Yuzhen Li, Xiaoshi Liu, Yang Li, Zhiying Wang, Wenjie Huang, Gongda Li, Chengwei Yang, Shijuan Yan, Jianbin Lai

**Affiliations:** 1 Guangdong Provincial Key Laboratory of Biotechnology for Plant Development, School of Life Sciences, South China Normal University, Guangzhou, China; 2 Guangdong Key Laboratory for Crop Germplasm Resources Preservation and Utilization, Agro-biological Gene Research Center, Guangdong Academy of Agricultural Sciences, Guangzhou, China; University of Nebraska-Lincoln, UNITED STATES OF AMERICA

## Abstract

Verticillium wilt, caused by the fungus *Verticillium dahliae*, is a devastating plant disease leading to severe global agricultural losses. Although the interaction between *V. dahliae* and host plants has been extensively investigated, the role of metabolic regulation in fungal development and pathogenicity remains poorly understood. Fatty acids serve as metabolic substrates for S-acylation, a post-translational modification that modulates protein properties and functions, yet this process has not been characterized in *V. dahliae*. Here, our systematic analysis revealed that the protein S-acyltransferase VdERF2 is essential for the development and pathogenicity of *V. dahliae*. Through comparative S-acyl-proteomic analyses, we found that VdERF2-dependent S-acylation is significantly enriched in metabolic pathways, especially purine metabolism. Among the identified substrates, VdHIUH, an enzyme involved in converting uric acid to allantoin in purine metabolism, requires S-acylation for its full function in fungal development and virulence. VdERF2-mediated S-acylation enhances the activity of VdHIUH, thereby reducing uric acid accumulation. Furthermore, allantoin reduces reactive oxygen species elevated by the VdHIUH S-acylation defect, partially alleviating the fungal developmental impairment. Collectively, our findings reveal a critical mechanism by which S-acylation modulates purine metabolism in *V. dahliae*, providing new insights into the molecular basis of fungal pathogenicity and offering potential targets for improving crop resistance to Verticillium wilt.

## Author summary

Verticillium wilt, which is caused by *Verticillium dahliae*, is a important global plant disease. However, the role of metabolic regulation in the growth and pathogenicity of this plant pathogenic fungus remains unclear. S-acylation, is a key post-translational modification which transfers fatty acids to protein substrates, but it has not been characterized in *V. dahliae*. Here, we show that the protein S-acyltransferase VdERF2 is essential for *V. dahliae* development and virulence. Comparative S-acyl-proteomics reveals VdERF2-dependent S-acylation is enriched in metabolism-associated processes, especially purine metabolism. Among its substrates, VdHIUH, the enzyme converting uric acid to allantoin, requires S-acylation for full activity, supporting normal development and pathogenicity. VdERF2-mediated S-acylation enhances VdHIUH activity, lowering uric acid accumulation. Notably, exogenous allantoin partially alleviates developmental defects caused by impaired VdHIUH S-acylation in *V. dahliae*, likely by suppressing the accumulation of reactive oxygen species. Together, these findings uncover an S-acylation-purine metabolism axis governing *V. dahliae* virulence, and identify promising new targets for Verticillium wilt control.

## Introduction

A substantial number of environmental pathogens infect a diverse range of plant species, significantly impacting agricultural productivity. *Verticillium dahliae* is a soil-borne fungal pathogen that invades plant roots and subsequently colonizes the xylem vasculature, leading to Verticillium wilt [1]. This disease, characterized by a range of symptoms such as wilting, necrosis, stunted growth, chlorosis, and vein clearing, affects more than two hundred dicotyledonous plant species and results in serious agricultural losses [2]. For instance, Verticillium wilt severely undermines both the yield and quality of cotton, representing a major threat to the global cotton industry [3]. Therefore, *V. dahliae* has emerged as a significant plant pathogen that requires effective control measures to ensure sustainable crop production. While much research has focused on host defense mechanisms during infection, the regulation of fungal development and virulence remains to be further elucidated [4]. In particular, the role of fungal metabolic modulation in this process constitutes a critical unresolved issue.

In the interaction between fungi and plants, nutritional metabolites play a crucial role; for instance, fatty acids are recognized as key components involved in the fungal-plant crosstalk [5]. Research has predominantly focused on the metabolic functions of fatty acids, but they also serve as substrates for post-translational lipidation [6]. Notably, long-chain fatty acids such as palmitic acid can be covalently attached to the cysteine residues of target proteins, resulting in S-acylation, a modification also referred to as S-palmitoylation. S-acylation increases the hydrophobicity of proteins, thereby mediating their membrane localization, intracellular trafficking, and functional regulation [7]. Acyl proteomics approaches for the enrichment of S-acylated proteins have identified numerous protein substrates across various species [8], indicating that this post-translational modification is essential in eukaryotes.

Protein S-acylation is catalyzed by a conserved family of protein S-acyltransferases (PATs), which contain an Asp-His-His-Cys (DHHC) motif in a cysteine-rich domain [9]. The PAT-mediated protein S-acylation has been reported to play diverse functional roles across different species. For instance, multiple DHHC proteins are involved in the regulation of various human diseases, including cancer and neurodegenerative disorders [10]; in plant species, PATs have been shown to target specific protein substrates to modulate developmental processes and stress responses [11]. Nevertheless, the direct function of S-acylation in metabolism regulation remains a critical unresolved issue, even in mammalian cells [12]. Although the first PAT was initially identified in yeast cells [13], investigations into this modification in plant pathogenic fungi remain limited and have so far been reported only in a few species. FonPAT2 mediates the S-acylation of the AP-2 complex subunit to stabilize the pathogenicity of *Fusarium oxysporum* [14]; UvPfa4 modulates the mitogen-activated protein kinase signaling for the infection of *Ustilaginoidea virens* [15]. However, the functions of protein S-acylation in *V. dahliae* remains entirely unknown*.*

Therefore, we initiated a functional characterization of PATs in *V. dahliae* and found that VdERF2 is essential for its development and virulence. Comparative proteomic analysis revealed that components associated with purine metabolism were enriched among the S-acylated proteins regulated by VdERF2. Purines represent fundamental compounds essential for the construction of nucleic acids, energy carriers, signaling molecules, and cofactors [16]. Purine metabolism is tightly controlled and follows a conserved degradation pathway to uric acid, but the terminal metabolic products vary probably among species [17,18]. In fungi, upon uric acid degradation mediated by urate oxidase (also known as uricase), two unstable intermediates, 5-hydroxyisourate (HIU) and 2-oxo-4-hydroxy-4-carboxy-5-ureidoimidazoline (OHCU), are sequentially generated [19]. HIU hydrolase catalyzes the conversion of HIU to OHCU, while OHCU decarboxylase mediates the transformation of OHCU into allantoin [20,21]. However, the regulatory mechanism in this purine metabolic process is unclear across different species.

Here, we elucidated the role of VdERF2-dependent S-acylation of HIU hydrolase in *V. dahliae* (hereafter referred to as VdHIUH) in modulating its activity during purine metabolism for fungal development and pathogenesis. This discovery establishes a link between S-acylation and metabolic processes, offering valuable insights for improving the resistance of crops against Verticillium wilt.

## Results

**Systematic analysis revealed that VdERF2 is a PAT that is essential for the development and virulence of *V. dahliae*.**

Because protein S-acylation is catalyzed by PATs that are evolutionarily conserved in eukaryotic organisms [22], we initiated a functional characterization of PATs in *V. dahliae* to investigate whether S-acylation plays a critical role in regulating the development and pathogenicity of this plant pathogenic fungus. By comparing the PAT sequences in yeast, six candidate PATs, designated VdAKR1, VdSWF1, VdERF2, VdPFA3, VdPFA4, and VdPFA5, were identified in *V. dahliae*. All VdPAT members contained multiple transmembrane domains and possessed the conserved DHHC motif within a cysteine-rich region (S1A Fig; S2 Fig), thus exhibiting the canonical structural features characteristic of PATs. Further phylogenetic analysis revealed that these six VdPATs are conserved across a broad range of phytopathogenic fungi (S1B Fig).

To investigate the biological functions of VdPATs in *V. dahliae*, individual knockout mutants of the six VdPAT-encoding genes were generated through Agrobacterium-mediated transformation (S3A Fig), and the resulting mutant strains were verified by PCR analysis (S3B Fig). To evaluate their roles in fungal growth, these six *VdPAT* knockout mutants were cultured on potato dextrose agar (PDA) medium for 7 days. Compared with the wild-type (WT), deletion of either *VdERF2* or *VdAKR1* markedly reduced mycelial growth rates and caused distinct alterations in colony morphology, whereas the loss of *VdSWF1*, *VdPFA3*, *VdPFA4*, or *VdPFA5* did not significantly affect the phenotypic characteristics of *V. dahliae* on the medium (Fig 1A, 1B). Notably, most of these *VdPAT* genes contribute to the virulence of *V. dahliae*, albeit to varying extents (S4 Fig). Given that the *VdERF2* mutant exhibited the most severe defects in both developmental and pathogenicity, we focused on the functional characterization of VdERF2 in this study.

**Fig 1:**
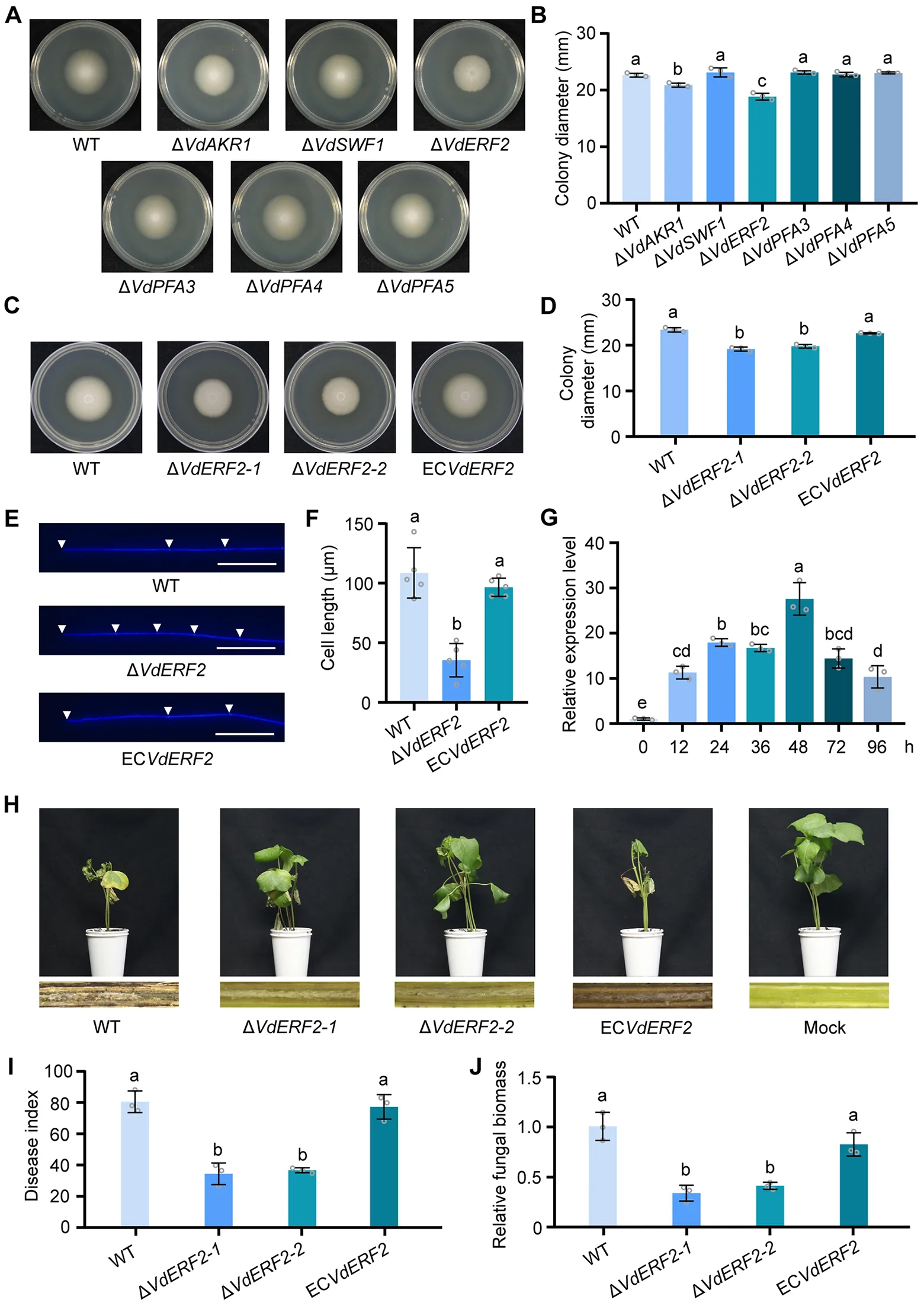
Systematic analysis revealed that VdERF2 is a PAT that is essential for the development and virulence of *V. dahliae.* **(A, B)** Colony growth phenotypes of *VdPAT* knockout mutants on PDA plates. Photographs taken 7 days after incubation are presented in (A). Colony diameters measured 7 days post-incubation (dpi) are shown in (B). **(C, D)** Colony growth phenotypes of the VdERF2 knockout and complemented strains on PDA plates. Representative images captured 7 dpi are shown in (C). Colony diameters, measured at 7 dpi, are presented in (D). **(E, F)** Hyphal tips of the VdERF2 mutant and complemented strains were stained with Calcofluor White. Representative microscopic images are presented in (E). Cell septa are indicated by white arrows. Bar = 50 µm. The statistical data for apical cell length at hyphal tips from five samples for each strain are shown in (F). **(G)** The expression pattern of *VdERF2* during the interaction between *V. dahliae* and cotton. Cotton samples inoculated with the WT *V. dahliae* strain were collected at different time points post-inoculation. Transcript levels of *VdERF2* were detected using quantitative RT-PCR. **(H-J)** The function of *VdERF2* in the virulence of *V. dahliae*. Representative disease symptoms in cotton plants infected with the indicated *V. dahliae* strains at 14 dpi are shown in (H). Disease indexes of inoculated cotton plants at 14 dpi are presented in (I). Relative fungal biomass in infected cotton plants (J) was measured by quantitative PCR, using *GhSSU* as the internal reference gene and *VdEF-1α* as the target gene. All results in this figure are representative of three biologically independent experiments. Data are expressed as means ± SD from three replicates. Statistical significance was determined by one-way ANOVA followed by Tukey’s multiple comparison test. Different letters indicate significant differences (*p* < 0.05).

To investigate the functional role of VdERF2 in *V. dahliae*, a complementation strain (EC*VdERF2*) was constructed in the Δ*VdERF2* mutant background. Analyses of two independent *VdERF2* knockout strains along with the EC*VdERF2* strain confirmed that VdERF2 is essential for normal mycelial growth and colony morphology of *V. dahliae* on PDA medium (Fig 1C, 1D). Because mycelial growth rate correlates with tip-cell length, we stained the apical regions of the WT, Δ*VdERF2*, and EC*VdERF2* strains with Calcofluor White. Microscopic analysis indicated that the tip cells in the Δ*VdERF2* strain were significantly shorter than those in the WT strain, and this defect was fully rescued in the EC*VdERF2* strain (Fig 1E, 1F). These findings indicate that VdERF2 regulates normal hyphal growth by controlling the development of hyphal tip cells in *V. dahliae*.

Because the development of *V. dahliae* may be linked to its virulence, we further investigated the impact of *VdERF2* depletion on fungal infection in cotton. First, we assessed the transcript levels of the *VdERF2* gene throughout the infection process. Quantitative RT-PCR analysis revealed that *VdERF2* expression gradually increased during infection, peaking at approximately 48 hours post-inoculation, followed by a slow decrease (Fig 1G), suggesting a potential role of this gene in the pathogenicity of *V. dahliae*. When cotton plants were inoculated with the WT or EC*VdERF2* strains, they exhibited severe disease symptoms, including wilting, chlorosis, necrosis, defoliation, and vascular browning. In contrast, infection with two independent Δ*VdERF2* mutant strains resulted in markedly milder symptoms (Fig 1H). Consistently, both disease index and fungal biomass measurements confirmed that VdERF2 functions as a critical virulence factor for *V. dahliae* during cotton infection (Fig 1I, 1J).

### Comparative S-acyl-proteomics analysis revealed the regulatory role of VdERF2 in the purine metabolism pathway

Because the aforementioned data demonstrated that ERF2 plays a crucial role in the development and pathogenicity of *V. dahliae*, and its homolog has not yet been functionally characterized in other phytopathogenic fungi, this system based on Δ*VdERF2* offers an ideal platform for investigating the specific regulatory mechanisms of protein S-acylation in plant pathogens. Proteomics based on the biotin-switch assay have been utilized to characterize S-acylated proteins in eukaryotic cells [23]. Therefore, a comparative proteomic approach employing this method was applied to identify protein substrates specifically mediated by ERF2 in *V. dahliae* (Fig 2A). Total proteins were extracted from both WT and Δ*VdERF2* strains, and free thiols in the cysteine residues of the proteins were blocked with N-ethylmaleimide (NEM). Hydroxylamine (NH_2_OH) was subsequently used to specifically cleave thioester bonds between fatty acyl groups and cysteine residues (Tris-HCl was used in negative control samples), and the newly exposed thiols were labeled with HPDP-biotin. The biotin-labeled proteins were then affinity-enriched using streptavidin agarose and eluted with β-mercaptoethanol. The eluted proteins represented S-acylated proteins for further analysis.

**Fig 2:**
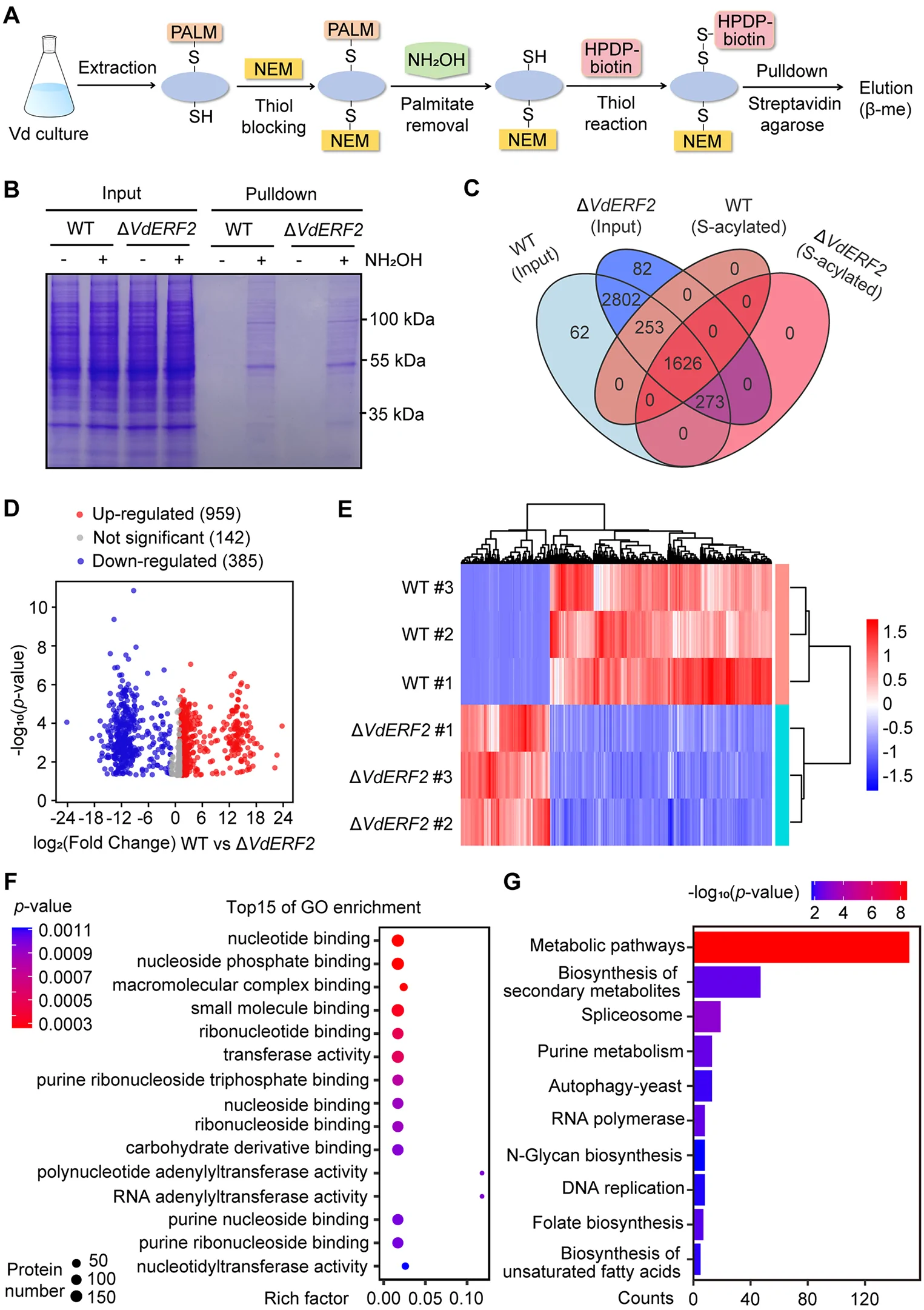
Comparative S-acyl-proteomics analysis revealed the regulatory role of VdERF2 in the purine metabolism pathway. **(A)** Schematic representation of the biotin-switch assay for proteomic analysis of S-acylated proteins from *V. dahliae*. SH, free thiol groups on cysteine residues; PALM, palmitoyl group. **(B)** SDS-PAGE analysis of S-acylated proteins from the WT and Δ*VdERF2* strains. The representative Coomassie blue staining result is presented. The NH_2_OH+ pulldown samples represent S-acylated proteins enriched through the biotin-switch assay. The NH_2_OH– pulldown samples indicate the negative backgrounds of enrichment. **(C)** Summary of the numbers of total proteins and S-acylated proteins identified by quantitative proteomics of the WT and Δ*VdERF2* strains in a Venn diagram. **(D)** Comparative analysis of S-acylated proteins between WT and Δ*VdERF2* strains. The up-regulated, not significant, and down-regulated groups in the volcano plot represent the ratios of protein S-acylation levels (WT/Δ*VdERF2*). **(E)** Heat map illustrating cluster analysis of differentially S-acylated proteins in *V. dahliae* comparing the WT and Δ*VdERF2* strains. The data from three biologically independent experiments are presented. **(F)** GO analysis of VdERF2-mediated S-acylated proteins in *V. dahliae*. The GO enrichment of molecular functions for the upregulated group of S-acylated proteins (WT/Δ*VdERF2*) is presented, showing the top15 enriched terms. **(G)** KEGG analysis of proteins undergoing VdERF2-dependent S-acylation. KEGG pathway enrichment was analyzed on the upregulated group of S-acylated proteins (WT/Δ*VdERF2*), and the top10 significantly enriched terms are presented.

SDS-PAGE analysis of the eluted proteins revealed a much higher enrichment of proteins in samples treated with NH_2_OH compared to those without NH_2_OH, indicating the specificity of this assay for capturing S-acylated proteins. Notably, S-acylated proteins were still detected in the sample from Δ*VdERF2* cells (Fig 2B), suggesting that this enzyme may be responsible only for the S-acylation of specific protein substrates. Therefore, three biological replicates of input and pulldown samples from both WT and Δ*VdERF2* strains were subjected to LC-MS/MS analysis. The LC-MS/MS analysis identified 5,016 proteins in the input samples of the WT strain and 5,036 in the Δ*VdERF2* strain, with 4,954 proteins common to both groups (Fig 2C; S5A Fig; S1 Dataset). In the pulldown samples for S-acylated proteins, 1,879 proteins were identified in the WT and 1,899 in the Δ*VdERF2* strain, with 1,626 proteins common to both datasets (Fig 2C; S1 Dataset). Following rigorous filtering, normalization, and statistical analysis, a total of 1,344 proteins were identified with differential S-acylation levels between the WT and Δ*VdERF2* strains. Among these, 959 proteins exhibited up-regulation and 385 showed down-regulation in the WT relative to the Δ*VdERF2* mutant (Fig 2D; S1 Dataset), indicating that VdERF2 plays a positive role in regulating the S-acylation of the majority of these modified proteins. Hierarchical clustering of the differentially modified proteins revealed tight clustering among the three biological replicates in each genotype and a clear separation between the WT and Δ*VdERF2* samples, underscoring the robustness of the comparison (Fig 2E; S2 Dataset).

Because the up-regulated S-acylated proteins were potential targets of VdERF2, we focused on the proteins in this group. Gene Ontology (GO) classification revealed that these up-regulated S-acylated proteins are enriched in multiple biological processes such as “cellular metabolic process” (S5B, S5C Fig; S2 Dataset), as well as in molecular functions associated with “nucleotide binding”, “nucleoside binding”, “purine nucleotide binding”, and “purine ribonucleotide binding” (Fig 2F). Consistently, KEGG pathway analysis of the up-regulated modified proteins revealed significant enrichment in “metabolic pathways” including “purine metabolism” (Fig 2G; S2 Dataset). These bioinformatics analyses indicate that purine metabolism is a targeted process by VdERF2-dependent S-acylation in *V. dahliae*.

### S-acylation of a HIU hydrolase mediated by VdERF2 is essential for the development and virulence of *V. dahliae*

Our proteomics data indicated that VdERF2-mediated protein S-acylation is associated with purine metabolism, a critical yet poorly understood metabolic pathway in *V. dahliae*. Therefore, we initiated a search for potential targets in the differentially S-acylated protein group to elucidate the regulatory mechanism underlying this process. Among these candidates, we selected VdHIUH, a conserved and essential enzyme involved in purine and uric acid metabolism, for further analysis. VdHIUH was fused with GFP and expressed in both the WT and Δ*VdERF2* strains of *V. dahliae*. Proteins were subsequently extracted for the biotin-switch assay, which revealed that VdHIUH underwent S-acylation in WT *V. dahliae* cells, and this modification was markedly reduced in the Δ*VdERF2* strain (Fig 3A). This biochemical evidence supported the conclusion that S-acylation of VdHIUH was mediated by VdERF2 and confirmed the reliability of our proteomics data. Moreover, co-immunoprecipitation confirmed an interaction between VdERF2 and VdHIUH (S6 Fig), thereby supporting their functional association as a PAT-substrate pair. Based on bioinformatics predictions via CSS-Palm [24] indicating that the C9 residue represents a potential S-acylation site in VdHIUH, we generated a point mutation substituting this cysteine with serine (C9S). Biotin-switch assay revealed that the C9S mutation dramatically reduced the modification of VdHIUH (Fig 3B). The persistence of a weak S-acylation signal in the C9S mutant protein suggests the presence of additional potential S-acylation sites; however, C9 is the primary S-acylation site of VdHIUH. Sequence alignment indicated that VdHIUH homologs are widely distributed among plant-pathogenic fungi, with the N-terminal cysteine residue exhibiting conservation across these species (S7 Fig).

**Fig 3:**
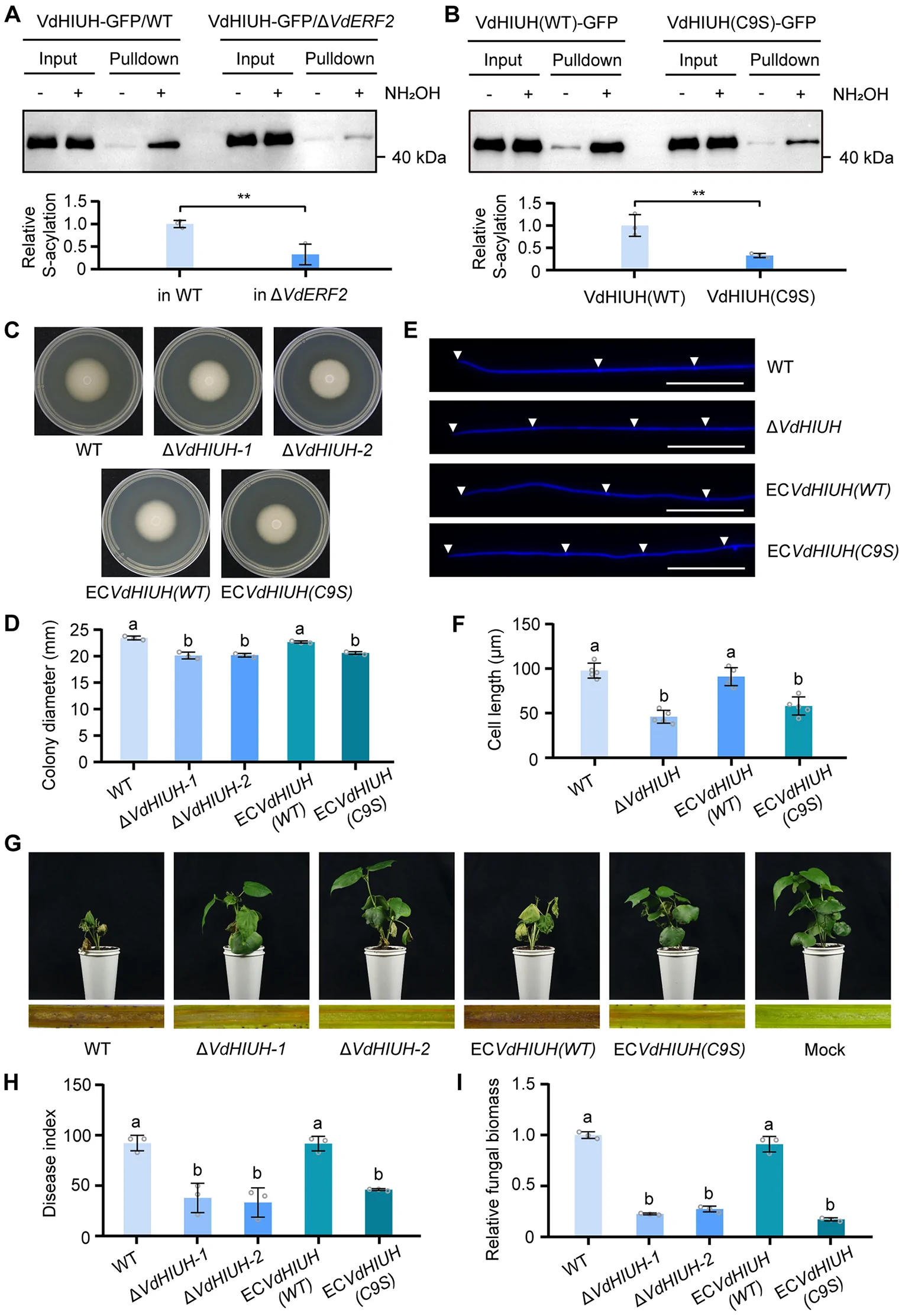
S-acylation of a HIU hydrolase mediated by VdERF2 is essential for the development and virulence of *V. dahliae.* **(A)** Detection of S-acylation of VdHIUH-GFP in the WT and Δ*VdERF2* strains. The S-acylation levels of VdHIUH-GFP from the indicated strains were assessed using a biotin-switch assay. A representative anti-GFP immunoblot is shown in the top graph. Quantitative analysis of S-acylation levels is presented in the bottom graph. **(B)** Identification of the S-acylation site on VdHIUH. The WT and C9S forms of VdHIUH-GFP from *V. dahliae* were analyzed in for the biotin-switch assay. A representative anti-GFP immunoblot is shown in the top graph. Statistical S-acylation levels are presented in the bottom graph. Data in (A) and (B) represent means ± SD from immunoblot signals ([pulldown+/input+] – [pulldown–/input–]) of biologically independent experiments. The relative S-acylation level of the WT version of VdHIUH-GFP from the WT strain was normalized to 1. Statistical significance was assessed by Student’s *t*-test (**p < 0.01). **(C, D)** Role of VdHIUH and its S-acylation in regulating colony growth. The representative colony phenotypes of the WT, Δ*VdHIUH* mutant, and complemented strains (WT or C9S versions) on PDA plates at 7 dpi are shown in (C). The quantified colony diameters measured at 7 dpi are presented in (D). **(E, F)** The impact of VdHIUH and its S-acylation on the cell length of apical cells at hyphal tips. In (E), hyphal tips of the indicated strains were stained with Calcofluor White, and cell septa are marked by white arrows. Bar = 50 µm. Quantified cell lengths from five samples for each strain are shown in (F). **(G-I)** Role of VdHIUH and its S-acylation in the virulence of *V. dahliae*. Representative symptoms of cotton plants inoculated with the indicated *V. dahliae* strains at 14 dpi are shown in (G). Disease index values for the inoculated cotton plants at 14 dpi are presented in (H). Relative fungal biomass in (I) was quantified using quantitative PCR, with *GhSSU* serving as the internal reference gene and *VdEF-1α* as the target gene. All data shown in this figure are representative of three biologically independent experiments. Quantitative results are expressed as means ± SD from three replicates. Statistical significance was assessed by one-way ANOVA followed by Tukey’s multiple comparison test. Different letters denote statistically significant differences (*p* < 0.05).

To investigate the function of VdHIUH and the role of its S-acylation, we generated Δ*VdHIUH* knockout mutant strains (S3C Fig). Subsequently, the WT or C9S mutant version of *VdHIUH* was introduced into the Δ*VdHIUH* mutant background to generate the complemented strains EC*VdHIUH(WT)* and EC*VdHIUH(C9S)*, respectively. After seven days of growth on PDA medium, two independent Δ*VdHIUH* knockout strains exhibited markedly aberrant colony morphology, with significantly reduced colony diameters compared to the WT strain. This abnormal phenotype was fully rescued in the EC*VdHIUH(WT)* strain, whereas the EC*VdHIUH(C9S)* strain continued to display defective morphology (Fig 3C, 3D). Consistently, Calcofluor White staining of apical cells revealed that both the Δ*VdHIUH* and *ECVdHIUH(C9S)* strains produced significantly shorter hyphal tip cells than the WT strain, while the tip-cell length in the EC*VdHIUH(WT)* strain was comparable to that of the WT strain (Fig 3E, 3F). These phenotypic analyses support the conclusion that VdHIUH is essential for normal developmental progression and that its S-acylation is critical for this function.

To evaluate the role of VdHIUH and its S-acylation in the pathogenesis of *V. dahliae*, cotton seedlings were inoculated with the WT, Δ*VdHIUH*, EC*VdHIUH(WT)*, or EC*VdHIUH(C9S)* strains. Cotton plants infected with the Δ*VdHIUH* mutant strains exhibited significantly reduced disease symptoms, along with lower disease index values and decreased fungal biomass, compared to those inoculated with the WT strain (Fig 3G–I), indicating that this enzyme plays a crucial role in the virulence of *V. dahliae*. Notably, the disease symptoms induced by EC*VdHIUH(WT)* were comparable to those caused by the WT strain, whereas EC*VdHIUH(C9S)* resulted in a similar phenotypic effect on cotton plants (Fig 3G–I). These inoculation results demonstrate that both VdHIUH and its S-acylation are essential for the full pathogenicity of *V. dahliae* on host plants.

### VdERF2-dependent S-acylation modulates VdHIUH activity in purine degradation for the development of *V. dahliae*

Given that HIU hydrolases catalyze the conversion of 5-HIU to OHCU, a critical step in uric acid metabolism downstream of purine degradation [25] (Fig 4A), the S-acylation of VdHIUH may serve as a regulatory mechanism for this metabolic pathway. To investigate this possibility, we analyzed the effect of abolishing VdHIUH S-acylation on uric acid accumulation in *V. dahliae*. The results showed that uric acid levels were significantly higher in the Δ*VdHIUH* mutant strain compared to the WT strain, supporting the functional role of VdHIUH in this metabolic process. Moreover, elevated uric acid levels were also observed in the Δ*VdERF2* and EC*VdHIUH(C9S)* strains relative to the EC*VdERF2* and EC*VdHIUH(WT)* strains (Fig 4B). Collectively, these findings indicate that VdERF2-mediated S-acylation of VdHIUH is essential for the efficient degradation of uric acid in *V. dahliae*.

**Fig 4:**
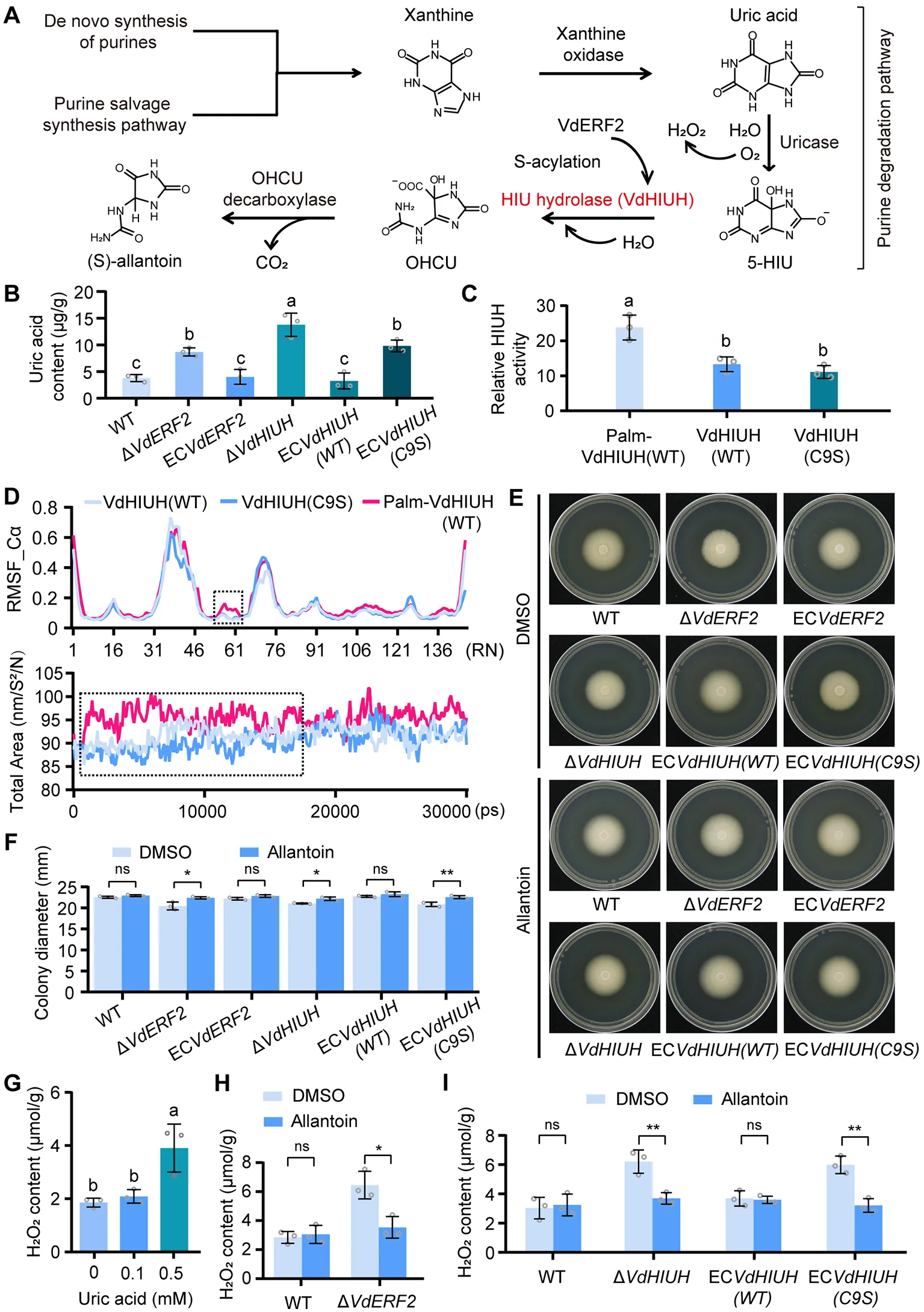
VdERF2-dependent S-acylation modulates VdHIUH activity in purine degradation for the development of *V. dahliae.* **(A**) The diagram illustrating the purine degradation pathway in *V. dahliae* cells. **(B)** Determination of uric acid content in the WT, Δ*VdERF2*, Δ*VdHIUH*, and complementary strains. **(C)** The effect of S-acylation on VdHIUH enzymatic activity. Uric acid was incubated with uricase to generate 5-HIU. Subsequently, equal amounts of Palm-VdHIUH(WT)-GFP (purified from WT cells), VdHIUH(WT)-GFP (purified from Δ*VdERF2* cells), or VdHIUH(C9S)-GFP (purified from WT cells) were added to the reaction. Absorbance profiles at 312 nm were recorded and the relative HIU hydrolase activity was calculated. **(D)** Molecular dynamics simulations of VdHIUH with and without S-acylation. A palmitoyl group was attached to the C9 residue of the VdHIUH protein (Palm-VdHIUH) to evaluate the impact of S-acylation on its structure. The VdHIUH(WT) protein lacking S-acylation and the VdHIUH(C9S) mutant protein were employed for comparative analysis. The root mean square fluctuations (RMSFs) are illustrated in the top graph. RN: residue number. The time-dependent evolution of the solvent-accessible surface area and the total accessible surface area are presented in the bottom graph. **(E, F)** The effect of allantoin on the growth of *V. dahliae* strains. Representative colony phenotypes of the WT, Δ*VdERF2*, EC*VdERF2*, Δ*VdHIUH*, EC*VdHIUH(WT)*, and EC*VdHIUH(C9S)* strains grown on PDA plates supplemented with 0.4 mM allantoin or DMSO solvent (control) at 7 dpi are shown in (E). Colony diameters of the indicated strains at 7 dpi are quantified in (F). **(G-I)** The impact of allantoin on the ROS status in *V. dahliae* strains. The H_2_O_2_ levels in the indicated *V. dahliae* strains, with different concentrations of uric acid treatment, are presented in (G). The influence of allantoin on H_2_O_2_ accumulation is shown in the Δ*VdERF2* strain (H) and in the Δ*VdHIUH* mutant and its complemented strains (I), relative to the WT strain. All results in this figure are representative of three biologically independent experiments. Quantitative data are means ± SD from three replicates. Statistical significance was assessed by Student’s *t*-test (*p < 0.05, **p < 0.01, ns. *p* > 0.05) or by one-way ANOVA followed by Tukey’s multiple comparison test. Different letters denote statistically significant differences (*p* < 0.05).

The next unresolved question was how S-acylation regulates this process. Given that the C9S mutation did not affect the stability or subcellular localization of VdHIUH-GFP (S8A–S8C Fig), we focused on whether this modification modulates its enzymatic activity. VdHIUH functions as the hydrolase responsible for catalyzing the degradation of the unstable intermediate 5-HIU in uric acid metabolism. To investigate its enzymatic activity, we reconstituted this metabolic pathway by incorporating recombinant *V. dahliae* uricase, which was expressed and purified from *E. coli*, along with HIUH-GFP from *V. dahliae*. The enzymatic conversion of 5-HIU was monitored by measuring changes in absorbance by spectrophotometry, thereby enabling the assessment of HIUH activity [26] (S8D, S8E Fig). As a result, the relative activity of S-acylated VdHIUH(WT)-GFP derived from the WT background strain was significantly higher than that of either VdHIUH(WT)-GFP from the Δ*VdERF2* background strain or VdHIUH(C9S)-GFP from the WT background strain (Fig 4C). Consistently, overexpression of VdHIUH partially rescued the growth and pathogenicity defects observed in the Δ*VdERF2* mutant (S9 Fig), suggesting that elevating the VdHIUH protein level may compensate for its diminished activity in this background. These findings support the hypothesis that S-acylation positively regulates the enzymatic activity of VdHIUH.

To investigate the potential mechanism by which S-acylation influences the activity of VdHIUH, the structural impact of palmitate attachment at the C9 residue was analyzed using molecular dynamics (MD) simulations [27]. When the C9 residue was mutated to serine, the overall protein structure remained nearly identical to that of WT VdHIUH, confirming that the functional consequences of this mutation were not attributable to structural alterations. Notably, MD simulations revealed that S-acylation of VdHIUH at the C9 residue (C9-palm) led to a distinct structural state compared to the unmodified protein. Due to the high hydrophobicity of palmitate, its attachment may perturb the secondary structure and alter the distribution of hydrophilic and hydrophobic surface accessibility in VdHIUH (Fig 4D), thereby modulating enzymatic activity.

Because uric acid is degraded to allantoin in purine metabolism in fungal cells, the S-acylation of VdHIUH may be functionally linked to allantoin. Therefore, we examined the effect of exogenous allantoin treatment on the growth of *V. dahliae* strains with different genotypes cultured on PDA medium. The results showed that allantoin supplementation had no significant effect on the growth of the WT, *ECVdERF2*, or EC*VdHIUH(WT)* strains, but it partially alleviated the growth defects observed in the Δ*VdERF2*, Δ*VdHIUH*, and EC*VdHIUH(C9S)* mutants (Fig 4E, 4F). These findings indicate that the impairment in purine degradation caused by the loss of VdERF2-mediated S-acylation of VdHIUH can be compensated by allantoin, the terminal product of this metabolic pathway. Previous studies have demonstrated an association between uric acid accumulation and reactive oxygen species (ROS) burst, which can impair organismal development. Given the elevated levels of uric acid observed in strains lacking VdHIUH S-acylation mediated by VdERF2, ROS accumulation may underlie their developmental defects. Our measurements confirmed that exogenous application of uric acid increased H_2_O_2_ levels in *V. dahliae* (Fig 4G). Compared to the WT strain, H_2_O_2_ levels were significantly elevated in the Δ*VdERF2*, Δ*VdHIUH*, and EC*VdHIUH(C9S)* strains; notably, this increase was completely abolished upon treatment with allantoin (Fig 4H, 4I). Consistently, in planta results demonstrate that application of allantoin functionally compensates for the loss of VdERF2 and VdHIUH during pathogenesis (S10 Fig). These findings elucidate the mechanism by which VdERF2-dependent S-acylation precisely regulates VdHIUH enzymatic activity in purine catabolism, thereby contributing to *V. dahliae* development and pathogenicity, potentially via maintenance of ROS homeostasis.

## Discussion

Metabolism is an essential biological process in all organisms, but the role of metabolic regulation in pathogens during their interaction with host cells remains poorly understood. In our current study, we uncover a mechanism by which the regulation of purine metabolism, mediated through protein S-acylation, contributes to the development and virulence of *V. dahliae* for Verticillium wilt. Verticillium wilt is a significant fungal disease affecting plants, yet the regulatory mechanisms governing the biology of the pathogen itself remain to be elucidated [4]. Previous studies have demonstrated that post-translational modifications play a crucial role in regulating the pathogenicity of *V. dahliae*. For instance, VdUlpB facilitates the de-SUMOylation of the glycolytic enzyme VdEno, thereby suppressing the expression of the effector VdSCP8 and enhancing fungal virulence [28]. Our study represents the first comprehensive investigation into the role of S-acylation, another critical protein modification, in modulating the development and pathogenicity of *V. dahliae*. This metabolic regulation strategy mediated via S-acylation in plant pathogenic fungi may also offer valuable clues for future investigations into human infectious diseases.

Through systematic characterization, we identified VdERF2 as a pivotal PAT regulating development and pathogenicity in *V. dahliae*, although other members of the PAT family may exhibit functional redundancy. ERF2 is evolutionarily conserved across fungal species, and its homolog in *Saccharomyces cerevisiae* represents the first identified PAT protein among all eukaryotic species [13]. In *S. cerevisiae*, ERF2 acts with its partner ERF4 to mediate the S-acylation of Ras, a small GTP-binding protein, thereby regulating signal transduction in multiple cellular processes [29,30]. In this study, comparative proteomic analysis revealed that proteins undergoing the VdERF2-dependent S-acylation are associated with purine metabolism, highlighting the regulatory specificity of this enzyme. Previous studies have demonstrated that S-acylation predominantly modulates key signaling transduction components, such as receptors and kinases [7]. Although S-acylation has also been implicated in human metabolic processes [31], for instance, by modifying a fatty acid translocase CD36 to indirectly influences β-oxidation [32], investigation into its direct targeting to metabolic enzymes remains limited in all species. Our results substantiate that VdHIUH is a critical substrate that partially mediates the biological function of VdERF2; however, the functional characterization of other candidate substrates remains an important objective for future studies.

Our analysis revealed that VdERF2 directly regulates VdHIUH, an enzyme that catalyzes the conversion of HIU to OHCU. The accumulation of uric acid observed in the Δ*VdERF2* and Δ*HIUH* mutants confirms the involvement of this module in the purine metabolic pathway of *V. dahliae*. Depletion of uric acid oxidation in mice results in mortality due to high levels of uric acid in the blood and kidney [33]. Interestingly, HIU hydrolase plays a subtle role in the virulence of the fungal pathogen *Cryptococcus neoformans* toward the nematode *Caenorhabditis elegans* [21]. These evidence suggests that uric acid metabolism is associated with multiple biological processes. Therefore, HIUH represents an ideal S-acylation target in the regulation of metabolism-associated development in *V. dahliae*. The hydrophobicity conferred by palmitoyl groups usually facilitates the association of proteins with membranes [34]. Although S-acylation may modulate enzyme activity, for instance, enhancing the kinase activity of human GRK6, this regulatory effect may be linked to changes in their membrane localization [35]. Our data indicated that S-acylation does not influence the subcellular localization of HIUH, but instead modulates its enzymatic activity. This effect may occur through the introduction of hydrophobic moieties, which alter the environment inside the enzyme, suggesting a general and direct mechanism by which S-acylation regulates enzyme activity in eukaryotic metabolism. Given that homologs of both VdERF2 and VdHIUH are widely distributed across plant-pathogenic fungi, it remains to be determined whether this VdERF2-VdHIUH module represents an evolutionarily conserved virulence mechanism.

Dysregulation of purine metabolism may impair cellular function through alterations in nutrient availability and metabolic products. The accumulation of uric acid resulting from disorders in purine metabolism can impose physiological stress. For instance, uric acid crystallization is associated with gout in humans and the concurrent increase of ROS represents a critical contributing factor [36,37]. HIUH is involved in the conversion of uric acid into allantoin, an antioxidant compound [38]. Our treatment with allantoin suppresses the developmental defects and ROS accumulation observed in the Δ*VdERF2* and Δ*HIUH* mutants, as well as the EC*HIUH(C9S)* strain, confirming that ROS accumulation is a cause of their abnormal development and support the conclusion that VdERF2 influences the development of *V. dahliae* through S-acylation of HIUH to modulate purine metabolism. The resulting developmental impairments compromise the ability of these strains to infect host plants. Notably, during infection, ROS accumulation occurs in the extracellular regions of host cells [39], highlighting a potential interplay between host-derived ROS and fungal purine metabolism. However, given that loss of VdHIUH, or its VdERF2-dependent S-acylation, blocks the metabolic flux from uric acid to allantoin and thereby prevents the utilization of purine-derived nitrogen, an alternative mechanism rooted in nitrogen nutrition remains plausible and warrants more detailed investigation in future studies. Furthermore, S-acylation is regulated by fatty acid homeostasis [40], thus it would be of interest to investigate its role in the crosstalk between fatty acid and nucleotide metabolism.

In summary, this study elucidates a mechanism through which protein S-acylation regulates purine metabolism in a plant-pathogenic fungus. It addresses a key question regarding how metabolic regulation contributes to the development and virulence of fungal pathogens. Consequently, S-acylation emerges as a promising target for inhibiting *V. dahliae*, offering a potential strategy to enhance crop resistance to Verticillium wilt. Given the evolutionary conservation of both S-acylation and metabolism pathways, these findings may also provide valuable insights for the controlling other pathogenic fungi and treating human disorders related to purine metabolism.

## Materials and methods

### Strains and plant growth conditions

The *V. dahliae* strain was cultured on PDA medium at 25 °C in the dark. *Escherichia coli* DH5α was used for plasmid construction and cultivated in LB medium at 37 °C. *Agrobacterium tumefaciens* EHA105 was employed for *A. tumefaciens*-mediated transformation (ATMT) and grown in LB medium at 28 °C. The susceptible cotton cultivar *Gossypium hirsutum* was maintained in a greenhouse under a 16-h light/8-h dark photoperiod at 25 ± 1 °C.

### Generation of mutant and complemented strains

The genomic DNA of *V. dahliae* was extracted using the CTAB method. The upstream and downstream flanking sequences of *VdPAT*s and *VdHIUH* were amplified by PCR from the genomic DNA and cloned into the *pGKO* vector using a Basic Seamless Cloning and Assembly Kit (TransGen Biotech, China). The resulting constructs were transformed into *A. tumefaciens* strain EHA105 (Weidi Biotechnology, China) and used for ATMT [41] to generate the Δ*VdPAT*s and Δ*VdHIUH* mutant strains. For complementation, the genomic region of *VdERF2 and VdHIUH*, including 1,400–1,500 bp upstream of their respective start codons, were amplified and cloned into the *pSul-GFP* vector to generate complementation constructs. These constructs were then transformed into the corresponding mutant strains via ATMT to obtain the complemented strains. Using a similar approach, the coding sequence fragments of VdHIUH(WT) or VdHIUH(C9S) were cloned into the *pSul-GFP* vector to generate the overexpression strains. All primers used in this study are listed in S3 Dataset.

### Virulence assays

Cotton seedlings were inoculated with *V. dahliae* using the root-dip method [42]. A conidial suspension of 1 × 10^7^ conidia/mL from the designated strain was used as the inoculum. Disease symptoms in the inoculated plants were assessed 14 days post-inoculation. The disease severity was classified as follows: Grade 0, no symptoms; Grade 1, 0–25% of leaves with symptoms exhibiting wilting, yellowing, and necrosis; Grade 2, 25–50% of leaves with symptoms; Grade 3, 50–75% of leaves with symptoms; and Grade 4, 75–100% of leaves with symptoms. The disease index was calculated as 100 × (Σ [number of plants × disease grade]) / ([total number of plants] × [maximum disease grade]).

### The biotin-switch assay

The biotin-switch assay was performed as previously described [43]. *V. dahliae* was cultured in liquid medium for 72 hours, after which mycelium and spores were collected and lysed using Lysis buffer (1 M Tris-HCl, 3 M NaCl, 0.5% Triton X-100, and 1× protease inhibitor) to prepare total proteins. Total proteins were blocked overnight at 4 °C with 2 M N-ethylmaleimide (NEM). Following the blocking step, methanol and chloroform were added to each tube, and the samples were centrifuged at 4 ℃ and 12000 *g* for 10 minutes. The resulting protein pellets were resuspended in 200 μL of Resuspension buffer (8 M urea, 2% SDS, and 1× PBS pH 7.4), and the protein solutions were equally divided into two tubes. To each tube, 50 μL of 4 mM biotin-HPDP was added, along with either 50 μL of 2 M Tris–HCl (pH 7.4) or 50 μL of 2 M NH_2_OH (pH 7.4). The mixtures were incubated for 1 hour, and then proteins were recovered by methanol-chloroform precipitation, followed by resuspension in 150 μL of Resuspension buffer. Next, 40 μL of each resuspended sample was retained as the input, and the remainder was mixed with 990 μL of PBS (pH 7.4) with 0.2% Triton X-100. The samples were further incubated with streptavidin-agarose for 1.5 hours. After incubation, the agarose was rinsed with Wash buffer (500 mM NaCl, 0.1% SDS, and 1× PBS pH 7.4). S-acylated proteins were eluted by incubating the agarose in 70 μL of Wash buffer supplemented with 5% β-mercaptoethanol for 20 minutes. The eluted protein samples were subjected to immunoblotting analysis using a anti-GFP antibody (TransGen Biotech, HT801–01) or to mass spectrometry analysis.

### The preparation of protein samples prior to mass spectrometry analysis

Proteins from the samples were mixed with NaCl to achieve a final concentration of 100 mM. Subsequently, four volumes of acetone were added to the mixture for precipitation at room temperature for 0.5 to 1 hour. The sample was then centrifuged at 8,000 *g* at 4 °C for 10 minutes. The supernatant was carefully removed and the pellet was washed it once with 1 mL of acetone. Following a second centrifugation at 8,000 *g* at 4 °C for 10 minutes, the supernatant was discarded, and the protein pellet was retained. The pellet was air-dried in a fume hood for 5–10 minutes and subsequently resuspended in a solution containing 1% SDS, 10 mM TCEP, and 40 mM CAA. The sample was vortexed, subjected to ultrasonic dissolution, and incubation at 80 °C for 5 minutes. After an additional round of acetone precipitation, the sample was prepared for enzymatic digestion.

Following this step, the protein pellet was resuspended in 50 μL of 50 mM ammonium bicarbonate. Trypsin (Promega, V5113) was added at an enzyme-to-protein ratio of 1:50 (w/w), and digestion was allowed to proceed overnight at 37 °C with intermittent vortexing. After digestion, formic acid was added to yield a final concentration of 1%, and the peptide sample was freeze-dried prior to mass spectrometric analysis.

### Liquid chromatography-tandem mass spectrometry analysis

The peptide sample was reconstituted in 0.1% formic acid and loaded onto a C18 trap column via an autosampler. Separation was performed on a C18 analytical column (75 μm I.D. × 250 mm, 1.9 μm, Shanghai Omicsolution Co., Ltd.) with a gradient elution of acetonitrile (ACN) as follows: 3–15% ACN from 0-70 min, 15–30% ACN from 70-100 min, 30–40% ACN from 100-110 min, and 35–85% ACN from 105-120 min, at a flow rate of 300 nL/min. The source voltage was set to 2.0 kV, and the ion transfer tube was maintained at 320 °C. MS1 spectra were acquired in the Orbitrap over an m/z range of 400–1,300 at a resolution of 120,000, with the RF lens at 60% and an AGC target of 4 × 10^5^. The background ion (C_2_H_6_SiO)_7_H^+^ (m/z = 519.1388) served as the lock mass for internal calibration. Due to the complexity of the proteome, MS2 spectra were acquired in data-independent acquisition (DIA) mode, with all precursor ions sequentially fragmented in 10 m/z windows at 30% HCD collision energy, using an automatic m/z range, a resolution of 30,000, and an AGC target of 8 × 10^5^.

### Database search and data analysis

Spectronaut 18.1 were employed for qualitative and quantitative analysis of data acquired in DIA mode. Raw files were searched against the Uniprot-Verticillium dahliae (Verticillium wilt) proteome database (Proteome ID: UP000288725, 2024-04-07). Trypsin was designated as the digestion enzyme, allowing for up to two missed cleavages. The database search accounted for common variable modifications, including methionine oxidation and N-terminal acetylation, whereas cysteine carbamidomethylation was set as a fixed modification. Quality control was implemented at the levels of PSMs, peptides, and proteins using a 1% false discovery rate (FDR) based on a target-decoy approach. Quantification was performed using fragment-ion area with interference correction enabled and protein abundance calculated from the average of its top 1–3 peptides.

The data from mass spectrometry was filtered to remove invalid entries. Missing values in the input and pulldown groups were set to one-fifth of the minimum value in the same dataset; if a strain had no data in the pulldown group, the value was set to one-fifth of the minimum from the other strain. The abundance of each protein from mass spectrometry was normalized to the average value of all protein abundances in this group. S-acylated proteins were defined as those with Pulldown + /Pulldown− > 5 (designated as Pos/Neg in datasets). Log_2_FC was calculated as the ratio between (PosWT/NegWT)/InputWT and (PosΔ*VdERF2*/NegΔ*VdERF2*)/InputΔ*VdERF2*, where Log_2_FC ≥ 1 indicated up-regulated proteins, Log_2_FC ≤ 1 indicated down-regulated proteins, and 0 < Log_2_FC < 1 were regarded as non-differential proteins (*p* < 0.05). PCA, volcano plots, and clustering were performed using Wei Sheng Xin (https://www.bioinformatics.com.cn/). GO enrichment and KEGG pathway analyses were conducted for up-regulatd proteins using GENE DENOVO (https://www.omicshare.com/tools/) and KOBAS (http://bioinfo.org/kobas/); figures were generated using GENE DENOVO (https://www.omicshare.com/tools/).

### Quantitative RT-PCR and quantitative PCR analysis

Total RNA was extracted using the HiPure Plant RNA Mini Kit (Magen Biotech, China) and used as the template for reverse transcription with the HiScript IV RT SuperMix (Vazyme Biotech, China). The quantitative PCR assays were performed using the Universal TaqMan multiplex qPCR master mix (Yeasen Biotechnology Co., Ltd, China). The *V. dahliae VdEF-1α* (elongation factor-1*α*) gene and the cotton *GhSSU* (small subunit ribosomal rRNA) gene were employed as reference genes for normalization. Quantitative PCR was conducted in a Bio-Rad CFX 96 system (C1000 Thermal Cycler). Relative product levels across different samples were determined using the 2^-ΔΔCT^ method.

### Co-immunoprecipitation assay

VdERF2-Flag_3_ was co-overexpressed with either VdHIUH-GFP or GFP in protoplasts. Proteins were extracted in 750 μL of lysis buffer (20 mM Tris-HCl pH7.4, 100 mM NaCl, 10% glycerol, 0.5% NP-40, and 1× protease inhibitor cocktail) for 20 min at 4 °C. Following centrifugation at 13,000 *g* for 10 min at 4 °C, 90 μL of the supernatant was retained as the input sample. The remaining lysate was incubated with Anti-GFP Magarose Beads (Smart-Lifesciences, SM038001) for 2 h at 4 °C. The beads were then rinsed three times with wash buffer (20 mM Tris-HCl pH 7.4, 100 mM NaCl, 10% glycerol, and 0.1% NP40). Finally, proteins were eluted by adding 80 μL of 2× protein sample buffer supplemented with 2.5% β-mercaptoethanol, followed by SDS-PAGE and immunoblotting using anti-GFP antibody (TransGen Biotech, HT801–01) and anti-Flag antibody (Biodragon Biotech, B1020).

### Quantitative analysis of uric acid and H_2_O_2_

To assess the roles of VdERF2 and VdHIUH on purine catabolism in *V. dahliae*, the contents of uric acid and H_2_O_2_ were quantified in the knockout and complementation strains. All strains were cultured on PDA plates for 3 days, followed by harvesting of mycelia. Uric acid levels in each strain were then measured according to the protocol provided with the uric acid content assay kit (Solarbio, BC1360) or H_2_O_2_ content assay kit (Solarbio, BC3595). The H_2_O_2_ content in cotton tissues was quantified using the H_2_O_2_ content assay kit (Solarbio, BC3590); for extraction, 2-cm basal stem segments were harvested from cotton plants at 48 h post-inoculation.

### HIU hydrolase activity measurement

The HIU hydrolase activity was assayed based on previously described [26]. The gene encoding uricase was cloned into the *pET28a* vector, then transformed into *E. coli* BL21, and the recombinant His_6_-tagged uricase was obtained through Ni-NTA agarose (QIAGEN) purification. 500 μL assay mixtures contained 0.1 µM recombinant uricase in 50 mM potassium phosphate buffer (pH 7.8). Upon addition of 50 μM uric acid (Sigma) to the mixtures, the uricase-mediated reaction nearly reached completion in 60 seconds, as determined by monitoring the absorbance at 292 nm. VdHIUH(WT)-GFP or VdHIUH(C9S)-GFP proteins expressed in the WT or Δ*VdERF2* strains of *V. dahliae* were purified using anti-GFP agarose. Subsequently, 20 µL of equal amounts (approximately 0.1 µM each) of the GFP-fused VdHIUH proteins were added separately to the reaction mixtures. 5-HIU was generated by the reaction catalyzed by urate oxidase and the disappearance of HIU was monitored at 312 nm. The absorbance value at the 300th second of the reaction was recorded, and the relative activity of VdHIUH was calculated accordingly.

### Molecular dynamics simulations

The initial VdHIUH model was constructed using homology modeling in SWISS-MODEL, with the available crystal structure serving as the template. Three independent simulation systems were subsequently prepared using the CHARMM-GUI pipeline for implementation in GROMACS 2024.6, employing the CHARMM36m all-atom force field [27]. All analyses were performed using the GROMACS toolkit integrated within the GROMACS software suite.

## Supporting information

S1 Fig Bioinformatic analysis of the VdPAT family members. **(A)** Schematic representation of the protein structure of the VdPATs family members. Transmembrane domains are highlighted in blue, and cysteine-rich regions containing the DHHC motif are shown in orange. **(B)** Phylogenetic analysis of the amino acid sequences of VdPATs family members from *V. dahliae* and their homologs from other plant-pathogenic fungi was performed using MEGA 7 with the maximum likelihood method, based on 1,000 bootstrap replications. Ss, *Sclerotinia sclerotiorum*; Bc, *Botrytis cinerea*; Mo, *Magnaporthe oryzae*; Uv, *Ustilaginoidea virens*; Fg, *Fusarium graminearum*; Vd, *Verticillium dahliae*.
(PDF)

S2 Fig Sequence alignment of VdPAT homologs across plant pathogenic fungi. Multiple sequence alignment of the DHHC-CRD regions of VdPAT homolog proteins from the indicated fungal species were performed using DNAMAN. The DHHC/DHYC motifs are highlighted with red lines. Ss, *Sclerotinia sclerotiorum*; Bc, *Botrytis cinerea*; Mo, *Magnaporthe oryzae*; Uv, *Ustilaginoidea virens*; Fg, *Fusarium graminearum*; Vd, *Verticillium dahliae*.
(PDF)

S3 Fig Characterization of knockout mutant strains for *VdPAT*s and *VdHIUH.* **(A)** Schematic representation of homologous recombination for gene knockout in *V. dahliae*. The primer pairs targeting the franking genomic regions (UU-F and DD-R) and the *HYG* gene (HYG-F and HYG-R) are indicated. **(B, C)** Verification of knockout mutant strains by genomic PCR. Two independent strains for each gene, including *VdPAT*s **(B)** and *VdHIUH* **(C)**, were confirmed. The WT strain and water served as negative controls. M: molecular weight marker; U: PCR product amplified using UU-F and HYG-R; D: PCR product amplified using DD-R and HYG-F.
(PDF)

S4 Fig Functional analysis of VdPAT family members in the virulence of *V. dahliae.* Representative disease symptoms observed in cotton plants infected with the indicated *V. dahliae* strains at 14 dpi are shown in **(A)**. Relative fungal biomass in infected cotton plants **(B)** was measured by quantitative PCR, using *GhSSU* as the internal reference gene and *VdEF-1α* as the target gene. Data are expressed as means ± SD from three replicates. Statistical significance was assessed by one-way ANOVA followed by Tukey’s multiple comparison test. Different letters indicate significant differences (*p* < 0.05). All data in this figure are representative of three biologically independent experiments.
(PDF)

S5 Fig PCA analysis of quantitative proteomics and GO annotation of VdERF2-dependent S-acylated proteins. **(A)** PCA analysis of quantitative proteomics data derived from three biologically independent replicate samples of the WT and Δ*VdERF2* strains. **(B, C)** GO enrichment analysis of VdERF2-dependent S-acylated proteins. The top15 significantly enriched GO terms in cellular component **(B)** and biological process **(C)** categories are shown, based on proteins exhibiting increased S-acylation ratios (WT/Δ*VdERF2*).
(PDF)

S6 Fig Detection of the interaction between VdERF2 and VdHIUH. Co-immunoprecipitation (Co-IP) was employed to assess the interaction between VdERF2-Flag and VdHIUH-GFP. Proteins were immunoprecipitated using anti-GFP beads; both input and IP samples were subjected to SDS-PAGE followed by immunoblotting with anti-GFP and anti-Flag antibodies. Representative immunoblots from three biologically independent experiments are presented.
(PDF)

S7 Fig Bioinformatic analysis of VdHIUH homologs across plant pathogenic fungi. **(A)** Multiple sequence alignment of HIUH proteins from indicated plant-pathogenic fungi using DNAMAN, with asterisks (*) denoting the conserved cysteine residue. **(B)** Phylogenetic analysis of the amino acid sequences of VdHIUH from *V. dahliae* and its homologs from other plant-pathogenic fungi was conducted using MEGA 7 with the maximum likelihood method, based on 1,000 bootstrap replicates. Ss, *Sclerotinia sclerotiorum*; Bc, *Botrytis cinerea*; Mo, *Magnaporthe oryzae*; Uv, *Ustilaginoidea virens*; Fg, *Fusarium graminearum*; Vd, *Verticillium dahliae*.
(PDF)

S8 Fig Detection of the effect of S-acylation on the activity, stability, and subcellular localization of VdHIUH. **(A, B)** Analysis of the impact of VdERF2-dependent S-acylation on the protein stability of VdHIUH. The transcript levels of *VdHIUH* in different strains, quantified by quantitative RT-PCR, are presented in **(A)**. Protein levels of VdHIUH-GFP, detected by anti-GFP immunoblotting, are shown in **(B)**. **(C)** Analysis of the effect of VdERF2-mediated S-acylation on the subcellular localization of VdHIUH. Representative microscopic images are displayed, with GFP signal in green and brightfield background in gray. Bar = 1 µm. **(D, E)** The Schematic representation of the method used to measure VdHIUH enzyme activity. The *V. dahliae* strains expressing GFP-tagged VdHIUH were cultured in CM medium for 3 days. Total proteins were subsequently extracted and purified using GFP-trap agarose. Recombinant uricase was firstly incubated with uric acid to generate 5-HIU. Purified VdHIUH-GFP proteins were then added to the reaction mixture, and the change in absorbance was monitored. A summary image of the assay procedure illustrated in **(D)** was generated by the authors using Figdraw (www.figdraw.com; copyright code:YTYRYaad5b). The levels of GFP-tagged proteins purified from the indicated strains, as determined by anti-GFP immunoblotting, are shown in **(E)**. Quantitative data are presented as means ± SD from three replicates. Statistical significance was determined by one-way ANOVA followed by Tukey’s multiple comparison test. Different letters indicate significant differences (*p* < 0.05). All results are representative of three biologically independent experiments.
(PDF)

S9 Fig Assessment of the phenotypic effects of VdHIUH overexpression in Δ*VdERF2.* **(A, B)** Colony growth phenotypes of strains overexpressing VdHIUH in the WT or Δ*VdERF2* genetic backgrounds on PDA plates. Representative photographs taken 7 days post-incubation are shown in **(A)**, and corresponding colony diameters are quantified in **(B)**. **(C, D)** Impact of VdHIUH overexpression on the virulence of WT or Δ*VdERF2* strains. Representative disease symptoms observed in cotton plants inoculated with the indicated *V. dahliae* strains at 14 dpi are presented in **(C)**. Relative fungal biomass in infected cotton tissues **(D)** was determined by quantitative PCR, using *GhSSU* as the endogenous reference gene and *VdEF-1α* as the target gene. Data represent means ± SD from three replicates. Statistical significance was assessed by one-way ANOVA followed by Tukey’s multiple comparison test; different letters denote significant differences (*p* < 0.05). All data shown in this figure are representative of three independent biological experiments.
(PDF)

S10 Fig Analysis of the effects of allantoin treatment on the virulence of the Δ*VdERF2* and Δ*VdHIUH* mutants. **(A, B)** WT, Δ*VdERF2*, and Δ*VdHIUH* strains of *V. dahliae* were cultured in PDB medium supplemented with either 0.4 mM allantoin or DMSO as a solvent control for five days. Conidia were harvested and used to inoculate cotton seedlings. Prior to inoculation, cotton roots were pretreated with or without 0.4 mM allantoin. Representative disease symptoms observed in cotton plants infected with the indicated *V. dahliae* strains at 21 dpi are shown in **(A)**. Relative fungal biomass in infected cotton tissues **(B)** was quantified by quantitative PCR, using *GhSSU* as the endogenous reference gene and *VdEF-1α* as the target gene. **(C)** Effect of allantoin pretreatment on H_2_O_2_ accumulation in cotton roots infected with the indicated *V. dahliae* strains. Cotton roots were pretreated with 0.4 mM allantoin prior to inoculation with WT, Δ*VdERF2*, or Δ*VdHIUH* strains, and H_2_O_2_ levels in root tissues were measured at 48 h post-inoculation. Quantitative data represent means ± SD from three replicates. Statistical significance was determined by Student’s *t*-test (*p < 0.05, ***p < 0.001, ****p < 0.0001, ns. *p* > 0.05). All data presented in this figure are representative of three independent biological experiments.
(PDF)

S1 Dataset The information of identified input and S-acylated proteins from proteomics. (XLSX)

S2 Dataset Bioinformatics analysis of input and S-acylated proteins derived from proteomic data. (XLSX)

S3 Dataset Primers used in this study. (XLSX)

S1 Raw Gel The raw gel images used for protein and DNA detection in the manuscript. (PDF)
