# Supplementary material for: Comparative S-acyl-proteomics reveals post-translational modulation of fungal purine metabolism for Verticillium wilt
Source: PLoS Pathog. 2026 Oct 26;22(10):e1014730. doi: 10.1371/journal.ppat.1014730 (PMC13722310; doi:10.1371/journal.ppat.1014730)
Supplement: Supplementary file 1 [file ppat.1014730.s001.pdf]

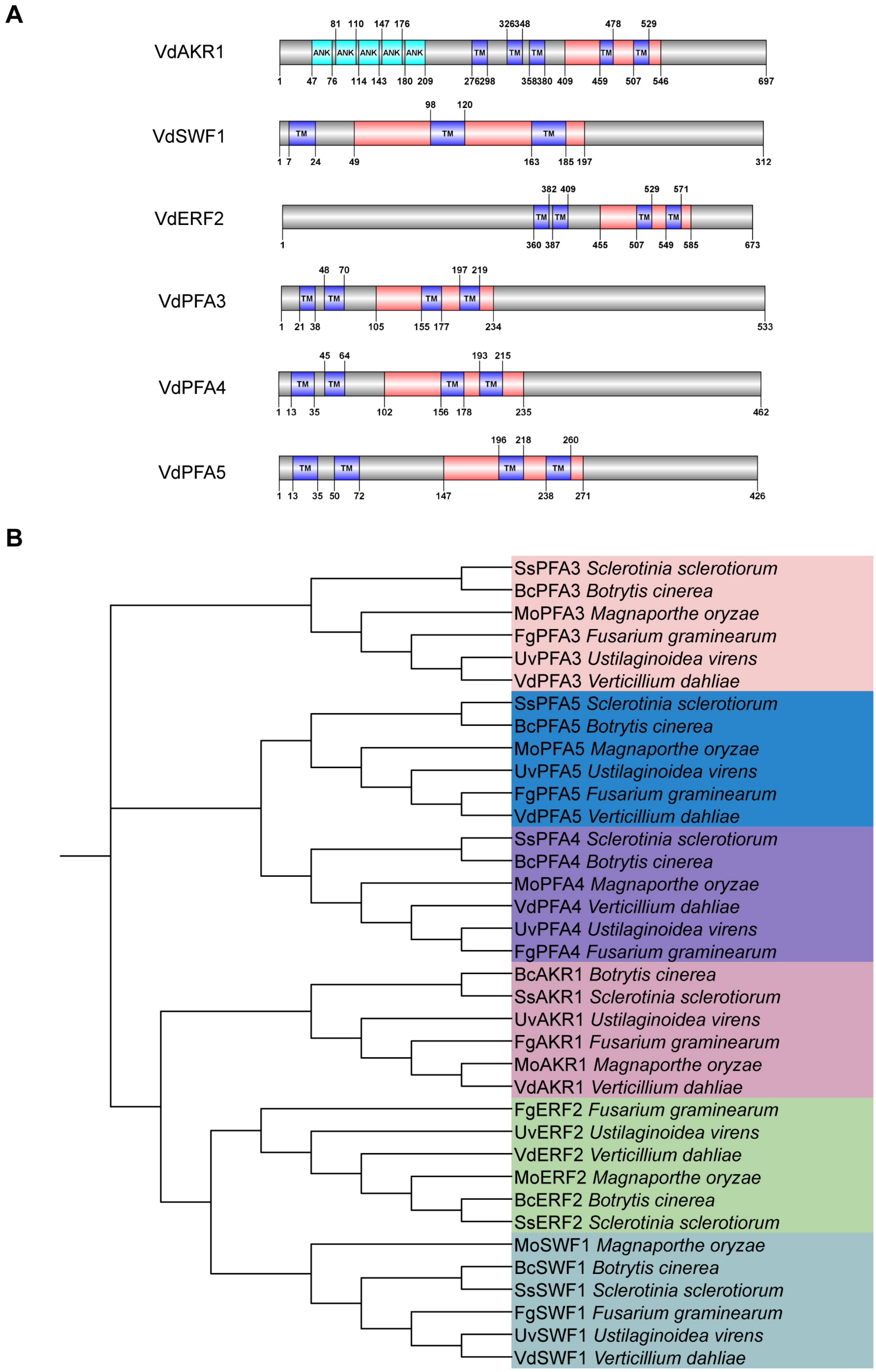
A
VdAKR1
ANK
TM
81 110 147 176 326348 478 529
1 47 76 114 143 180 209 276298 358380 409 459 507 546 697
VdSWF1
98 120
1 7 24 49 163 185 197 312
VdERF2
382 409 529 571
1 360 387 455 507 549 585 673
VdPFA3
48 70 197 219
1 21 38 105 155 177 234 533
VdPFA4
45 64 193 215
1 13 35 102 156 178 235 462
VdPFA5
196 218 260
1 13 35 50 72 147 238 271 426
B
SsPFA3 Sclerotinia sclerotiorum
BcPFA3 Botrytis cinerea
MoPFA3 Magnaporthe oryzae
FgPFA3 Fusarium graminearum
UvPFA3 Ustilaginoidea virens
VdPFA3 Verticillium dahliae
SsPFA5 Sclerotinia sclerotiorum
BcPFA5 Botrytis cinerea
MoPFA5 Magnaporthe oryzae
UvPFA5 Ustilaginoidea virens
FgPFA5 Fusarium graminearum
VdPFA5 Verticillium dahliae
SsPFA4 Sclerotinia sclerotiorum
BcPFA4 Botrytis cinerea
MoPFA4 Magnaporthe oryzae
VdPFA4 Verticillium dahliae
UvPFA4 Ustilaginoidea virens
FgPFA4 Fusarium graminearum
BcAKR1 Botrytis cinerea
SsAKR1 Sclerotinia sclerotiorum
UvAKR1 Ustilaginoidea virens
FgAKR1 Fusarium graminearum
MoAKR1 Magnaporthe oryzae
VdAKR1 Verticillium dahliae
FgERF2 Fusarium graminearum
UvERF2 Ustilaginoidea virens
VdERF2 Verticillium dahliae
MoERF2 Magnaporthe oryzae
BcERF2 Botrytis cinerea
SsERF2 Sclerotinia sclerotiorum
MoSWF1 Magnaporthe oryzae
BcSWF1 Botrytis cinerea
SsSWF1 Sclerotinia sclerotiorum
FgSWF1 Fusarium graminearum
UvSWF1 Ustilaginoidea virens
VdSWF1 Verticillium dahliae

Fig S1
