# Supplementary material for: Comparative S-acyl-proteomics reveals post-translational modulation of fungal purine metabolism for Verticillium wilt
Source: PLoS Pathog. 2026 Oct 26;22(10):e1014730. doi: 10.1371/journal.ppat.1014730 (PMC13722310; doi:10.1371/journal.ppat.1014730)
Supplement: Supplementary file 2 [file ppat.1014730.s002.pdf]

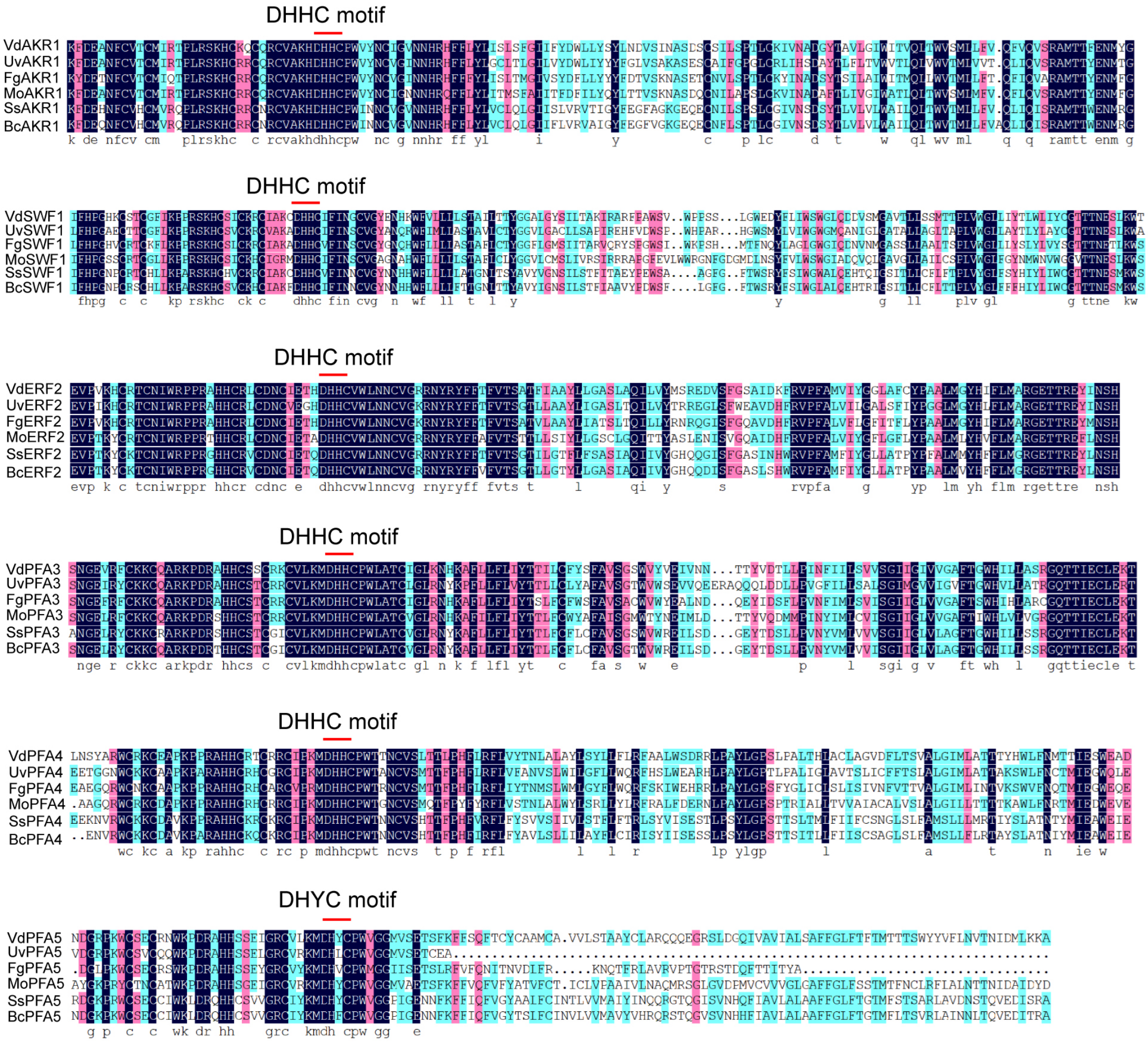
DHHC motif
VdAKR1
UvAKR1
FgAKR1
MoAKR1
SsAKR1
BcAKR1
DHHC motif
VdSWF1
UvSWF1
FgSWF1
MoSWF1
SsSWF1
BcSWF1
DHHC motif
VdERF2
UvERF2
FgERF2
MoERF2
SsERF2
BcERF2
DHHC motif
VdPFA3
UvPFA3
FgPFA3
MoPFA3
SsPFA3
BcPFA3
DHHC motif
VdPFA4
UvPFA4
FgPFA4
MoPFA4
SsPFA4
BcPFA4
DHYC motif
VdPFA5
UvPFA5
FgPFA5
MoPFA5
SsPFA5
BcPFA5

Fig S2
