# Supplementary material for: Comparative S-acyl-proteomics reveals post-translational modulation of fungal purine metabolism for Verticillium wilt
Source: PLoS Pathog. 2026 Oct 26;22(10):e1014730. doi: 10.1371/journal.ppat.1014730 (PMC13722310; doi:10.1371/journal.ppat.1014730)
Supplement: Supplementary file 3 [file ppat.1014730.s003.pdf]

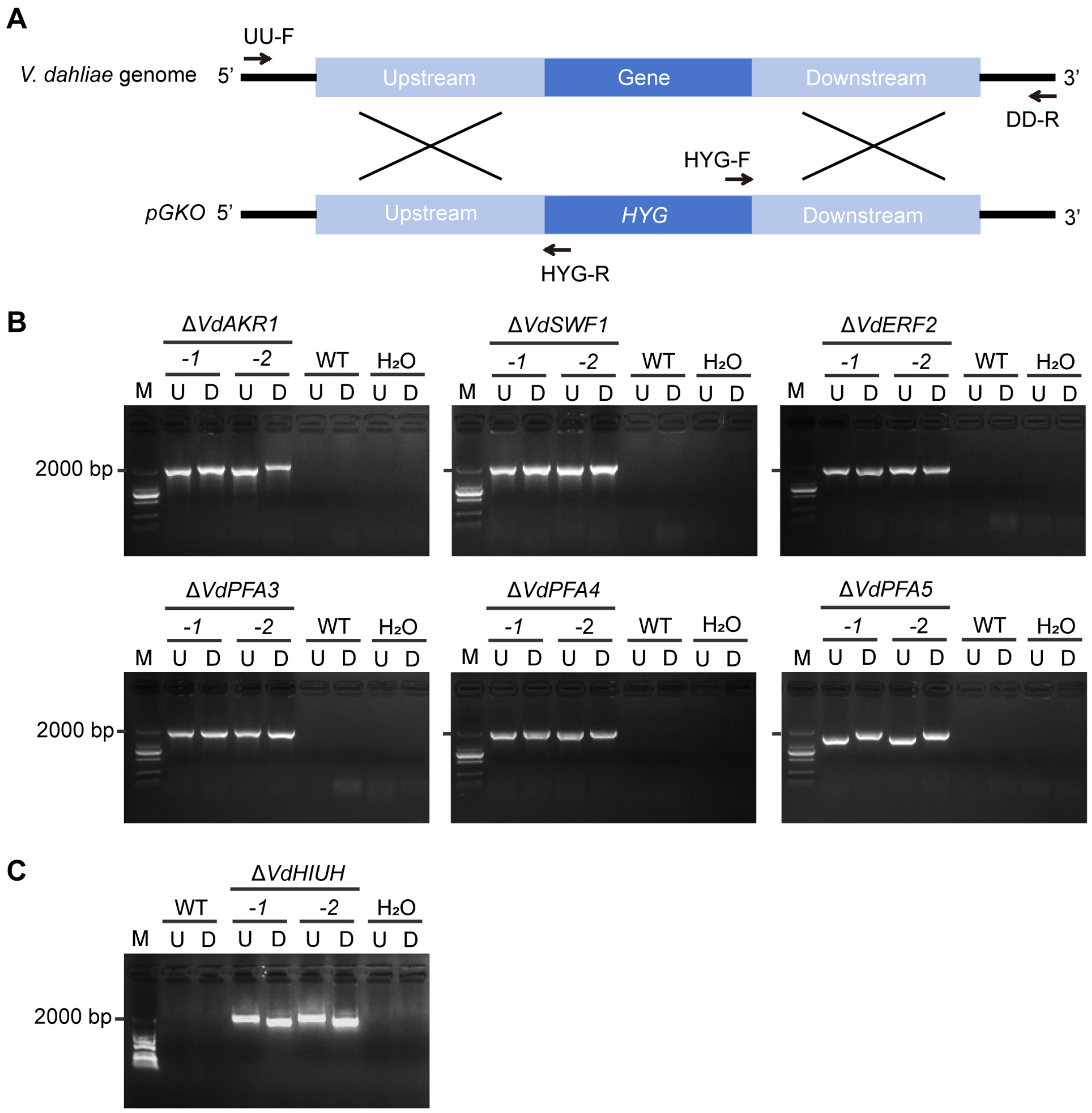
A
UU-F
V. dahliae genome 5'
Upstream
Gene
Downstream
3'
DD-R
HYG-F
pGKO 5'
Upstream
HYG
Downstream
3'
HYG-R
B
ΔVdAKR1
-1
-2
WT
H2O
M U D U D U D U D
2000 bp
ΔVdSWF1
ΔVdERF2
ΔVdPFA3
ΔVdPFA4
ΔVdPFA5
C
ΔVdHIUH
WT
-1
-2
H2O
M U D U D U D U D
2000 bp

Fig S3
