# Supplementary material for: Comparative S-acyl-proteomics reveals post-translational modulation of fungal purine metabolism for Verticillium wilt
Source: PLoS Pathog. 2026 Oct 26;22(10):e1014730. doi: 10.1371/journal.ppat.1014730 (PMC13722310; doi:10.1371/journal.ppat.1014730)
Supplement: Supplementary file 4 [file ppat.1014730.s004.pdf]

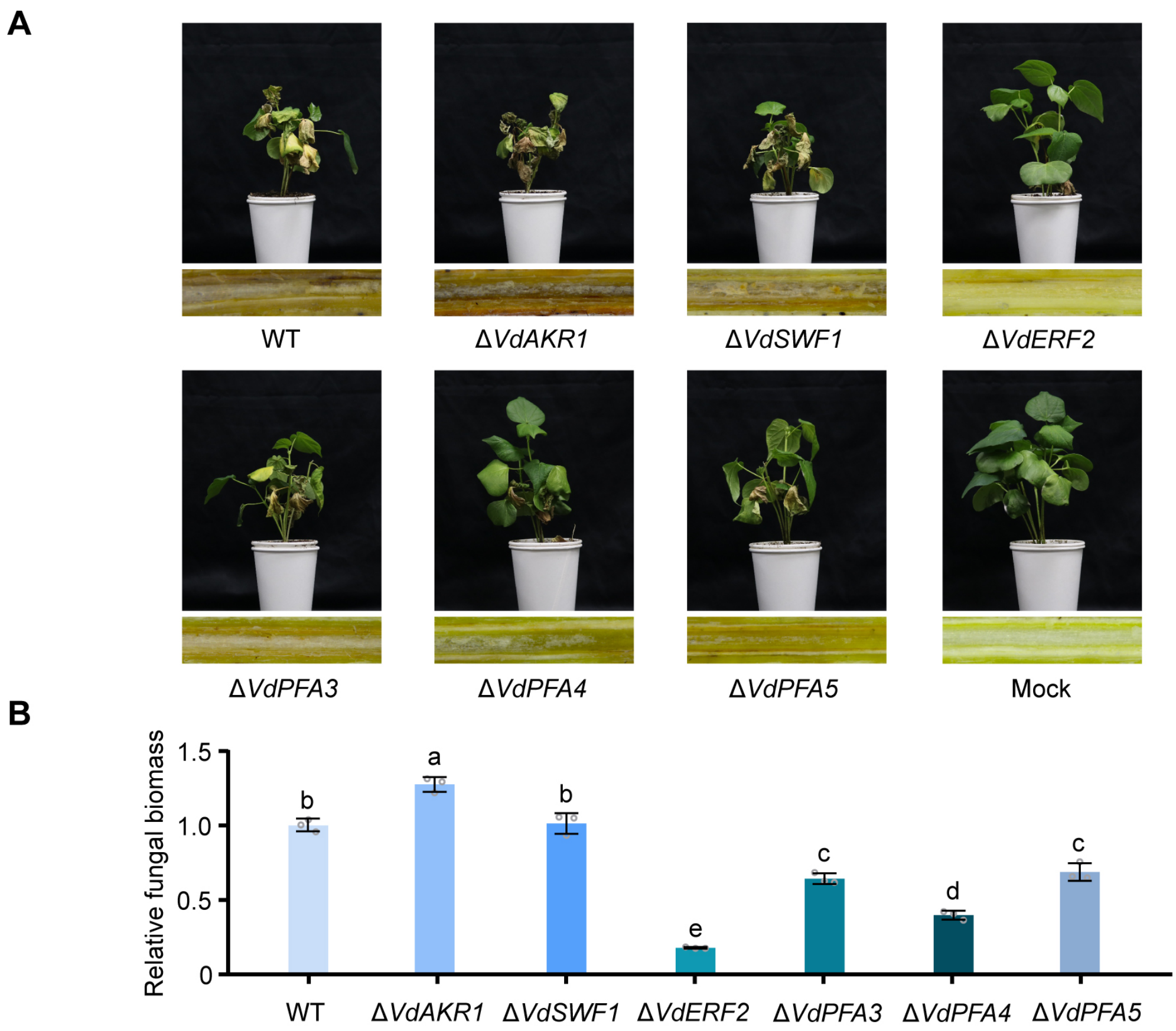
A
WT
ΔVdAKR1
ΔVdSWF1
ΔVdERF2
ΔVdPFA3
ΔVdPFA4
ΔVdPFA5
Mock
B
Relative fungal biomass
1.5
1.0
0.5
0
b
a
b
e
c
d
c
WT
ΔVdAKR1
ΔVdSWF1
ΔVdERF2
ΔVdPFA3
ΔVdPFA4
ΔVdPFA5

Fig S4
