# Supplementary material for: Comparative S-acyl-proteomics reveals post-translational modulation of fungal purine metabolism for Verticillium wilt
Source: PLoS Pathog. 2026 Oct 26;22(10):e1014730. doi: 10.1371/journal.ppat.1014730 (PMC13722310; doi:10.1371/journal.ppat.1014730)
Supplement: Supplementary file 5 [file ppat.1014730.s005.pdf]

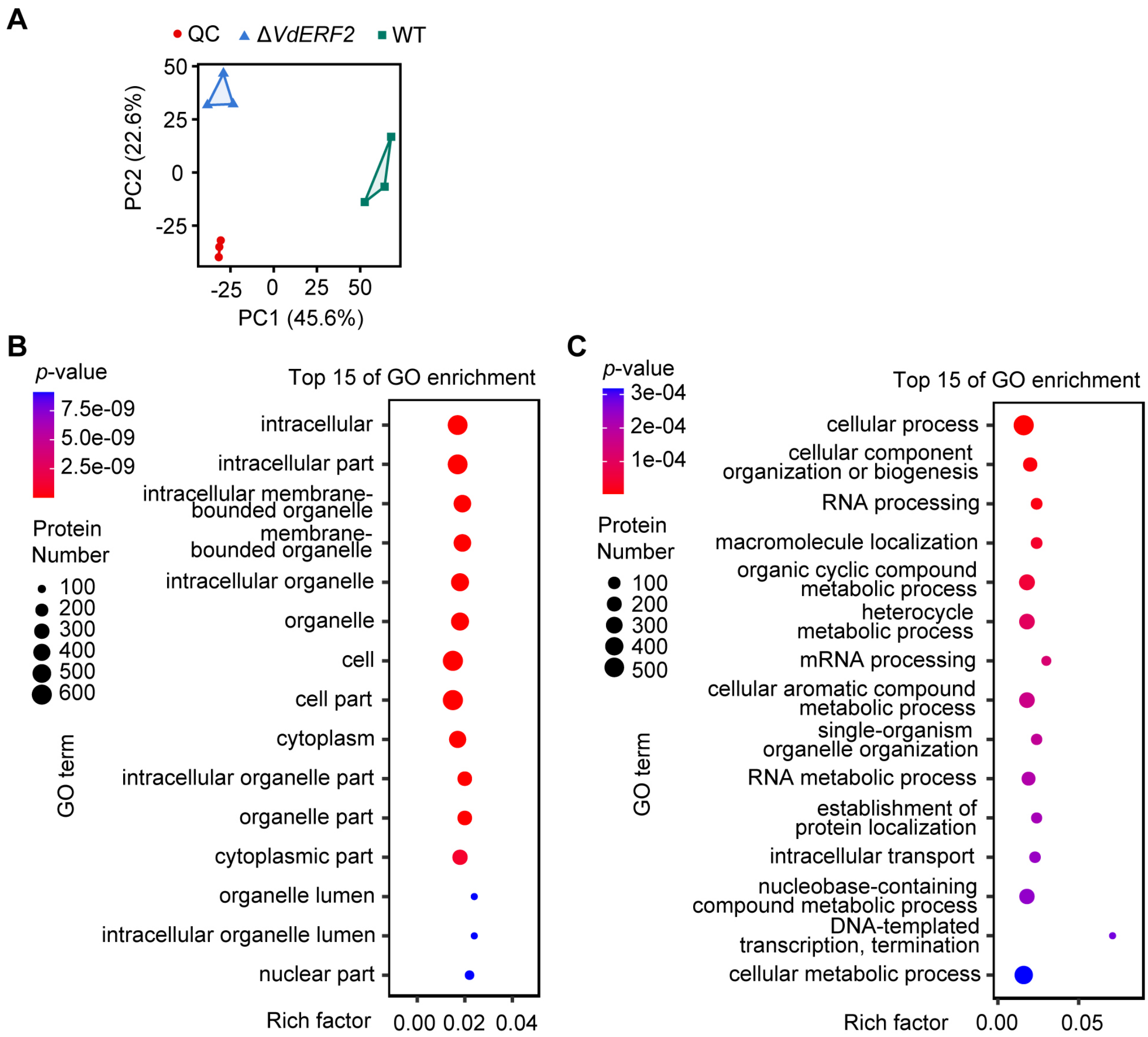
A
QC
ΔVdERF2
WT
PC2 (22.6%)
PC1 (45.6%)
B
p-value
7.5e-09
5.0e-09
2.5e-09
Protein Number
100
200
300
400
500
600
GO term
Top 15 of GO enrichment
intracellular
intracellular part
intracellular membrane-bounded organelle
membrane-bounded organelle
intracellular organelle
organelle
cell
cell part
cytoplasm
intracellular organelle part
organelle part
cytoplasmic part
organelle lumen
intracellular organelle lumen
nuclear part
Rich factor
C
p-value
3e-04
2e-04
1e-04
Protein Number
100
200
300
400
500
GO term
Top 15 of GO enrichment
cellular process
cellular component organization or biogenesis
RNA processing
macromolecule localization
organic cyclic compound metabolic process
heterocycle metabolic process
mRNA processing
cellular aromatic compound metabolic process
single-organism organelle organization
RNA metabolic process
establishment of protein localization
intracellular transport
nucleobase-containing compound metabolic process
DNA-templated transcription, termination
cellular metabolic process
Rich factor

Fig S5
