# Supplementary material for: Comparative S-acyl-proteomics reveals post-translational modulation of fungal purine metabolism for Verticillium wilt
Source: PLoS Pathog. 2026 Oct 26;22(10):e1014730. doi: 10.1371/journal.ppat.1014730 (PMC13722310; doi:10.1371/journal.ppat.1014730)
Supplement: Supplementary file 6 [file ppat.1014730.s006.pdf]

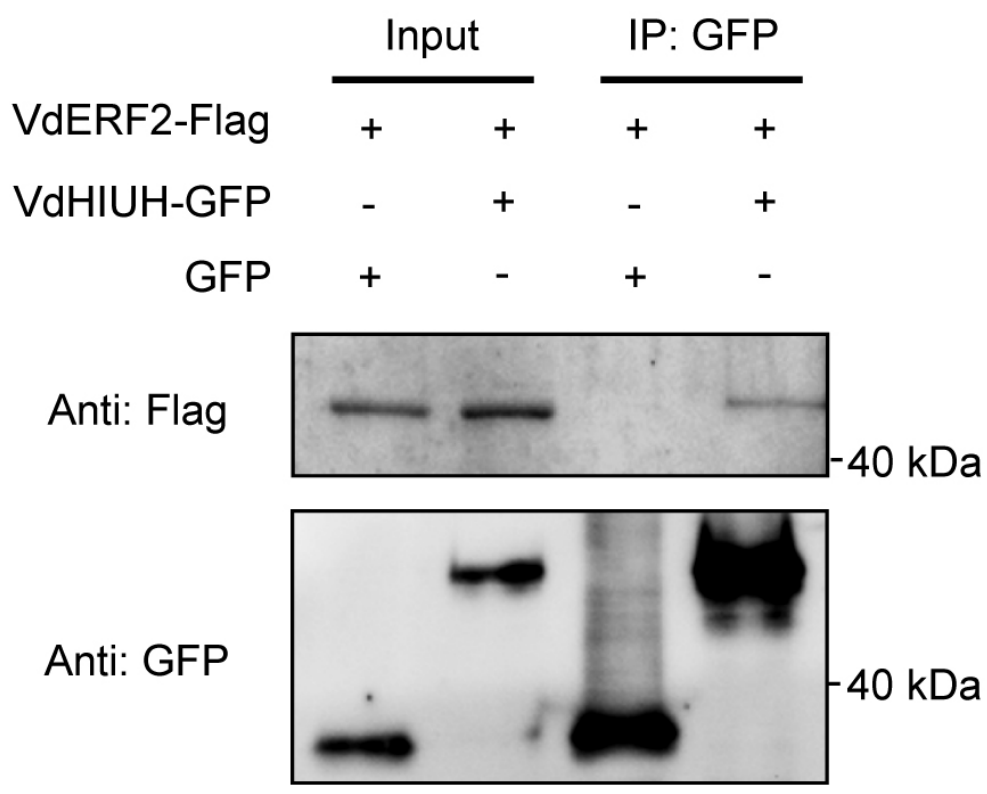
Input
IP: GFP
VdERF2-Flag + + + +
VdHIUH-GFP - + - +
GFP + - + -
Anti: Flag
40 kDa
Anti: GFP
40 kDa

Fig S6
