# Supplementary material for: Comparative S-acyl-proteomics reveals post-translational modulation of fungal purine metabolism for Verticillium wilt
Source: PLoS Pathog. 2026 Oct 26;22(10):e1014730. doi: 10.1371/journal.ppat.1014730 (PMC13722310; doi:10.1371/journal.ppat.1014730)
Supplement: Supplementary file 7 [file ppat.1014730.s007.pdf]

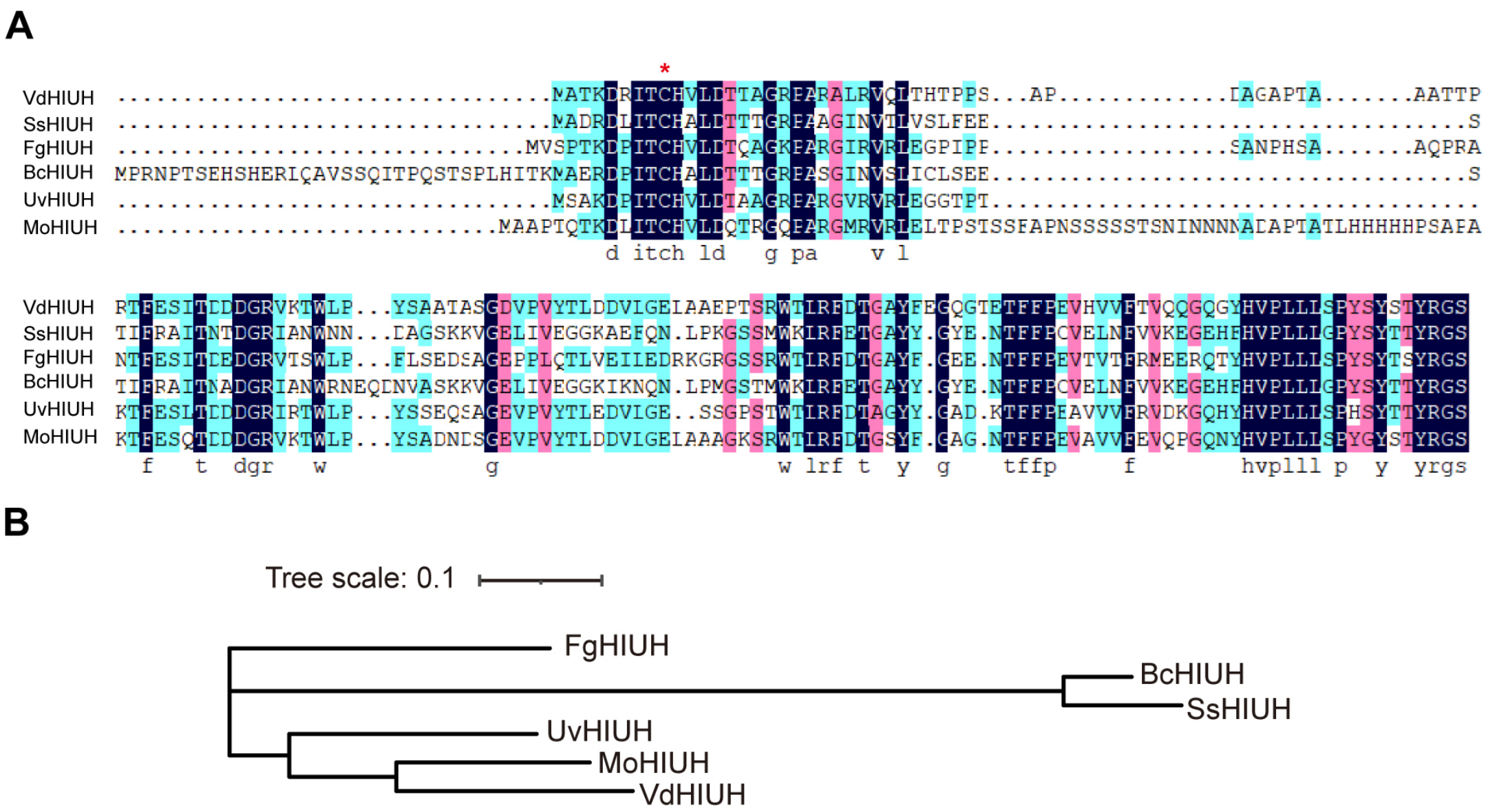
A
VdHIUH
SsHIUH
FgHIUH
BcHIUH
UvHIUH
MoHIUH
B
Tree scale: 0.1
FgHIUH
BcHIUH
SsHIUH
UvHIUH
MoHIUH
VdHIUH

Fig S7
