# Supplementary material for: Comparative S-acyl-proteomics reveals post-translational modulation of fungal purine metabolism for Verticillium wilt
Source: PLoS Pathog. 2026 Oct 26;22(10):e1014730. doi: 10.1371/journal.ppat.1014730 (PMC13722310; doi:10.1371/journal.ppat.1014730)
Supplement: Supplementary file 8 [file ppat.1014730.s008.pdf]

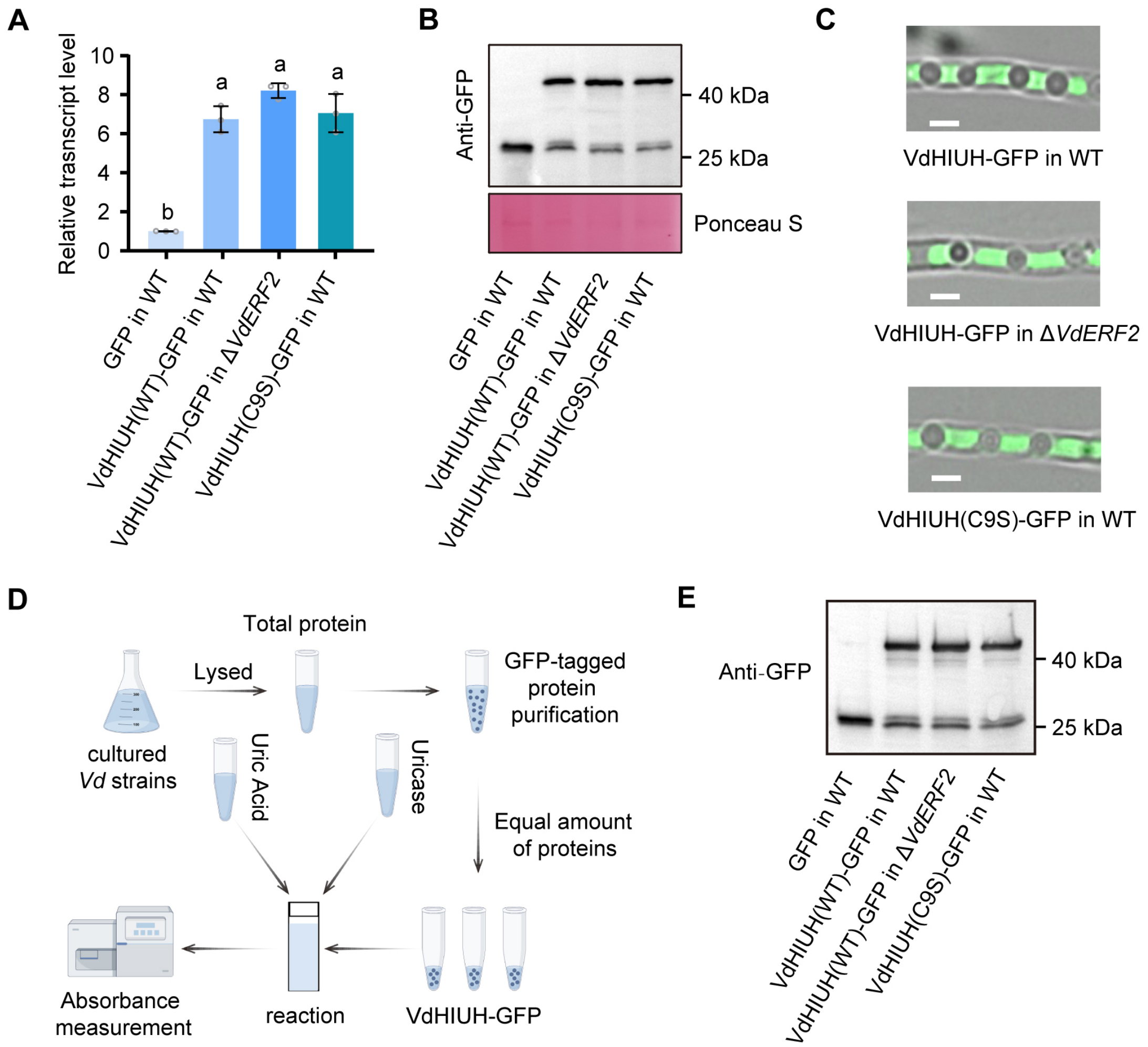
A
Relative trasnscript level
10
8
6
4
2
0
b
a
a
a
GFP in WT
VdHIUH(WT)-GFP in WT
VdHIUH(WT)-GFP in ΔVdERF2
VdHIUH(C9S)-GFP in WT
B
Anti-GFP
40 kDa
25 kDa
Ponceau S
GFP in WT
VdHIUH(WT)-GFP in WT
VdHIUH(WT)-GFP in ΔVdERF2
VdHIUH(C9S)-GFP in WT
C
VdHIUH-GFP in WT
VdHIUH-GFP in ΔVdERF2
VdHIUH(C9S)-GFP in WT
D
Total protein
Lysed
GFP-tagged protein purification
cultured Vd strains
Uric Acid
Uricase
Equal amount of proteins
Absorbance measurement
reaction
VdHIUH-GFP
E
Anti-GFP
40 kDa
25 kDa
GFP in WT
VdHIUH(WT)-GFP in WT
VdHIUH(WT)-GFP in ΔVdERF2
VdHIUH(C9S)-GFP in WT

Fig S8
