# Supplementary material for: Comparative S-acyl-proteomics reveals post-translational modulation of fungal purine metabolism for Verticillium wilt
Source: PLoS Pathog. 2026 Oct 26;22(10):e1014730. doi: 10.1371/journal.ppat.1014730 (PMC13722310; doi:10.1371/journal.ppat.1014730)
Supplement: Supplementary file 9 [file ppat.1014730.s009.pdf]

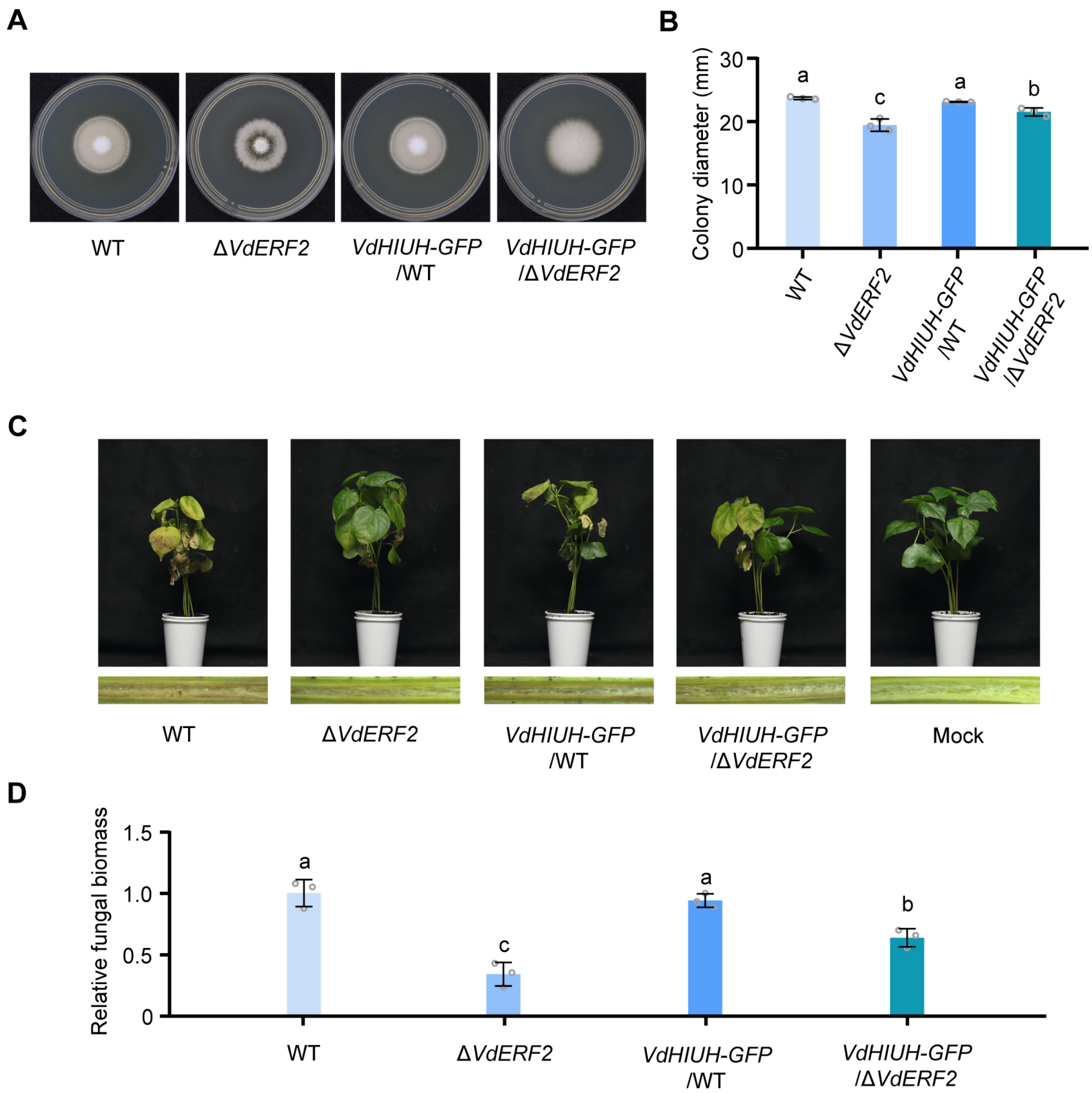
A
WT
ΔVdERF2
VdHIUH-GFP /WT
VdHIUH-GFP /ΔVdERF2
B
Colony diameter (mm)
30
20
10
0
a
c
a
b
WT
ΔVdERF2
VdHIUH-GFP /WT
VdHIUH-GFP /ΔVdERF2
C
WT
ΔVdERF2
VdHIUH-GFP /WT
VdHIUH-GFP /ΔVdERF2
Mock
D
Relative fungal biomass
1.5
1.0
0.5
0
a
c
a
b
WT
ΔVdERF2
VdHIUH-GFP /WT
VdHIUH-GFP /ΔVdERF2

Fig S9
