# Supplementary material for: Comparative S-acyl-proteomics reveals post-translational modulation of fungal purine metabolism for Verticillium wilt
Source: PLoS Pathog. 2026 Oct 26;22(10):e1014730. doi: 10.1371/journal.ppat.1014730 (PMC13722310; doi:10.1371/journal.ppat.1014730)
Supplement: Supplementary file 10 [file ppat.1014730.s010.pdf]

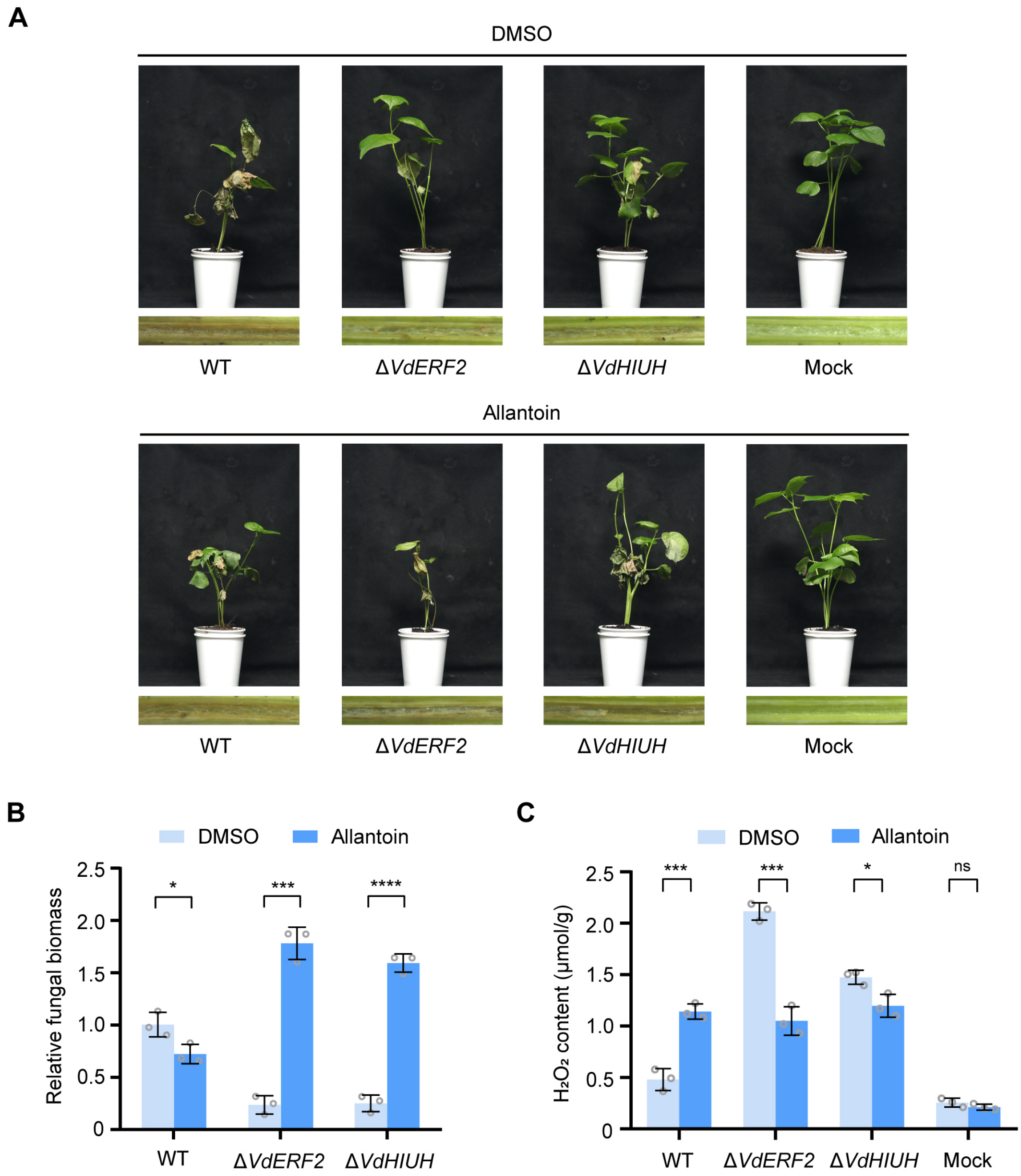
A
DMSO
WT
ΔVdERF2
ΔVdHIUH
Mock
Allantoin
WT
ΔVdERF2
ΔVdHIUH
Mock
B
DMSO
Allantoin
Relative fungal biomass
2.5
2.0
1.5
1.0
0.5
0
*
***
****
WT
ΔVdERF2
ΔVdHIUH
C
DMSO
Allantoin
H2O2 content (μmol/g)
2.5
2.0
1.5
1.0
0.5
0
***
***
*
ns
WT
ΔVdERF2
ΔVdHIUH
Mock

Fig S10
