# Supplementary material for: Comparative S-acyl-proteomics reveals post-translational modulation of fungal purine metabolism for Verticillium wilt
Source: PLoS Pathog. 2026 Oct 26;22(10):e1014730. doi: 10.1371/journal.ppat.1014730 (PMC13722310; doi:10.1371/journal.ppat.1014730)
Supplement: Supplementary file 11 [file ppat.1014730.s014.pdf]

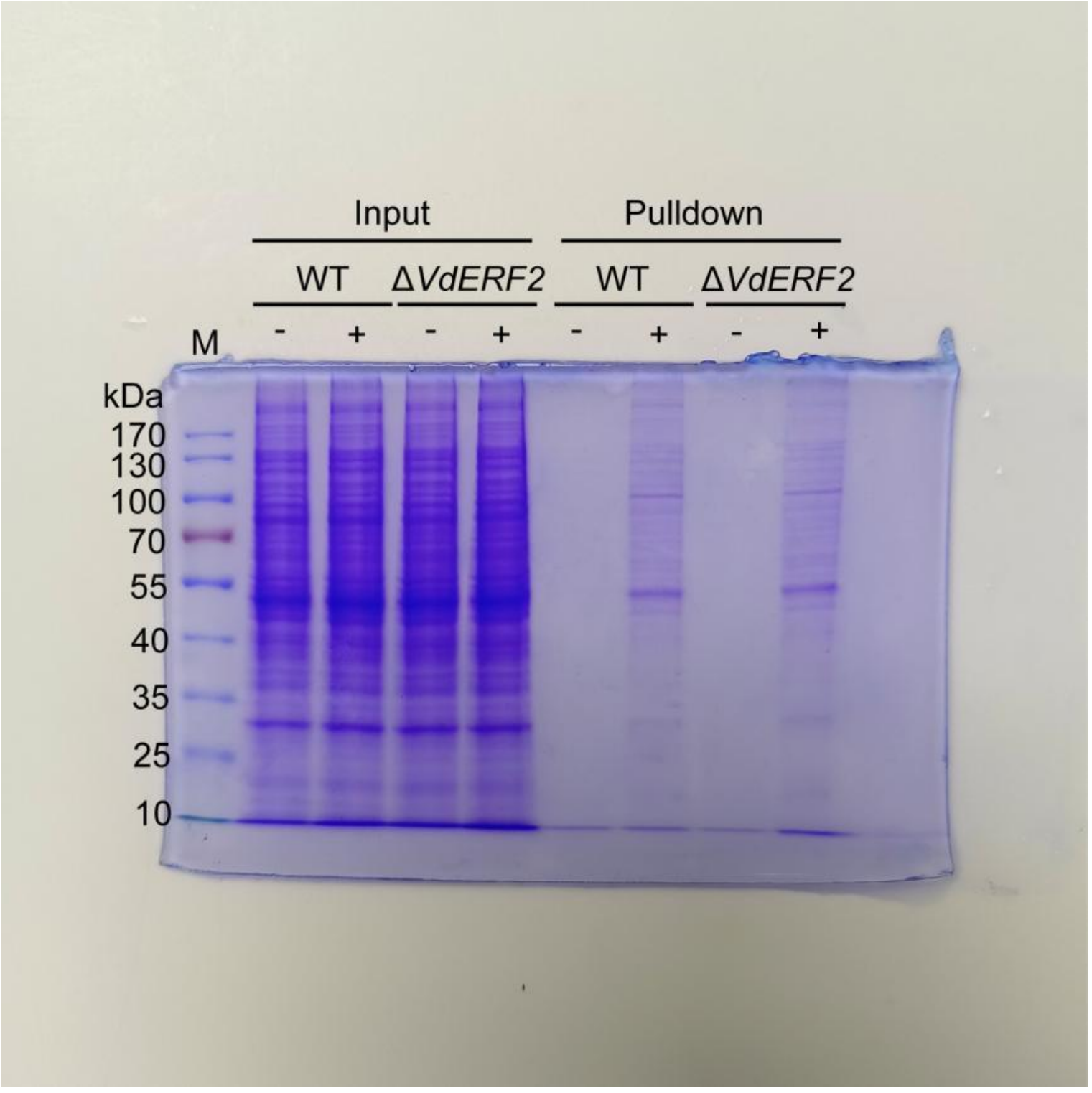

Figure 2B

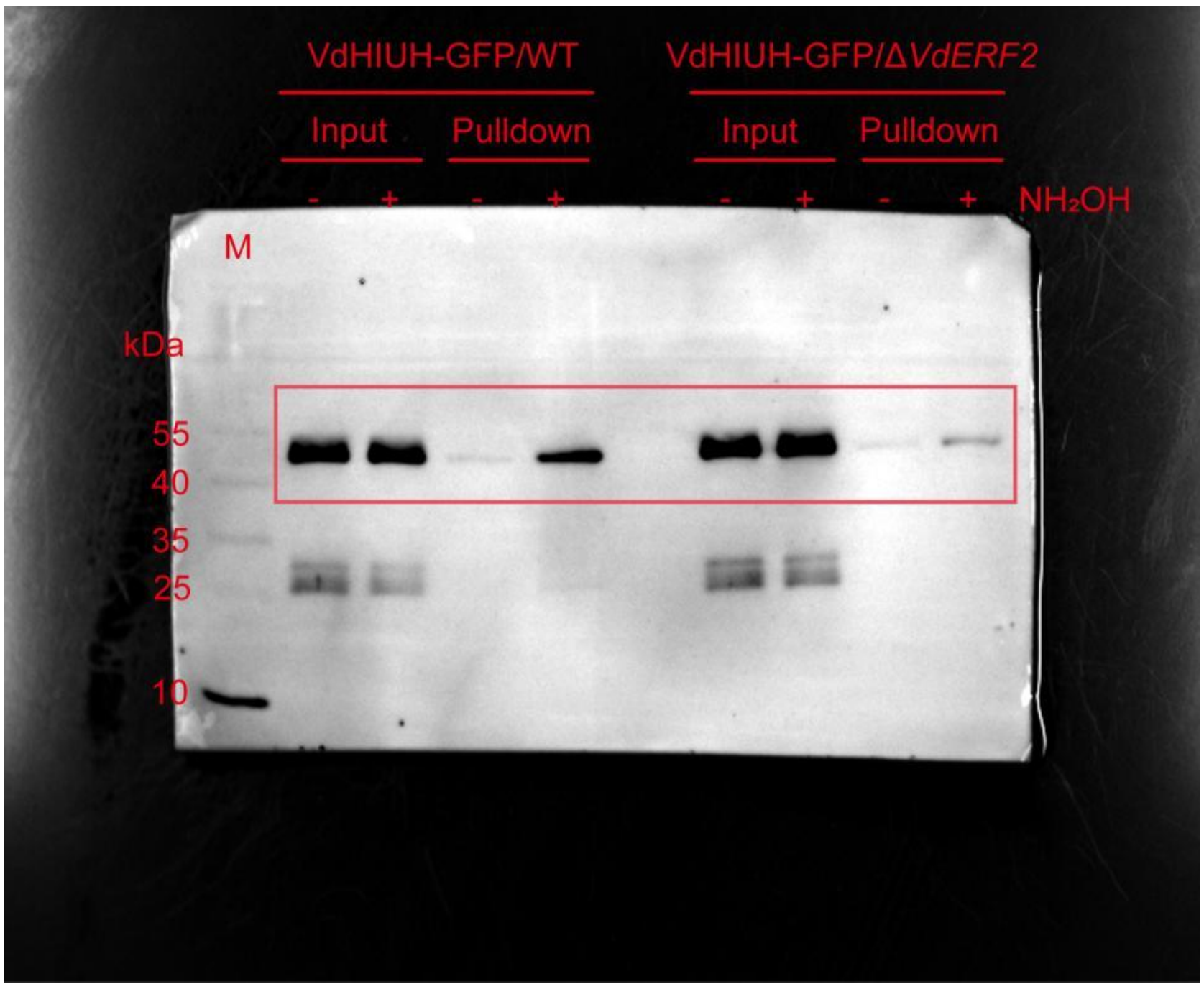

Figure 3A

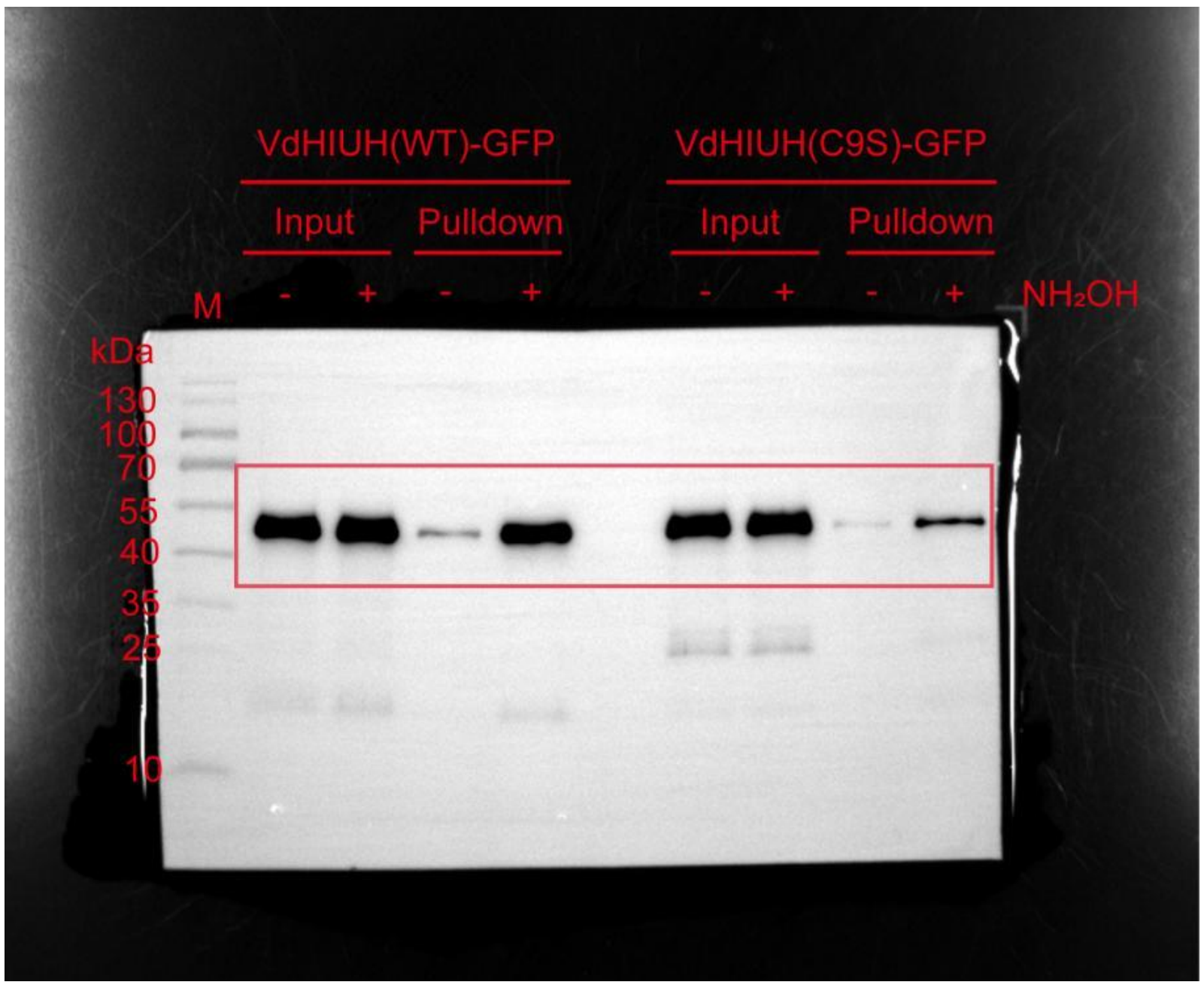

Figure 3B

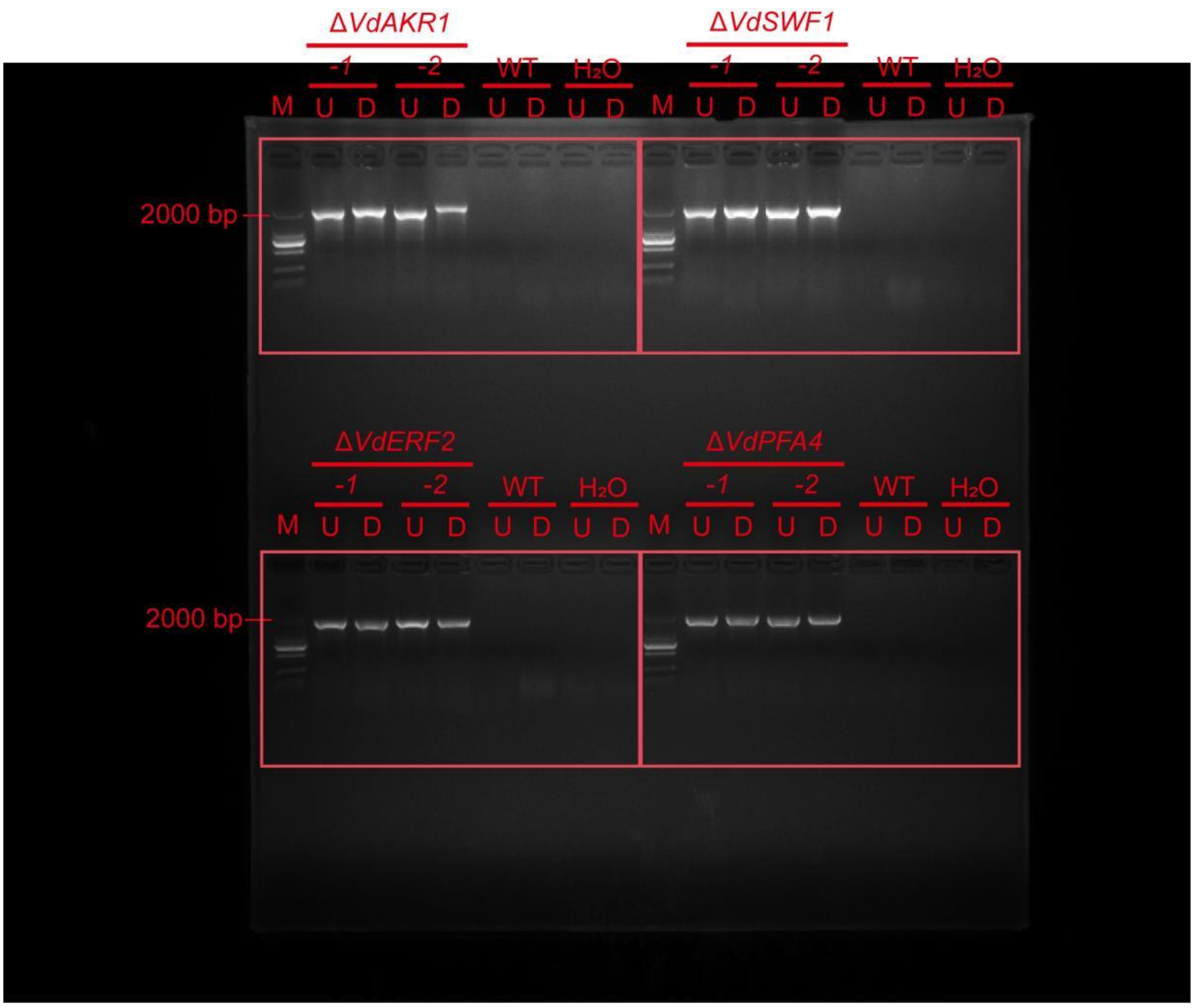

Figure S3B

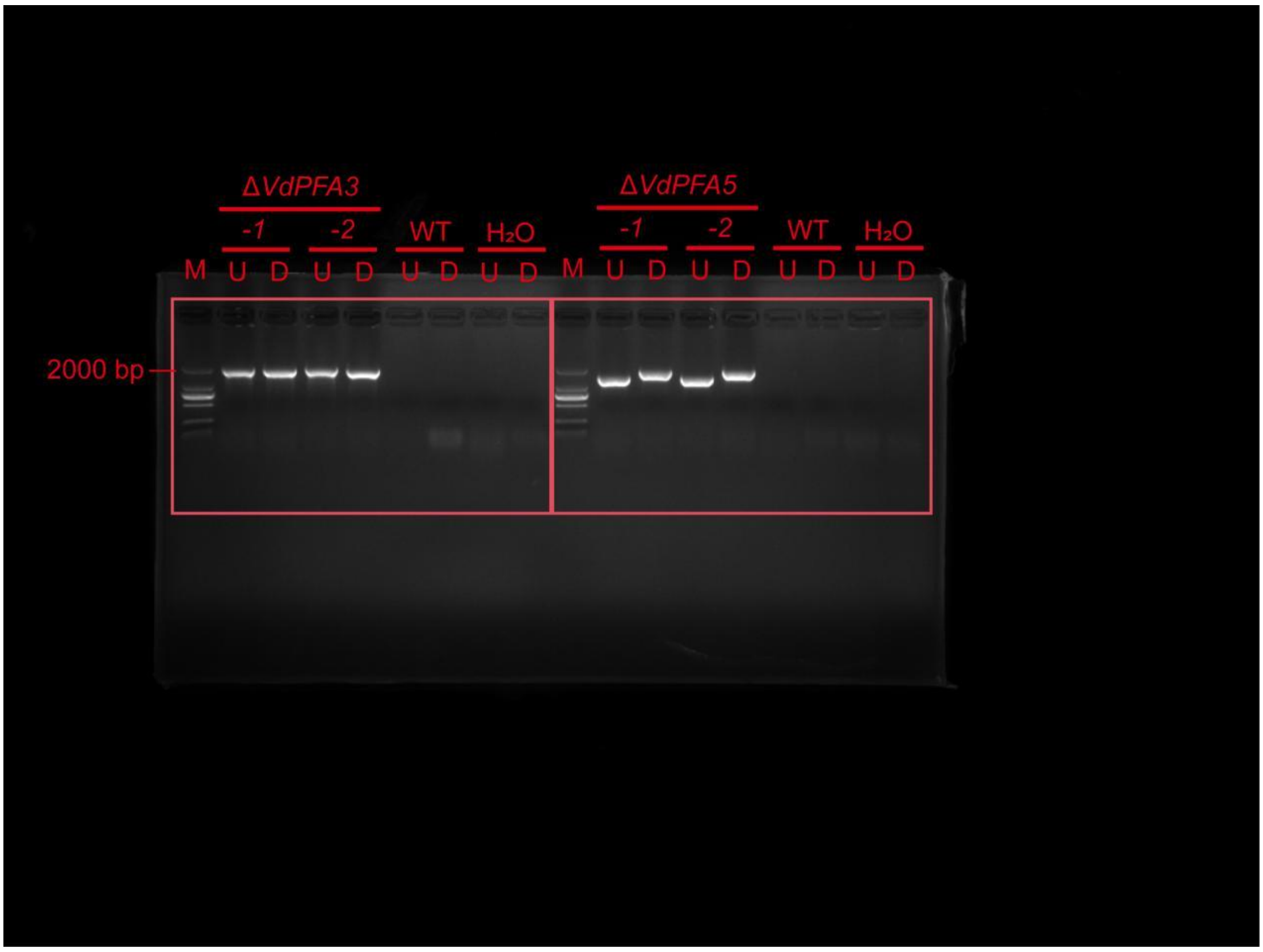

Figure S3B

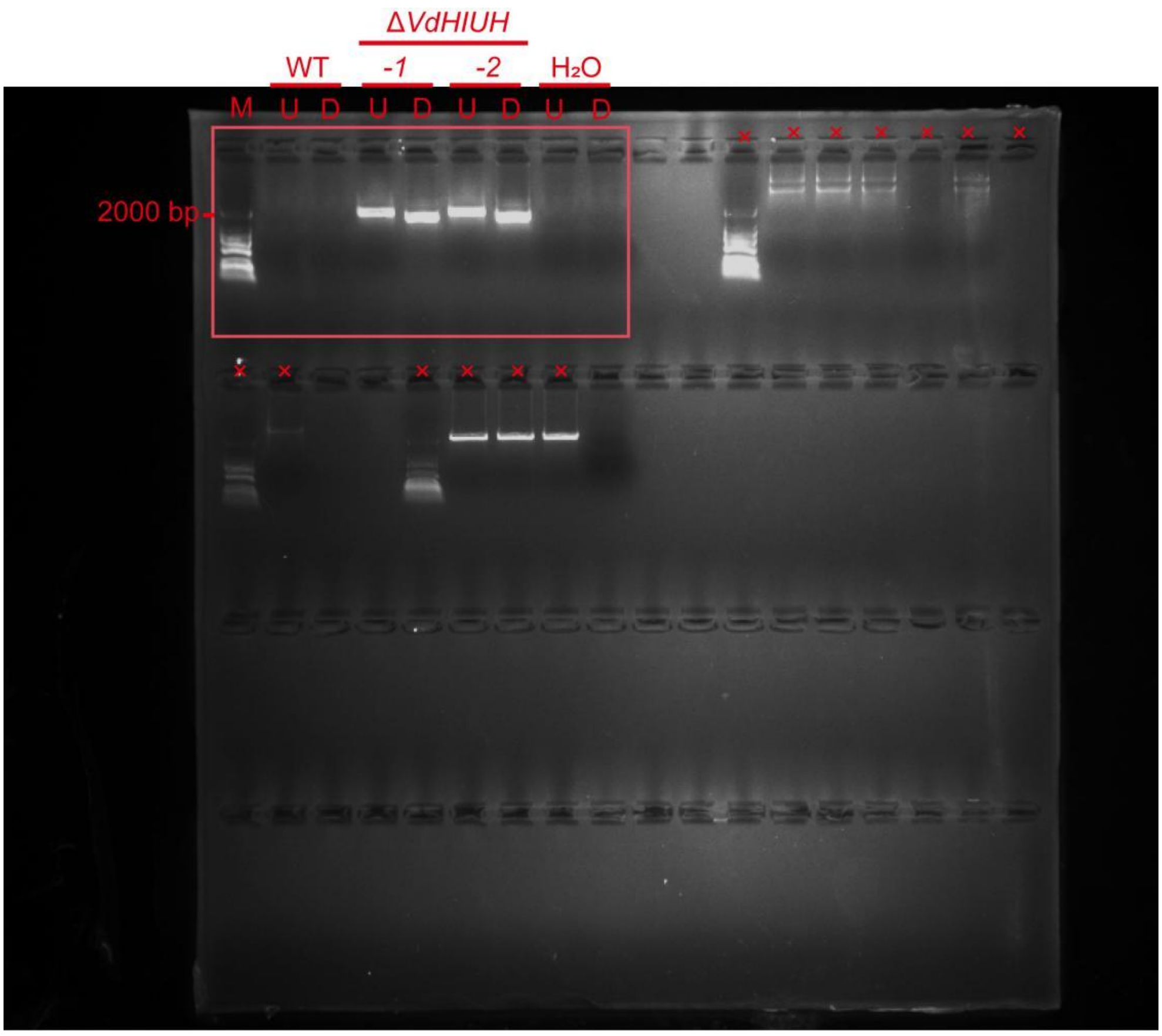

Figure S3C

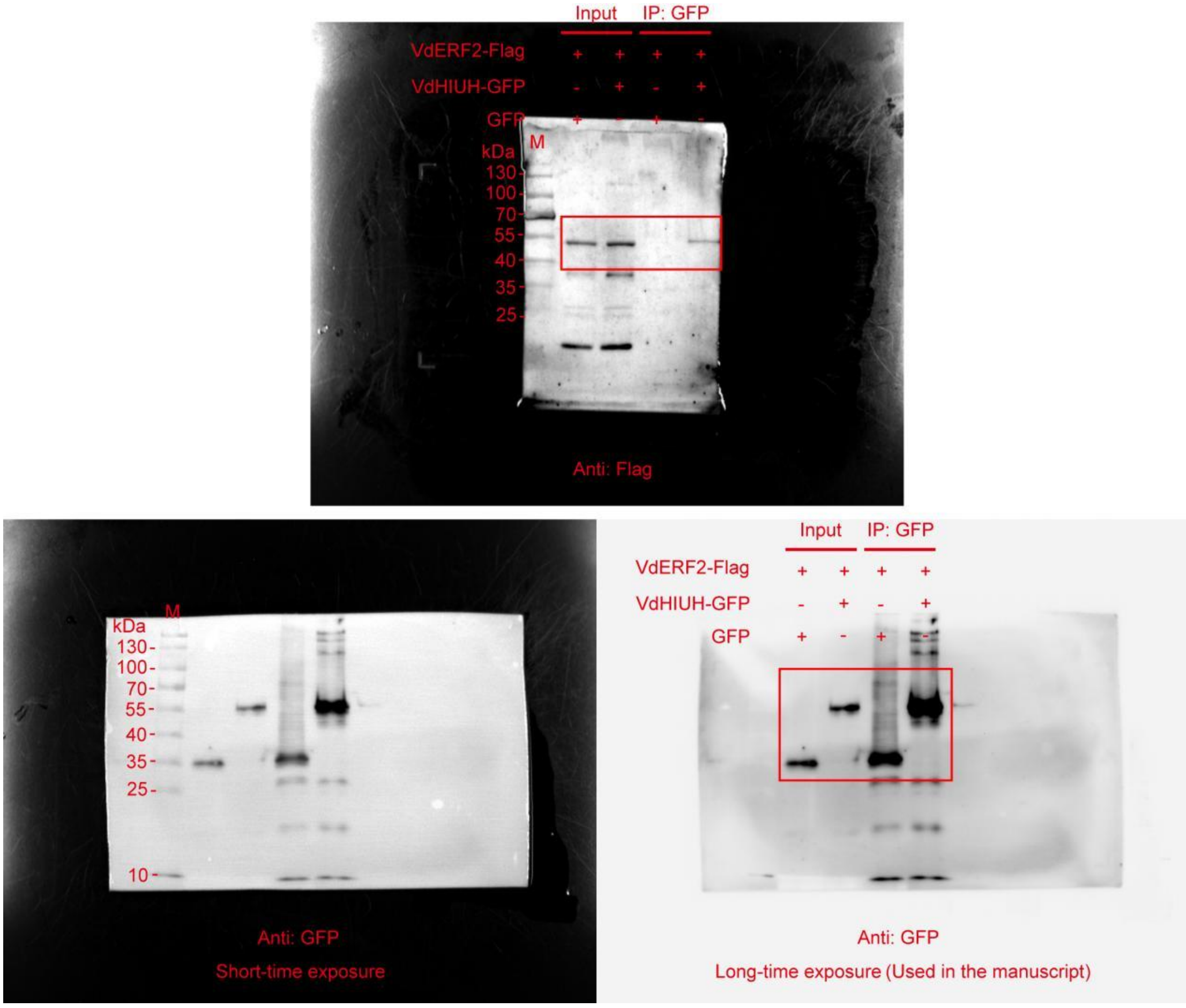
Input
IP: GFP
VdERF2-Flag
VdHIUH-GFP
GFP
kDa
M
130
100
70
55
40
35
25
Anti: Flag
10
Anti: GFP
Short-time exposure
Long-time exposure (Used in the manuscript)

Figure S6

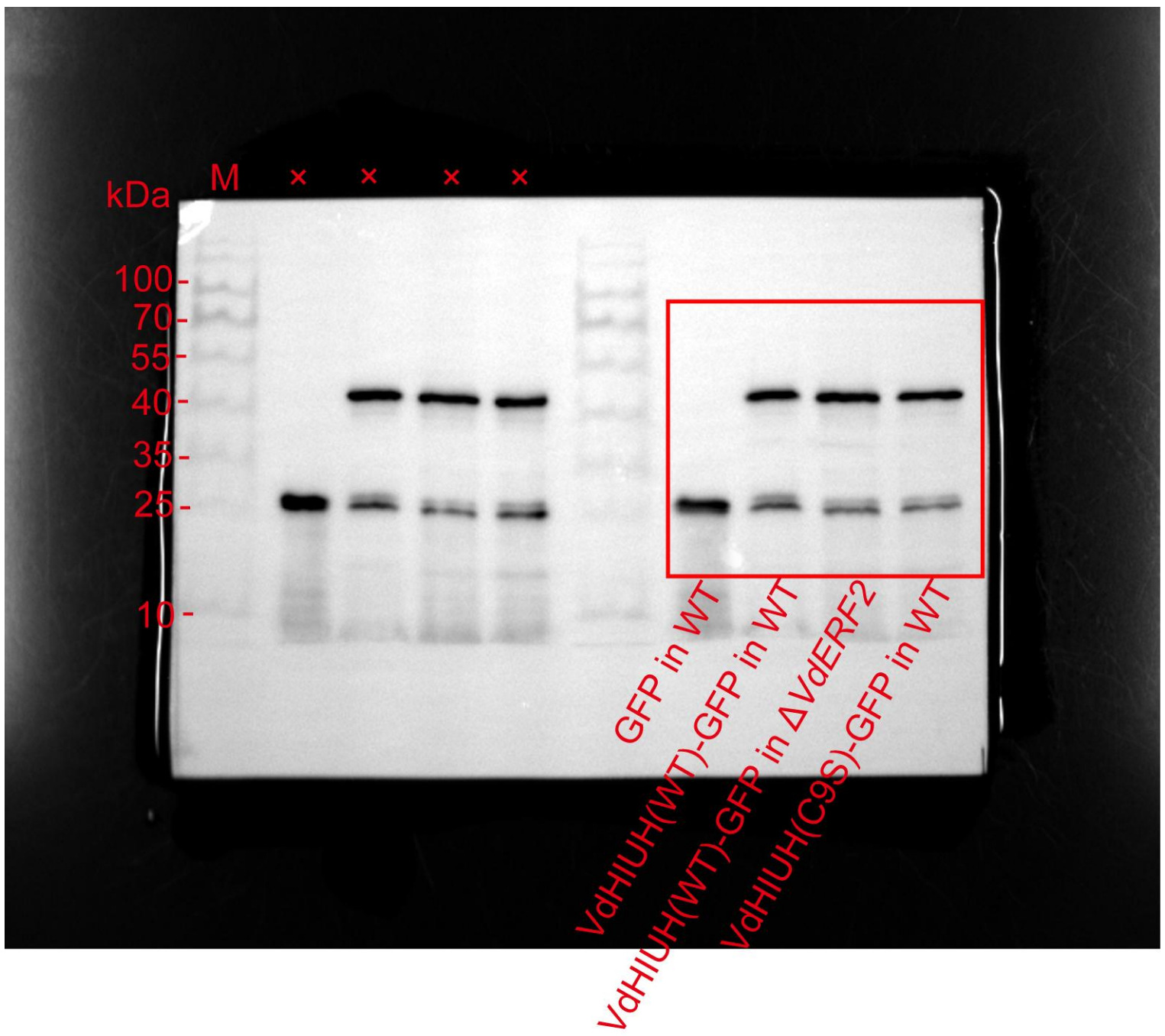
kDa
M
100-
70-
55-
40-
35-
25-
10-
GFP in WT
VdHIUH(WT)-GFP in WT
VdHIUH(WT)-GFP in ΔVdERF2
VdHIUH(C9S)-GFP in WT

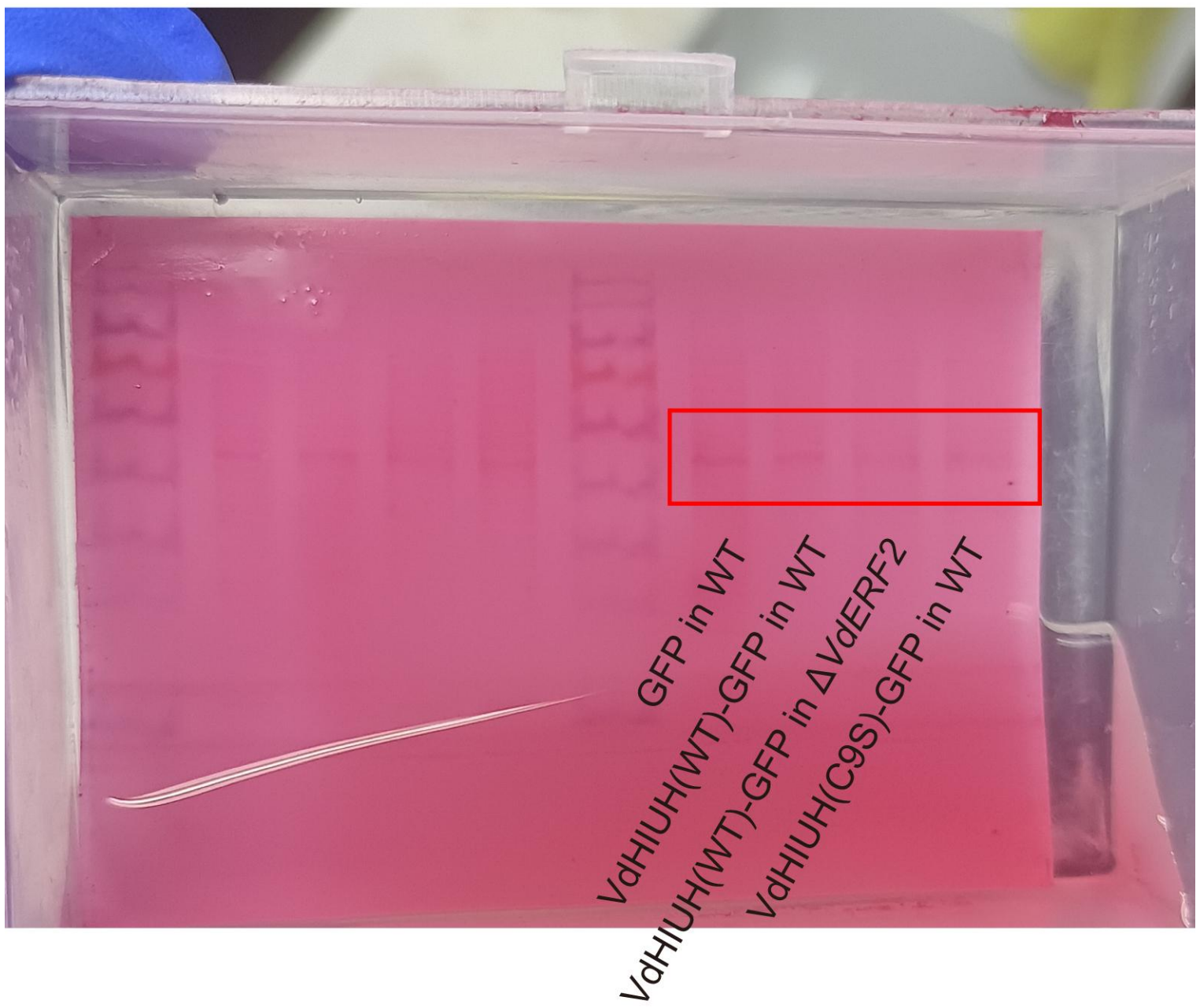
GFP in WT
VdHIUH(WT)-GFP in WT
VdHIUH(WT)-GFP in ΔVdERF2
VdHIUH(C9S)-GFP in WT

Figure S8B

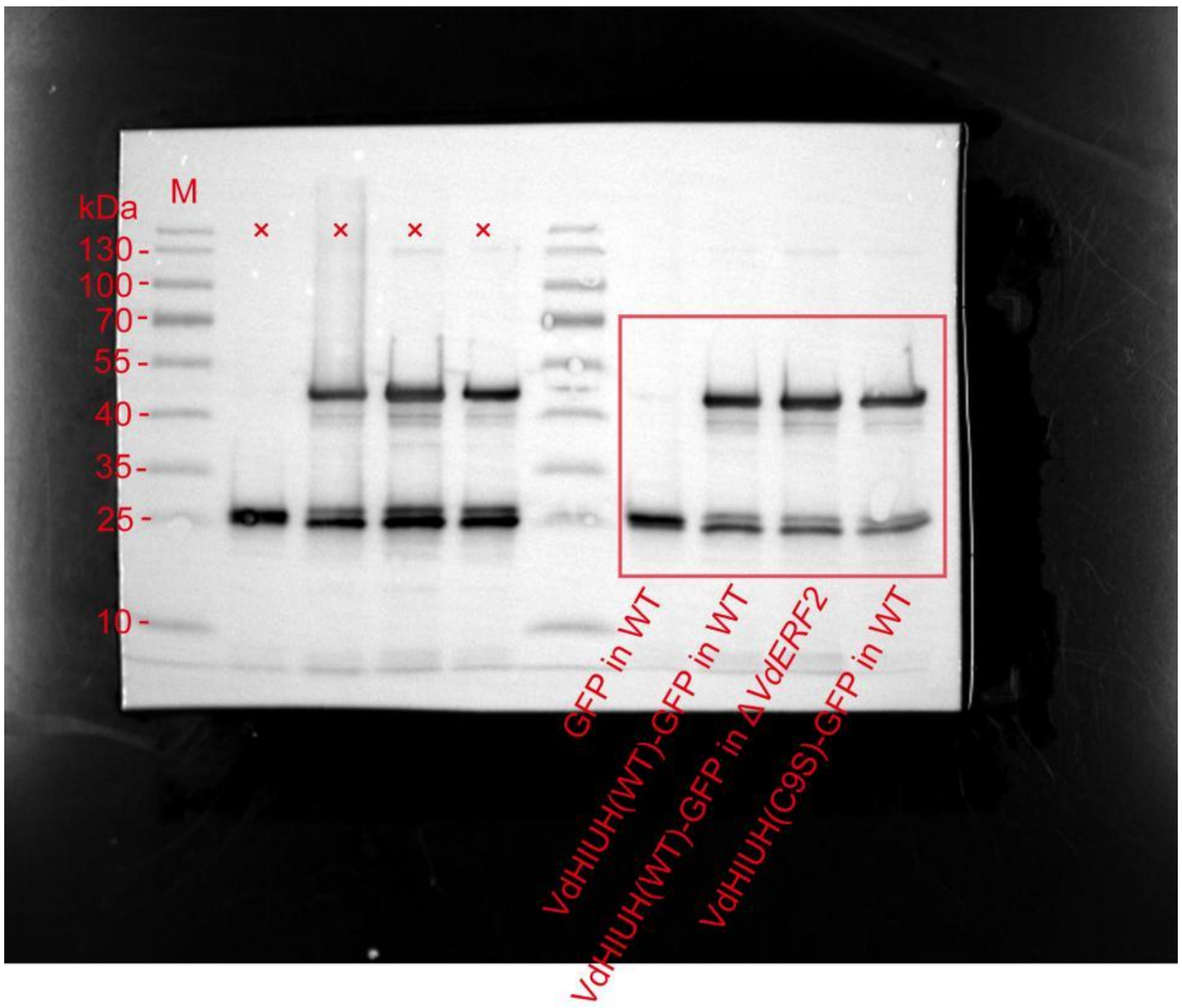
kDa
M
130
100
70
55
40
35
25
10
×
×
×
×
GFP in WT
VdHIUH(WT)-GFP in WT
VdHIUH(WT)-GFP in ΔVdERF2
VdHIUH(C9S)-GFP in WT

Figure S8E
