# Supplementary material for: Comparative S-acyl-proteomics reveals post-translational modulation of fungal purine metabolism for Verticillium wilt
Source: PLoS Pathog. 2026 Oct 26;22(10):e1014730. doi: 10.1371/journal.ppat.1014730 (PMC13722310; doi:10.1371/journal.ppat.1014730)
Supplement: Supplementary file 12 [file ppat.1014730.s015.docx]

**RESPONSE TO REVIEWERS**

Reviewer's Responses to Questions

**Part I - Summary**
Please use this section to discuss strengths/weaknesses of study, novelty/significance, general execution and scholarship.

**Reviewer #1:** This study investigates how the regulation of purine metabolism contributes to the development and pathogenicity of Verticillium dahliae. The authors identified that VdERF2, a protein S-acyltransferase, is essential for the fungus's normal development and virulence. VdERF2 mediates the S-acylation of VdHIUH, an enzyme in the purine metabolism pathway, which requires this modification to fully function in converting uric acid to allantoin. The production of allantoin helps reduce elevated reactive oxygen species (ROS) levels, thereby mitigating oxidative stress and allowing the fungus to maintain its ability to infect host plants. Ultimately, the works demonstrate how S-acylation modulates purine metabolism to control fungal pathogenicity.

**RESPONSE: Thank you very much for your insightful and constructive comments. In response to your valuable suggestions, we have carefully revised the manuscript. Our point-by-point responses are provided below.**

**Reviewer #2:** This manuscript by Li et al. investigates the role of protein S-acylation in regulating purine metabolism and pathogenicity in Verticillium dahliae. Through comparative S-acyl proteomics, the authors identify VdERF2 as a key PAT enzyme and propose that S-acylation of VdHIUH modulates uric acid metabolism, ROS homeostasis, and fungal virulence. The study is interesting, here are some points for improvement:

**RESPONSE: Thank you very much for your insightful and constructive comments. In response to your valuable suggestions, we have carefully revised the manuscript. Our point-by-point responses are provided below.**

1. Conservation of PATs is not rigorously demonstrated. The authors state that VdPATs are conserved across fungi , but whether all homologs retain the DHHC motif is not discussed. Whether function is conserved? It is good to provide sequence conservation and clarify whether this represents a conserved or species-specific mechanism. How about DHHC motif? These homologs also contain the motif?

**RESPONSE: Thank you very much. In accordance with your suggestion, we have conducted sequence alignments of the DHHC-CRD domain across PAT proteins from multiple plant-pathogenic fungi (Fig S2). Our analysis reveals that PAT proteins from a broad spectrum of phytopathogenic fungi harbor highly conserved DHHC/DHYC catalytic motifs, indicating that the S-acylation machinery is widely preserved among these pathogens. However, whether the VdERF2-VdHIUH regulatory axis identified in *V. dahliae* represents an evolutionarily conserved virulence mechanism remains to be determined and warrants further functional validation in additional fungal pathogens. We have incorporated this point into the Discussion section.**

2. Focus on VdERF2 is not well justified. VdERF2 is prioritized based on developmental phenotype, but VdAKR1 also shows strong defects. No systematic comparison of virulence across PAT mutants.

**RESPONSE: Thank you very much. During the initial phase of our study, we systematically evaluated all *VdPAT* mutants and identified *VdERF2* deletion as causing the most severe impairment in both fungal development and virulence. Consequently, we prioritized the mechanistic characterization of VdERF2 in this work. In accordance with your suggestion, we have now incorporated a comprehensive comparative data of the virulence phenotypes of all *VdPAT* mutants in Fig S4. This analysis revealed that most of tested *VdPAT* genes contribute to the virulence of *V. dahliae*, albeit to varying extents. Notably, the deletion of *VdERF2* resulted in the most pronounced attenuation of virulence among all mutants, thereby providing the principal justification for our focused investigation into the functional mechanism of VdERF2.**

3.the importance of purine metabolism to virulence is poorly discussed. Not sure if the pathways is modulated by S-acylation is also a general phenomenon.

**RESPONSE: Thank you very much! We have incorporated a discussion on the role of purine metabolism in fungal virulence into the Discussion section.**

4. The central model relies on VdHIUH, yet the conservation of HIUH and its S-acylation site is not demonstrated, and it remains unclear whether this mechanism extends beyond V. dahliae. The authors should provide cross-species conservation analysis or temper claims of generality. How much we know about HIUH? Any previous reports about this gene, any other plant fungi except C. neoformans?

**RESPONSE: Thank you very much! In accordance with your suggestion, we have incorporated a cross-species conservation analysis of VdHIUH homologs across multiple phytopathogenic fungi (Fig S7). Sequence alignment reveals that VdHIUH homologs are widely distributed among plant-pathogenic fungi, and the N-terminal S-acylation site (C9), identified in *V. dahliae*, is evolutionarily conserved across these species. To date, functional characterization of HIUH has been largely confined to the human pathogen *C. neoformans*, where it functions in the purine degradation pathway. However, no functional studies on HIUH homologs in other phytopathogenic fungi have been reported. In the revised manuscript, we explicitly note that whether the VdERF2-VdHIUH regulatory axis is conserved in other fungal pathogens remains an open question and warrants further investigation in future studies.**

**Reviewer #3:** This is a solid, well-executed study showing that the S-acyltransferase VdERF2 controls development and virulence in Verticillium dahliae via S-acylation of the purine degradation enzyme VdHIUH. Using comparative S-acyl-proteomics, genetics, biochemistry, and molecular dynamics, the authors demonstrate that VdERF2-mediated S-acylation at C9 enhances VdHIUH activity, reduces uric acid accumulation, and maintains ROS homeostasis through allantoin. The work establishes a direct link between protein lipidation, central metabolism, and fungal pathogenicity. The experiments are rigorous, the data consistent, and the writing clear. The topic fits PLOS Pathogens scope and will be of broad interest.

**RESPONSE: Thank you very much for your insightful and constructive comments. In response to your valuable suggestions, we have carefully revised the manuscript. Our point-by-point responses are provided below.**

**Part II – Major Issues: Key Experiments Required for Acceptance**
Please use this section to detail the key new experiments or modifications of existing experiments that should be absolutely required to validate study conclusions.

Generally, there should be no more than 3 such required experiments or major modifications for a "Major Revision" recommendation. If more than 3 experiments are necessary to validate the study conclusions, then you are encouraged to recommend "Reject".

**Reviewer #1:** The authors conclude that they have uncovered a critical molecular mechanism by which S-acylation modulates purine metabolism to control pathogenicity. However, the fact that allantoin acts as an ROS scavenger to alleviate oxidative stress is already a widely recognized phenomenon across multiple biological kingdoms, including plants and microbes. Therefore, simply demonstrating that ROS suppression via purine metabolism maintains virulence may lack the high degree of novelty expected by PLOS Pathogens. The true originality of this study is strictly limited to the upstream regulatory mechanism—specifically, VdERF2-mediated S-acylation. To strengthen the manuscript's impact, the authors must discuss whether this specific S-acylation regulatory mechanism is evolutionarily conserved among other pathogenic fungi.

**RESPONSE: Thank you very much! To address your suggestion concerning evolutionary conservation, we conducted sequence comparisons of the DHHC-CRD domain of PAT proteins across diverse pathogenic fungi. Our analysis demonstrates that the DHHC motifs in ERF2 homologs are highly conserved among these pathogens (Fig S2). Furthermore, comprehensive sequence alignment reveals that VdHIUH homologs are broadly distributed among plant-pathogenic fungi, and the N-terminal S-acylation site (C9), identified in *V. dahliae* in our current study, is evolutionarily conserved across this group (Fig S7). Collectively, these findings point to a potentially conserved regulatory module; however, its functional conservation remains to be experimentally validated in additional fungal pathogens. We have incorporated this discussion into the revised manuscript.**

The experiments using comparative S-acyl-proteomic analyses to identify targets and tracing the resulting ROS accumulation defect back to VdHIUH is generally logical. However, the manuscript explicitly states that VdERF2-regulated S-acylation is significantly enriched across various metabolic pathways, not just purine metabolism. Despite this, the authors oversimplify their conclusion by attributing the severe pathogenicity defects of VdERF2 mutants entirely to the functional loss of a single target, VdHIUH. Given that VdERF2 likely possesses numerous S-acylation targets, definitively linking the entire mutant phenotype solely to VdHIUH and purine metabolism is a significant logical leap that requires further justification.

**RESPONSE: Thank you very much! Your insight is correct. As demonstrated, S-acylation of a number of potential protein substrates was significantly reduced in the *VdERF2* mutant, suggesting that this PAT may participate in diverse biological processes. Notably, GO analysis revealed a prominent enrichment of terms associated with purine and nucleotide; accordingly, we prioritized this functional category and identified VdHIUH as a key target protein. Following your valuable suggestion, we conducted additional experiments as outlined below. As you anticipated, our results substantiate that VdHIUH is a critical substrate that partially mediates the biological function of VdERF2; however, the functional characterization of other candidate substrates remains an important objective for future studies. This point has been explicitly clarified in the revised manuscript.**

To build a more robust scientific case and address the flaws mentioned above, I strongly recommend the following improvements.
1) Direct proof of single-target dependency: To rule out interference from other metabolic pathways affected by VdERF2, the authors must demonstrate whether overexpressing an artificially active (S-acylation-independent) version of VdHIUH in a VdERF2 deletion mutant can fully rescue the ROS suppression and pathogenicity defects.

**RESPONSE: Thank you very much! Because S-acylation mediated by VdERF2 enhances the enzymatic activity of VdHUIH, increasing the protein level of VdHUIH may compensate for its reduced activity. Following your suggestion, we overexpressed VdHUIH in both the WT strain and the Δ*VdERF2* mutant and assessed fungal growth and pathogenicity. As shown in Fig S8, VdHUIH overexpression in the WT background had no discernible effect on fungal development or virulence. In contrast, VdHUIH overexpression partially rescued the growth and pathogenicity defects associated with *VdERF2* deletion. These findings support the notion that VdHUIH functions as a key S-acylation substrate and partially mediates the biological functions of VdERF2.**

2) Mapping target cysteine residues: The authors need to provide biochemical and molecular data identifying the exact cysteine residue(s) on VdHIUH where S-acylation occurs. Point mutation analyses of these specific residues are necessary to definitively prove the structural and functional changes in enzymatic activity.

**RESPONSE:Thank you very much! We sincerely apologize for the lack of clarity in our previous description. In Fig 3, biochemical analysis identified Cys9 as the S-acylation site on VdHIUH. Subsequent phenotypic analyses further demonstrate that this residue is essential for VdHIUH’s role in fungal development and virulence. These findings obtained using an S-acylation-deficient VdHIUH mutant are consistent with the functional role of VdERF2. Moreover, bioinformatic analyses reveal that this cysteine residue is evolutionarily conserved across a broad spectrum of pathogenic fungi (Fig S7).**

3) In planta infection stress data: The mechanism of restoring normal development and maintaining pathogenicity via ROS mitigation must be thoroughly validated under actual host infection conditions (in planta). It is critical to demonstrate that this allantoin-mediated mechanism effectively counteracts the massive oxidative burst generated by the plant's active immune response, rather than merely observing ROS reduction in an in vitro culture environment.

**RESPONSE: Thank you very much! Following your suggestion, we conducted in planta assays using cotton seedlings (Fig S10). Cotton plants were with allantoin prior to inoculation with the WT, Δ*VdERF2*, or Δ*VdHIUH* strains. Allantoin treatment markedly suppressed H₂O₂ accumulation in cotton plants inoculated with either the Δ*VdERF2* or Δ*VdHIUH* strain. In contrast, allantoin treatment induced elevated H₂O₂ levels in cotton infected with the WT strain; this H₂O₂ signal likely originates from both fungal and plant cells. Importantly, cotton plants inoculated with Δ*VdERF2* or Δ*VdHIUH* exhibited significantly greater disease severity under allantoin treatment compared with the DMSO-treated control group. Collectively, these in planta results demonstrate that allantoin functionally compensates for the loss of VdERF2 and VdHIUH during pathogenesis.**

**Reviewer #2:** (No Response)

**Reviewer #3:** Is there any interaction between VdERF2 and VdHIUH?

**RESPONSE: Thank you very much! In accordance with your suggestion, we performed a co-immunoprecipitation assay, which demonstrated that VdERF2 interacts with VdHIUH (Fig S6), supporting the notion that VdHIUH is a substrate protein of VdERF2.**

**Part III – Minor Issues: Editorial and Data Presentation Modifications**
Please use this section for editorial suggestions as well as relatively minor modifications of existing data that would enhance clarity.

**Reviewer #1:** (No Response)

**Reviewer #2:** (No Response)

**Reviewer #3:**

Title: Comparable → Comparative
line 22. modulates protein property and function → properties and functions
line 31. enhances VdHIUH activity → enhances the activity of VdHIUH
line 102. This discovery would→This discovery establishes
line 107. VdERF2 is PAT essential→ VdERF2 is a PAT that is essential
line 409. Using a similar approach, the similar method→Using a similar approach

**RESPONSE: Thank you very much! We have reviewed the manuscript and addressed the identified grammatical errors and typographical inconsistencies.**
