# Supplementary material for: Comparative S-acyl-proteomics reveals post-translational modulation of fungal purine metabolism for Verticillium wilt
Source: PLoS Pathog. 2026 Oct 26;22(10):e1014730. doi: 10.1371/journal.ppat.1014730 (PMC13722310; doi:10.1371/journal.ppat.1014730)
Supplement: Supplementary file 13 [file ppat.1014730.s016.docx]

**RESPONSE TO REVIEWERS**

PPATHOGENS-D-26-00375R1

Comparative S-acyl-proteomics reveals post-translational modulation of fungal purine metabolism for Verticillium wilt
PLOS Pathogens

Dear Dr. Lai,

Thank you for submitting your manuscript to PLOS Pathogens. After careful consideration, we feel that it has merit but does not fully meet PLOS Pathogens's publication criteria as it currently stands. Reviewer 1 raises some important points that can likely be addressed without too much additional work. Therefore, we invite you to submit a revised version of the manuscript that addresses these concerns.

Please submit your revised manuscript by Nov 14 2026 11:59PM. If you will need more time than this to complete your revisions, please reply to this message or contact the journal office at plospathogens@plos.org. When you're ready to submit your revision, log on to https://www.editorialmanager.com/ppathogens/ and select the 'Submissions Needing Revision' folder to locate your manuscript file.

Please include the following items when submitting your revised manuscript:

* A letter that responds to each point raised by the editor and reviewer(s). You should upload this letter as a separate file labeled 'Response to Reviewers'. This file does not need to include responses to any formatting updates and technical items listed in the 'Journal Requirements' section below.
* A marked-up copy of your manuscript that highlights changes made to the original version. You should upload this as a separate file labeled 'Revised Manuscript with Track Changes'.
* An unmarked version of your revised paper without tracked changes. You should upload this as a separate file labeled 'Manuscript'.

If you would like to make changes to your financial disclosure, competing interests statement, or data availability statement, please make these updates within the submission form at the time of resubmission. Guidelines for resubmitting your figure files are available below the reviewer comments at the end of this letter.

As the corresponding author, your ORCID iD is verified in the submission system and will appear in the published article. PLOS supports the use of ORCID, and we encourage all coauthors to register for an ORCID iD and use it as well. Please encourage your coauthors to verify their ORCID iD within the submission system before final acceptance, as unverified ORCID iDs will not appear in the published article. Only the individual author can complete the verification step; PLOS staff cannot verify ORCID iDs on behalf of authors.

We look forward to receiving your revised manuscript.

Kind regards,

Richard A Wilson
Academic Editor
PLOS Pathogens

Robert Cramer
Section Editor
PLOS Pathogens

Sumita Bhaduri-McIntosh
Editor-in-Chief
PLOS Pathogens
orcid.org/0000-0003-2946-9497

Dominique Soldati-Favre
Editor-in-Chief
PLOS Pathogens
orcid.org/0000-0003-4156-2109

**Journal Requirements:**

1. We noted that you have included an author contributions statement in your manuscript file, and it appears to differ from the information we have on file in your submission form. Please remove the statement from your manuscript and instead ensure that the full contributions of each author are acknowledged in the ""Add/EditRemove Authors"" section of our submission form.

The list of CRediT author contributions may be found here: https://journals.plos.org/plospathogens/s/authorship#loc-author-contributions.

**RESPONSE: Thank you very much! The description of author contributions has been revised in the manuscript and the submission system.**

2. Modern Agro-industry Technology
   Research System (2023KJ114 to CY), the Program for Changjiang Scholars (N/A to
   CY), and Fund for young scholars from South China Normal University (24KJ17 to CL).
   The funders had no role in study design, data collection and analysis, decision to
   publish, or preparation of the manuscript.”. However, your funding information on the submission form indicates that you received funding from “Guangdong S&T Program        (2025B0202070003 to Prof. Jianbin Lai)”. Please indicate by return email the full and correct funding information for your study and confirm the order in which funding contributions should appear. Please be sure to indicate whether the funders played any role in the study design, data collection and analysis, decision to publish, or preparation of the manuscript.

**RESPONSE: Thank you very much! The funding information in the manuscript has been confirmed. The full and correct funding information has been sent by email.**

3. Some material included in your submission may be copyrighted. According to PLOS’s copyright policy, authors who use figures or other material (e.g., graphics, clipart, maps) from another author or copyright holder must demonstrate or obtain permission to publish this material under the Creative Commons Attribution 4.0 International (CC BY 4.0) License used by PLOS journals. Please closely review the details of PLOS’s copyright requirements here: PLOS Licenses and Copyright. If you need to request permissions from a copyright holder, you may use PLOS's Copyright Content Permission form.

Please respond directly to this email and provide any known details concerning your material's license terms and permissions required for reuse, even if you have not yet obtained copyright permissions or are unsure of your material's copyright compatibility. Once you have responded and addressed all other outstanding technical requirements, you may resubmit your manuscript within Editorial Manager.

**RESPONSE: Thank you very much! With the exception of the image in Figure S8D, regarding which we previously submitted formal permission and copyright documentation, no other figures raise copyright concerns. As noted in your comments, we confirm that the schematic in Figure 2 was drawn by hand by the authors, and that the authors are the original photographers of Figures 1, 3, S4, and S9. The information has been sent by email.**

Potential Copyright Issues:

Figure 2: Please confirm whether you drew the images / clip-art within the figure panels by hand. If you did not draw the images, please provide (a) a link to the source of the images or icons and their license / terms of use; or (b) written permission from the copyright holder to publish the images or icons under our CC BY 4.0 license. Alternatively, you may replace the images with open source alternatives. See these open source resources you may use to replace images / clip-art:
- https://commons.wikimedia.org
- https://openclipart.org/

**RESPONSE: Thank you very much! We confirmed that we drew the images in Figure 2 by hand.**

Fig 1, 3, S4, S9: Please confirm (a) that you are the photographer; or (b) provide written permission from the photographer to publish the photo(s) under our CC BY 4.0 license.

**RESPONSE: Thank you very much! We confirmed that we are the photographer of these images.**

If the reviewer comments include a recommendation to cite specific previously published works, please review and evaluate these publications to determine whether they are relevant and should be cited. There is no requirement to cite these works unless the editor has indicated otherwise.

[Note: HTML markup is below. Please do not edit.]

**Reviewers' Comments:**

Reviewer's Responses to Questions

**Part I - Summary**
Please use this section to discuss strengths/weaknesses of study, novelty/significance, general execution and scholarship.

Reviewer #1: The authors have responded constructively to the previous round. All three experiments I listed as required for acceptance were attempted, two of them generating new data (Fig S9, Fig S10), and the third point (mapping of the acylated cysteine) was already supported by data present in the original submission, which I had overlooked. The addition of the VdERF2–VdHIUH co-immunoprecipitation (Fig S6), the systematic virulence comparison across all VdPAT mutants (Fig S4), and the cross-species conservation analyses (Fig S2, Fig S7) meaningfully strengthen the manuscript. Importantly, the authors have toned down the central causal claim, now stating that VdHIUH partially mediates the biological function of VdERF2, which directly addresses my concern about attributing the entire ΔVdERF2 phenotype to a single substrate.

**RESPONSE: Thank you very much for your kind comments!**

Reviewer #2: I dont have any questions. I have reviewed the questions and authors' revision.

**RESPONSE: Thank you very much for your kind comments!**

Reviewer #3: The authors have addressed my comments. I have no more questions.

**RESPONSE: Thank you very much for your kind comments!**

**Part II – Major Issues: Key Experiments Required for Acceptance**
Please use this section to detail the key new experiments or modifications of existing experiments that should be absolutely required to validate study conclusions.

Generally, there should be no more than 3 such required experiments or major modifications for a "Major Revision" recommendation. If more than 3 experiments are necessary to validate the study conclusions, then you are encouraged to recommend "Reject".

Reviewer #1: My main remaining concern is the allantoin rescue experiments. Allantoin is a major nitrogen source for filamentous fungi, catabolised via allantoinase and allantoicase to urea and ammonia. Loss of VdHIUH, or loss of its VdERF2-dependent S-acylation, blocks flux from uric acid to allantoin and therefore blocks access to purine-derived nitrogen. Under this interpretation, exogenous allantoin would restore growth of the ΔVdERF2, ΔVdHIUH and ECVdHIUH(C9S) strains purely as a nutrient, with no antioxidant contribution required. Critically, this alternative also predicts the authors' own control result — that allantoin has no effect on the wild type — because the wild type already produces allantoin endogenously. The observation that allantoin lowers H₂O₂ is likewise compatible with either model, since restoring flux through the pathway would relieve uric acid accumulation regardless of mechanism.

As the data stand, the two explanations are not separable, yet the Discussion states that the allantoin rescue is "confirming that ROS accumulation is a primary cause of their abnormal development."

I would accept either of the following:

(a) A discriminating control. A single plate-based experiment would resolve this: test whether a non-metabolisable antioxidant (N-acetylcysteine, ascorbate, or reduced glutathione) reproduces the growth rescue, and/or whether an equimolar alternative nitrogen source (urea or ammonium) fails to do so. If the antioxidant rescues and the alternative nitrogen source does not, the ROS model is substantially strengthened.

(b) Explicit acknowledgement and downgrading. If the authors prefer not to perform this experiment, the nitrogen-nutrition alternative must be stated explicitly in the Discussion as an unexcluded possibility, and all causal ROS language must be softened accordingly.

**RESPONSE: Thank you very much! Pursuant to your suggestion, we have selected option (b) and revised the Discussion section to explicitly state the nitrogen-nutrition alternative while moderating the statements concerning ROS.**

Reviewer #2: (No Response)

**RESPONSE: Thank you very much!**

Reviewer #3: (No Response)

**RESPONSE: Thank you very much!**

**Part III – Minor Issues: Editorial and Data Presentation Modifications**
Please use this section for editorial suggestions as well as relatively minor modifications of existing data that would enhance clarity.

Reviewer #1: In Fig 4F the allantoin effect is approximately 21 → 22 mm for ΔVdERF2 and 21.5 → 22.5 mm for ECVdHIUH(C9S), against a wild-type value of roughly 23 mm. The differences are statistically significant but the mutants are not restored to wild-type level. "Restore" should be replaced with "partially alleviate" throughout, including in the Abstract.

**RESPONSE: Thank you very much! “Restore” has been replaced with “partially alleviate” throughout the manuscript.**

A clear residual band is visible in the C9S pulldown (+NH₂OH) lane, and the quantification indicates roughly 70% reduction rather than near-complete loss. This residual signal is itself informative: it suggests one or more additional, minor S-acylation sites, which is directly relevant to my earlier request for identification of the acylated residue(s). Please describe the result as it appears and note the implication.

**RESPONSE: Thank you very much! Following your suggestion, the description of the result presented in Figure 3B has been revised in the manuscript.**

Reviewer #2: (No Response)

**RESPONSE: Thank you very much!**

Reviewer #3: (No Response)

**RESPONSE: Thank you very much!**

PLOS authors have the option to publish the peer review history of their article (what does this mean?). If published, this will include your full peer review and any attached files.


If you choose “no”, your identity will remain anonymous but your review may still be made public.


**Do you want your identity to be public for this peer review?** For information about this choice, including consent withdrawal, please see our Privacy Policy.

Reviewer #1: **Yes:** Junhyun Jeon

Reviewer #2: No

Reviewer #3: No

[NOTE: If reviewer comments were submitted as an attachment file, they will be attached to this email and accessible via the submission site. Please log into your account, locate the manuscript record, and check for the action link "View Attachments". If this link does not appear, there are no attachment files.]

**Figure resubmission:**

While revising your submission, we strongly recommend that you use PLOS’s NAAS tool (https://ngplosjournals.pagemajik.ai/artanalysis) to test your figure files. NAAS can convert your figure files to the TIFF file type and meet basic requirements (such as print size, resolution), or provide you with a report on issues that do not meet our requirements and that NAAS cannot fix.

After uploading your figures to PLOS’s NAAS tool - https://ngplosjournals.pagemajik.ai/artanalysis, NAAS will process the files provided and display the results in the "Uploaded Files" section of the page as the processing is complete. If the uploaded figures meet our requirements (or NAAS is able to fix the files to meet our requirements), the figure will be marked as "fixed" above. If NAAS is unable to fix the files, a red "failed" label will appear above. When NAAS has confirmed that the figure files meet our requirements, please download the file via the download option, and include these NAAS processed figure files when submitting your revised manuscript.

**Reproducibility:**

To enhance the reproducibility of your results, we recommend that authors of applicable studies deposit laboratory protocols in protocols.io, where a protocol can be assigned its own identifier (DOI) such that it can be cited independently in the future. Additionally, PLOS ONE offers an option to publish peer-reviewed clinical study protocols. Read more information on sharing protocols at https://plos.org/protocols?utm_medium=editorial-email&utm_source=authorletters&utm_campaign=protocols

In compliance with data protection regulations, you may request that we remove your personal registration details at any time. (Remove my information/details). Please contact the publication office if you have any questions.
