# Supplementary material for: Comparative S-acyl-proteomics reveals post-translational modulation of fungal purine metabolism for Verticillium wilt
Source: PLoS Pathog. 2026 Oct 26;22(10):e1014730. doi: 10.1371/journal.ppat.1014730 (PMC13722310; doi:10.1371/journal.ppat.1014730)
Supplement: Peer review history

# Peer review: Decision Letter 0

**DOI:** 10.1371/journal.ppat.1014730.r001

**Authors:** Robert A. Cramer, Richard A Wilson

9 Jun 2026

Comparable S-acyl-proteomics reveals post-translational modulation of fungal purine metabolism for Verticillium wilt

PLOS Pathogens

Dear Dr. Lai,

Thank you for submitting your manuscript to PLOS Pathogens. I apologize for the time it has taken to complete our assessment, there were difficulties securing reviewers. After careful consideration, we feel that it has merit but does not fully meet PLOS Pathogens's publication criteria as it currently stands. Therefore, we invite you to submit a revised version of the manuscript that addresses the points raised during the review process. In particular, the study's main conclusion regarding the direct function of S-acylation on purine metabolism and virulence needs further support given the multitude of other S-acylation targets in the cell.

Please submit your revised manuscript by Aug 08 2026 11:59PM. If you will need more time than this to complete your revisions, please reply to this message or contact the journal office at plospathogens@plos.org. When you're ready to submit your revision, log on to https://www.editorialmanager.com/ppathogens/ and select the 'Submissions Needing Revision' folder to locate your manuscript file.

Please include the following items when submitting your revised manuscript:

* A letter that responds to each point raised by the editor and reviewer(s). You should upload this letter as a separate file labeled 'Response to Reviewers'. This file does not need to include responses to any formatting updates and technical items listed in the 'Journal Requirements' section below.

* A marked-up copy of your manuscript that highlights changes made to the original version. You should upload this as a separate file labeled 'Revised Manuscript with Track Changes'.

* An unmarked version of your revised paper without tracked changes. You should upload this as a separate file labeled 'Manuscript'.

We look forward to receiving your revised manuscript.

Kind regards,

Richard A Wilson

Academic Editor

PLOS Pathogens

Robert Cramer

Section Editor

Editor-in-Chief

PLOS Pathogens

orcid.org/0000-0003-2946-9497

Editor-in-Chief

PLOS Pathogens

orcid.org/0000-0002-7699-2064

**Additional Editor Comments (if provided):**

The reviewer raise valid concerns that must be addressed. In general, more direct proof to bolster the author's conclusions is warranted.

**Journal Requirements:**

If the reviewer comments include a recommendation to cite specific previously published works, please review and evaluate these publications to determine whether they are relevant and should be cited. There is no requirement to cite these works unless the editor has indicated otherwise.

1) Please ensure that the CRediT author contributions listed for every co-author are completed accurately and in full.

At this stage, the following Authors/Authors require contributions: Chi Li, Wenyang Zhang, Shuaifei Cui, Jian Zhang, Yuzhen Li, Xiaoshi Liu, Yang Li, Zhiying Wang, Wenjie Huang, Gongda Li, Chengwei Yang, Shijuan Yan, and Jianbin Lai. Please ensure that the full contributions of each author are acknowledged in the "Add/Edit/Remove Authors" section of our submission form.

The list of CRediT author contributions may be found here: https://journals.plos.org/plospathogens/s/authorship#loc-author-contributions

2) Please provide an Author Summary. This should appear in your manuscript between the Abstract (if applicable) and the Introduction, and should be 150-200 words long. The aim should be to make your findings accessible to a wide audience that includes both scientists and non-scientists. Sample summaries can be found on our website under Submission Guidelines:

https://journals.plos.org/plospathogens/s/submission-guidelines#loc-parts-of-a-submission

3) We do not publish any copyright or trademark symbols that usually accompany proprietary names, eg ©,  ®, or TM  (e.g. next to drug or reagent names). Therefore please remove all instances of trademark/copyright symbols throughout the text, including:

- TM on page: 17.

4) Please upload all main figures as separate Figure files in .tif or .eps format. For more information about how to convert and format your figure files please see our guidelines:

https://journals.plos.org/plospathogens/s/figures

5) Please upload a copy of Figure Fig 1-4 which you refer to in your text on pages 5, 6, 8, and 9. Or, if the figure is no longer to be included as part of the submission please remove all reference to it within the text.

6) Please amend your detailed Financial Disclosure statement. This is published with the article. It must therefore be completed in full sentences and contain the exact wording you wish to be published.

i) State the initials, alongside each funding source, of each author to receive each grant. For example: "This work was supported by the National Institutes of Health (####### to AM; ###### to CJ) and the National Science Foundation (###### to AM)."

ii) State what role the funders took in the study. If the funders had no role in your study, please state: "The funders had no role in study design, data collection and analysis, decision to publish, or preparation of the manuscript."

iii) If any authors received a salary from any of your funders, please state which authors and which funders..

If you did not receive any funding for this study, please simply state: u201cThe authors received no specific funding for this work.u201d

7) We have amended your Competing Interest statement to comply with journal style. We kindly ask that you double check the statement and let us know if anything is incorrect.

8) PLOS now requires that authors provide the original uncropped and unadjusted images underlying all blot or gel results reported in a submission’s figures or Supporting Information files. This policy and the journal’s other requirements for blot/gel reporting and figure preparation are described in detail at https://journals.plos.org/plosone/s/figures#loc-blot-and-gel-reporting-requirements and https://journals.plos.org/plosone/s/figures#loc-preparing-figures-from-image-files.

When you submit your revised manuscript, please ensure that your figures adhere fully to these guidelines and provide the original underlying images for all blot or gel data reported in your submission. See the following link for instructions on providing the original image data: https://journals.plos.org/plosone/s/figures#loc-original-images-for-blots-and-gels.

In your cover letter, please note whether your blot/gel image data are in Supporting Information or posted at a public data repository, provide the repository URL if relevant, and provide specific details as to which raw blot/gel images, if any, are not available. Email us at customercare@plos.org if you have any questions.

**Reviewers' Comments:**

Reviewer's Responses to Questions

**Part I - Summary**

Please use this section to discuss strengths/weaknesses of study, novelty/significance, general execution and scholarship.

Reviewer #1: This study investigates how the regulation of purine metabolism contributes to the development and pathogenicity of Verticillium dahliae. The authors identified that VdERF2, a protein S-acyltransferase, is essential for the fungus's normal development and virulence. VdERF2 mediates the S-acylation of VdHIUH, an enzyme in the purine metabolism pathway, which requires this modification to fully function in converting uric acid to allantoin. The production of allantoin helps reduce elevated reactive oxygen species (ROS) levels, thereby mitigating oxidative stress and allowing the fungus to maintain its ability to infect host plants. Ultimately, the works demonstrate how S-acylation modulates purine metabolism to control fungal pathogenicity.

Reviewer #2: This manuscript by Li et al. investigates the role of protein S-acylation in regulating purine metabolism and pathogenicity in Verticillium dahliae. Through comparative S-acyl proteomics, the authors identify VdERF2 as a key PAT enzyme and propose that S-acylation of VdHIUH modulates uric acid metabolism, ROS homeostasis, and fungal virulence. The study is interesting, here are some points for improvement:

1. Conservation of PATs is not rigorously demonstrated. The authors state that VdPATs are conserved across fungi , but whether all homologs retain the DHHC motif is not discussed. Whether function is conserved? It is good to provide sequence conservation and clarify whether this represents a conserved or species-specific mechanism. How about DHHC motif? These homologs also contain the motif?

2. Focus on VdERF2 is not well justified. VdERF2 is prioritized based on developmental phenotype, but VdAKR1 also shows strong defects. No systematic comparison of virulence across PAT mutants.

3.the importance of purine metabolism to virulence is poorly discussed. Not sure if the pathways is modulated by S-acylation is also a general phenomenon.

4. The central model relies on VdHIUH, yet the conservation of HIUH and its S-acylation site is not demonstrated, and it remains unclear whether this mechanism extends beyond V. dahliae. The authors should provide cross-species conservation analysis or temper claims of generality. How much we know about HIUH? Any previous reports about this gene, any other plant fungi except C. neoformans?

Reviewer #3: This is a solid, well-executed study showing that the S-acyltransferase VdERF2 controls development and virulence in Verticillium dahliae via S-acylation of the purine degradation enzyme VdHIUH. Using comparative S-acyl-proteomics, genetics, biochemistry, and molecular dynamics, the authors demonstrate that VdERF2-mediated S-acylation at C9 enhances VdHIUH activity, reduces uric acid accumulation, and maintains ROS homeostasis through allantoin. The work establishes a direct link between protein lipidation, central metabolism, and fungal pathogenicity. The experiments are rigorous, the data consistent, and the writing clear. The topic fits PLOS Pathogens scope and will be of broad interest.

**********

**Part II – Major Issues: Key Experiments Required for Acceptance**

Please use this section to detail the key new experiments or modifications of existing experiments that should be absolutely required to validate study conclusions.

Generally, there should be no more than 3 such required experiments or major modifications for a "Major Revision" recommendation. If more than 3 experiments are necessary to validate the study conclusions, then you are encouraged to recommend "Reject".

Reviewer #1: The authors conclude that they have uncovered a critical molecular mechanism by which S-acylation modulates purine metabolism to control pathogenicity. However, the fact that allantoin acts as an ROS scavenger to alleviate oxidative stress is already a widely recognized phenomenon across multiple biological kingdoms, including plants and microbes. Therefore, simply demonstrating that ROS suppression via purine metabolism maintains virulence may lack the high degree of novelty expected by PLOS Pathogens. The true originality of this study is strictly limited to the upstream regulatory mechanism—specifically, VdERF2-mediated S-acylation. To strengthen the manuscript's impact, the authors must discuss whether this specific S-acylation regulatory mechanism is evolutionarily conserved among other pathogenic fungi.

The experiments using comparative S-acyl-proteomic analyses to identify targets and tracing the resulting ROS accumulation defect back to VdHIUH is generally logical. However, the manuscript explicitly states that VdERF2-regulated S-acylation is significantly enriched across various metabolic pathways, not just purine metabolism. Despite this, the authors oversimplify their conclusion by attributing the severe pathogenicity defects of VdERF2 mutants entirely to the functional loss of a single target, VdHIUH. Given that VdERF2 likely possesses numerous S-acylation targets, definitively linking the entire mutant phenotype solely to VdHIUH and purine metabolism is a significant logical leap that requires further justification.

To build a more robust scientific case and address the flaws mentioned above, I strongly recommend the following improvements.

1) Direct proof of single-target dependency: To rule out interference from other metabolic pathways affected by VdERF2, the authors must demonstrate whether overexpressing an artificially active (S-acylation-independent) version of VdHIUH in a VdERF2 deletion mutant can fully rescue the ROS suppression and pathogenicity defects.

2) Mapping target cysteine residues: The authors need to provide biochemical and molecular data identifying the exact cysteine residue(s) on VdHIUH where S-acylation occurs. Point mutation analyses of these specific residues are necessary to definitively prove the structural and functional changes in enzymatic activity.

3) In planta infection stress data: The mechanism of restoring normal development and maintaining pathogenicity via ROS mitigation must be thoroughly validated under actual host infection conditions (in planta). It is critical to demonstrate that this allantoin-mediated mechanism effectively counteracts the massive oxidative burst generated by the plant's active immune response, rather than merely observing ROS reduction in an in vitro culture environment.

Reviewer #2: (No Response)

Reviewer #3: Is there any interaction between VdERF2 and VdHIUH?

**********

**Part III – Minor Issues: Editorial and Data Presentation Modifications**

Please use this section for editorial suggestions as well as relatively minor modifications of existing data that would enhance clarity.

Reviewer #1: (No Response)

Reviewer #2: (No Response)

Reviewer #3: Title: Comparable → Comparative

line 22. modulates protein property and function → properties and functions

line 31. enhances VdHIUH activity → enhances the activity of VdHIUH

line 102. This discovery would→This discovery establishes

line 107. VdERF2 is PAT essential→ VdERF2 is a PAT that is essential

line 409. Using a similar approach, the similar method→Using a similar approach

**********

PLOS authors have the option to publish the peer review history of their article (what does this mean?). If published, this will include your full peer review and any attached files.

If you choose “no”, your identity will remain anonymous but your review may still be made public.

**Do you want your identity to be public for this peer review?**  For information about this choice, including consent withdrawal, please see our Privacy Policy.

Reviewer #1: **Yes:**  Junhyun Jeon

Reviewer #2: No

Reviewer #3: No

[NOTE: If reviewer comments were submitted as an attachment file, they will be attached to this email and accessible via the submission site. Please log into your account, locate the manuscript record, and check for the action link "View Attachments". If this link does not appear, there are no attachment files.]

**Figure resubmission:**

**Reproducibility:**

?>

# Author comment: Author response to Decision Letter 1

**DOI:** 10.1371/journal.ppat.1014730.r002

12 Aug 2026

Attachment Submitted filename: Li et al Response to reviewers.docx

# Peer review: Decision Letter 1

**DOI:** 10.1371/journal.ppat.1014730.r003

**Authors:** Robert A. Cramer, Richard A Wilson

15 Sep 2026

PPATHOGENS-D-26-00375R1

Comparative S-acyl-proteomics reveals post-translational modulation of fungal purine metabolism for Verticillium wilt

PLOS Pathogens

Dear Dr. Lai,

Thank you for submitting your manuscript to PLOS Pathogens. After careful consideration, we feel that it has merit but does not fully meet PLOS Pathogens's publication criteria as it currently stands. Reviewer 1 raises some important points that can likely be addressed without too much additional work. Therefore, we invite you to submit a revised version of the manuscript that addresses these concerns.

Please submit your revised manuscript by Nov 14 2026 11:59PM. If you will need more time than this to complete your revisions, please reply to this message or contact the journal office at plospathogens@plos.org. When you're ready to submit your revision, log on to https://www.editorialmanager.com/ppathogens/ and select the 'Submissions Needing Revision' folder to locate your manuscript file.

Please include the following items when submitting your revised manuscript:

* A letter that responds to each point raised by the editor and reviewer(s). You should upload this letter as a separate file labeled 'Response to Reviewers'. This file does not need to include responses to any formatting updates and technical items listed in the 'Journal Requirements' section below.

* A marked-up copy of your manuscript that highlights changes made to the original version. You should upload this as a separate file labeled 'Revised Manuscript with Track Changes'.

* An unmarked version of your revised paper without tracked changes. You should upload this as a separate file labeled 'Manuscript'.

If you would like to make changes to your financial disclosure, competing interests statement, or data availability statement, please make these updates within the submission form at the time of resubmission. Guidelines for resubmitting your figure files are available below the reviewer comments at the end of this letter.

As the corresponding author, your ORCID iD is verified in the submission system and will appear in the published article. PLOS supports the use of ORCID, and we encourage all coauthors to register for an ORCID iD and use it as well. Please encourage your coauthors to verify their ORCID iD within the submission system before final acceptance, as unverified ORCID iDs will not appear in the published article. Only the individual author can complete the verification step; PLOS staff cannot verify ORCID iDs on behalf of authors.

We look forward to receiving your revised manuscript.

Kind regards,

Richard A Wilson

Academic Editor

PLOS Pathogens

Robert Cramer

Section Editor

PLOS Pathogens

Sumita Bhaduri-McIntosh

Editor-in-Chief

PLOS Pathogens

orcid.org/0000-0003-2946-9497

Dominique Soldati-Favre

Editor-in-Chief

PLOS Pathogens

orcid.org/0000-0003-4156-2109

**Journal Requirements:**

1. We noted that you have included an author contributions statement in your manuscript file, and it appears to differ from the information we have on file in your submission form. Please remove the statement from your manuscript and instead ensure that the full contributions of each author are acknowledged in the ""Add/EditRemove Authors"" section of our submission form.

The list of CRediT author contributions may be found here: https://journals.plos.org/plospathogens/s/authorship#loc-author-contributions

2. Modern Agro-industry Technology

Research System (2023KJ114 to CY), the Program for Changjiang Scholars (N/A to

CY), and Fund for young scholars from South China Normal University (24KJ17 to CL).

The funders had no role in study design, data collection and analysis, decision to

publish, or preparation of the manuscript.”. However, your funding information on the submission form indicates that you received funding from “Guangdong S&T Program        (2025B0202070003 to Prof. Jianbin Lai)”. Please indicate by return email the full and correct funding information for your study and confirm the order in which funding contributions should appear. Please be sure to indicate whether the funders played any role in the study design, data collection and analysis, decision to publish, or preparation of the manuscript.

3. Some material included in your submission may be copyrighted. According to PLOS’s copyright policy, authors who use figures or other material (e.g., graphics, clipart, maps) from another author or copyright holder must demonstrate or obtain permission to publish this material under the Creative Commons Attribution 4.0 International (CC BY 4.0) License used by PLOS journals. Please closely review the details of PLOS’s copyright requirements here: PLOS Licenses and Copyright. If you need to request permissions from a copyright holder, you may use PLOS's Copyright Content Permission form.

Please respond directly to this email and provide any known details concerning your material's license terms and permissions required for reuse, even if you have not yet obtained copyright permissions or are unsure of your material's copyright compatibility. Once you have responded and addressed all other outstanding technical requirements, you may resubmit your manuscript within Editorial Manager.

Potential Copyright Issues:

Figure 2: Please confirm whether you drew the images / clip-art within the figure panels by hand. If you did not draw the images, please provide (a) a link to the source of the images or icons and their license / terms of use; or (b) written permission from the copyright holder to publish the images or icons under our CC BY 4.0 license. Alternatively, you may replace the images with open source alternatives. See these open source resources you may use to replace images / clip-art:

- https://commons.wikimedia.org

- https://openclipart.org/

Fig 1, 3, S4, S9: Please confirm (a) that you are the photographer; or (b) provide written permission from the photographer to publish the photo(s) under our CC BY 4.0 license.

If the reviewer comments include a recommendation to cite specific previously published works, please review and evaluate these publications to determine whether they are relevant and should be cited. There is no requirement to cite these works unless the editor has indicated otherwise.

[Note: HTML markup is below. Please do not edit.]

**Reviewers' Comments:**

Reviewer's Responses to Questions

**Part I - Summary**

Please use this section to discuss strengths/weaknesses of study, novelty/significance, general execution and scholarship.

Reviewer #1: The authors have responded constructively to the previous round. All three experiments I listed as required for acceptance were attempted, two of them generating new data (Fig S9, Fig S10), and the third point (mapping of the acylated cysteine) was already supported by data present in the original submission, which I had overlooked. The addition of the VdERF2–VdHIUH co-immunoprecipitation (Fig S6), the systematic virulence comparison across all VdPAT mutants (Fig S4), and the cross-species conservation analyses (Fig S2, Fig S7) meaningfully strengthen the manuscript. Importantly, the authors have toned down the central causal claim, now stating that VdHIUH partially mediates the biological function of VdERF2, which directly addresses my concern about attributing the entire ΔVdERF2 phenotype to a single substrate.

Reviewer #2: I dont have any questions. I have reviewed the questions and authors' revision.

Reviewer #3: The authors have addressed my comments. I have no more questions.

**********

**Part II – Major Issues: Key Experiments Required for Acceptance**

Please use this section to detail the key new experiments or modifications of existing experiments that should be absolutely required to validate study conclusions.

Generally, there should be no more than 3 such required experiments or major modifications for a "Major Revision" recommendation. If more than 3 experiments are necessary to validate the study conclusions, then you are encouraged to recommend "Reject".

Reviewer #1: My main remaining concern is the allantoin rescue experiments. Allantoin is a major nitrogen source for filamentous fungi, catabolised via allantoinase and allantoicase to urea and ammonia. Loss of VdHIUH, or loss of its VdERF2-dependent S-acylation, blocks flux from uric acid to allantoin and therefore blocks access to purine-derived nitrogen. Under this interpretation, exogenous allantoin would restore growth of the ΔVdERF2, ΔVdHIUH and ECVdHIUH(C9S) strains purely as a nutrient, with no antioxidant contribution required. Critically, this alternative also predicts the authors' own control result — that allantoin has no effect on the wild type — because the wild type already produces allantoin endogenously. The observation that allantoin lowers H_2_;O_2_ is likewise compatible with either model, since restoring flux through the pathway would relieve uric acid accumulation regardless of mechanism.

As the data stand, the two explanations are not separable, yet the Discussion states that the allantoin rescue is "confirming that ROS accumulation is a primary cause of their abnormal development."

I would accept either of the following:

(a) A discriminating control. A single plate-based experiment would resolve this: test whether a non-metabolisable antioxidant (N-acetylcysteine, ascorbate, or reduced glutathione) reproduces the growth rescue, and/or whether an equimolar alternative nitrogen source (urea or ammonium) fails to do so. If the antioxidant rescues and the alternative nitrogen source does not, the ROS model is substantially strengthened.

(b) Explicit acknowledgement and downgrading. If the authors prefer not to perform this experiment, the nitrogen-nutrition alternative must be stated explicitly in the Discussion as an unexcluded possibility, and all causal ROS language must be softened accordingly.

Reviewer #2: (No Response)

Reviewer #3: (No Response)

**********

**Part III – Minor Issues: Editorial and Data Presentation Modifications**

Please use this section for editorial suggestions as well as relatively minor modifications of existing data that would enhance clarity.

Reviewer #1: In Fig 4F the allantoin effect is approximately 21 → 22 mm for ΔVdERF2 and 21.5 → 22.5 mm for ECVdHIUH(C9S), against a wild-type value of roughly 23 mm. The differences are statistically significant but the mutants are not restored to wild-type level. "Restore" should be replaced with "partially alleviate" throughout, including in the Abstract.

A clear residual band is visible in the C9S pulldown (+NH_2_OH) lane, and the quantification indicates roughly 70% reduction rather than near-complete loss. This residual signal is itself informative: it suggests one or more additional, minor S-acylation sites, which is directly relevant to my earlier request for identification of the acylated residue(s). Please describe the result as it appears and note the implication.

Reviewer #2: (No Response)

Reviewer #3: (No Response)

**********

PLOS authors have the option to publish the peer review history of their article (what does this mean?). If published, this will include your full peer review and any attached files.

If you choose “no”, your identity will remain anonymous but your review may still be made public.

**Do you want your identity to be public for this peer review?**  For information about this choice, including consent withdrawal, please see our Privacy Policy.

Reviewer #1: **Yes:**  Junhyun Jeon

Reviewer #2: No

Reviewer #3: No

[NOTE: If reviewer comments were submitted as an attachment file, they will be attached to this email and accessible via the submission site. Please log into your account, locate the manuscript record, and check for the action link "View Attachments". If this link does not appear, there are no attachment files.]

**Figure resubmission:**

**Reproducibility:**

To enhance the reproducibility of your results, we recommend that authors of applicable studies deposit laboratory protocols in protocols.io, where a protocol can be assigned its own identifier (DOI) such that it can be cited independently in the future. Additionally, PLOS ONE offers an option to publish peer-reviewed clinical study protocols. Read more information on sharing protocols at https://plos.org/protocols?utm_medium=editorial-email&utm_source=authorletters&utm_campaign=protocols

# Author comment: Author response to Decision Letter 2

**DOI:** 10.1371/journal.ppat.1014730.r004

17 Sep 2026

Attachment Submitted filename: Li_et_al_Response_to_Reviewers_auresp_2.docx

# Peer review: Decision Letter 2

**DOI:** 10.1371/journal.ppat.1014730.r005

**Authors:** Robert A. Cramer, Richard A Wilson

16 Oct 2026

Dear Prof. Lai,

We are pleased to inform that your manuscript, " Comparative S-acyl-proteomics reveals post-translational modulation of fungal purine metabolism for Verticillium wilt ", has been editorially accepted for publication at PLOS Pathogens.

Before your manuscript can be formally accepted and sent to production, you will need to complete our formatting changes, which you will receive by email within a week. Please note that your manuscript will not be scheduled for publication until you have made the required changes.

IMPORTANT NOTES

(1) Copyediting and Proofreading: The corresponding author will receive a typeset proof for review, to ensure errors have not been introduced during production. Please review the PDF proof of your manuscript carefully, as this is the last chance to correct any errors. Please note that major changes, or those which affect the scientific understanding of the work, will likely cause delays to the publication date of your manuscript.

(2) Appropriate Figure Files: Please remove all name and figure # text from your figure files. Please also take this time to check that your figures are of high resolution, which will improve the readbility of your figures and help expedite your manuscript's publication. Please note that figures must have been originally created at 300dpi or higher. Do not manually increase the resolution of your files. For instructions on how to properly obtain high quality images, please review our Figure Guidelines, with examples at: http://journals.plos.org/plospathogens/s/figures.

We encourage you to use PACE (http://pace.apexcovantage.com/), a figure-checking tool which helps authors ensure all figures meet PLOS requirements so that figures can publish in the highest quality. PACE is a digital diagnostic and conversion tool for figure files. It will provide information about any failed check(s) and, if able, will automatically convert the figure into an acceptable file that passes quality checks.

To use PACE, you must first register as a user. Then, login and navigate to the UPLOAD tab, where you will find detailed instructions on how to use the tool.

(3) Striking Image: Please upload a striking still image to accompany your article if one is available (you can include a new image or an existing one from within your manuscript). Should your paper be accepted, this image will be considered for our monthly issue image and may also appear on our website to feature your article. Please upload this as a separate file, selecting "striking image" as the file type upon upload. Please also include a separate "Other" file with a caption, including credits and any potential copyright information. Please do not include the caption in the main article file. If your image is from someone other than yourself, please ensure that the artist has read and agreed to the terms and conditions of the Creative Commons Attribution License at http://journals.plos.org/plospathogens/s/content-license. Please note that PLOS cannot publish copyrighted images.

(4) Press Release or Related Media: If you or your institution will be preparing press materials for this manuscript, please inform our press team in advance at plospathogens@plos.org as soon as possible. We ask that you contact us within one week to plan ahead of our fast Production schedule. If you need to know your paper's publication date for related media purposes, you must coordinate with our press team, and your manuscript will remain under a strict press embargo until the publication date and time.

(5) Update your Profile Information: Now that your manuscript has been provisionally accepted, please log into Editorial Manager and update your profile, if needed. Go to http://www.editorialmanager.com/ppathogens, log in, and click on the "Update My Information" link at the top of the page. Please update your user information to ensure an efficient production and billing process.

(6) LaTeX users only: Our staff will ask you to upload a TEX file in addition to the PDF before the paper can be sent to typesetting, so please carefully review our Latex Guidelines http://journals.plos.org/plospathogens/s/latex in the meantime.

Thank you again for supporting Open Access publishing; we are looking forward to publishing your work in PLOS Pathogens.

Best regards,

Richard A Wilson

Academic Editor

PLOS Pathogens

Robert Cramer

Section Editor

PLOS Pathogens

Dominique Soldati-Favre

Editor-in-Chief

PLOS Pathogens

***********************************************************

Reviewer's Responses to Questions

**Part I - Summary**

Please use this section to discuss strengths/weaknesses of study, novelty/significance, general execution and scholarship.<br/><br/>

Reviewer #1: The manuscript looks ready for publication.

--------------------

**Part II – Major Issues: Key Experiments Required for Acceptance**

Please use this section to detail the key new experiments or modifications of existing experiments that should be absolutely required to validate study conclusions.<br/><br/>Generally, there should be no more than 3 such required experiments or major modifications for a "Major Revision" recommendation. If more than 3 experiments are necessary to validate the study conclusions, then you are encouraged to recommend "Reject".<br/><br/>

Reviewer #1: I don't have further concerns.

--------------------

**Part III – Minor Issues: Editorial and Data Presentation Modifications**

Please use this section for editorial suggestions as well as relatively minor modifications of existing data that would enhance clarity.<br/><br/>

Reviewer #1: I don't have further concerns.

--------------------

PLOS authors have the option to publish the peer review history of their article (what does this mean?). If published, this will include your full peer review and any attached files.

If you choose “no”, your identity will remain anonymous but your review may still be made public.

**Do you want your identity to be public for this peer review?**  For information about this choice, including consent withdrawal, please see our Privacy Policy.

Reviewer #1: Yes: Junhyun Jeon

# Editor report: Acceptance letter

**DOI:** 10.1371/journal.ppat.1014730.r006

**Authors:** Robert A. Cramer, Richard A Wilson

Dear Prof. Lai,

We are delighted to inform you that your manuscript, " Comparative S-acyl-proteomics reveals post-translational modulation of fungal purine metabolism for Verticillium wilt ," has been formally accepted for publication in PLOS Pathogens.

We have now passed your article onto the PLOS Production Department who will complete the rest of the pre-publication process. All authors will receive a confirmation email upon publication.

Thank you again for supporting open-access publishing; we are looking forward to publishing your work in PLOS Pathogens.

Best regards,

Dominique Soldati-Favre

Editor-in-Chief

PLOS Pathogens
